# Chiral disubstituted piperidinyl ureas: a class of dual diacylglycerol lipase-α and ABHD6 inhibitors†
†The authors declare no competing interests.
‡
‡Electronic supplementary information (ESI) available. See DOI: 10.1039/c7md00029d


**DOI:** 10.1039/c7md00029d

**Published:** 2017-03-10

**Authors:** Hui Deng, Tom van der Wel, Richard J. B. H. N. van den Berg, Adrianus M. C. H. van den Nieuwendijk, Freek J. Janssen, Marc P. Baggelaar, Hermen S. Overkleeft, Mario van der Stelt

**Affiliations:** a Department of Molecular Physiology , Leiden Institute of Chemistry , Leiden University , Leiden , The Netherlands . Email: m.van.der.stelt@chem.leidenuniv.nl; b Department of Bio-organic Synthesis , Leiden Institute of Chemistry , Leiden University , Leiden , The Netherlands

## Abstract

Inhibitors of diacylglycerol lipases and α,β-hydrolase domain containing protein 6 (ABHD6) are potential leads for the development of therapeutic agents for metabolic and neurodegenerative disorders. Here, we report the enantioselective synthesis and structure activity relationships of triazole ureas featuring chiral, hydroxylated 2-benzylpiperidines as dual inhibitors of DAGLα and ABHD6. The chirality of the carbon bearing the C2 substituent, as well as the position of the hydroxyl (tolerated at C5, but not at C3) has profound influence on the inhibitory activity of both DAGLα and ABHD6, as established using biochemical assays and competitive activity-based protein profiling on mouse brain extracts.

## Abstract 2

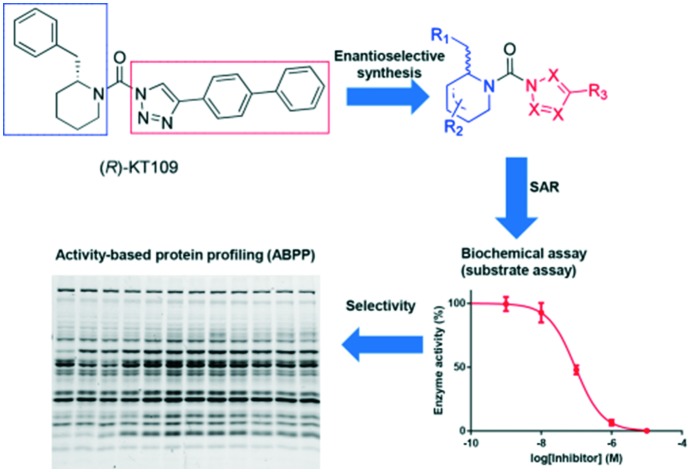
The enantioselective synthesis and structure–activity relationships of deoxy-iminosugar-based triazole ureas as dual inhibitors of DAGLα and ABHD6 were reported.

## Introduction

Diacylglycerol lipase α and diacylglycerol lipase β (DAGLα and DAGLβ) are intracellular, multi-domain, transmembrane serine hydrolases that employ a Ser-His-Asp catalytic triad to specifically hydrolyse arachidonate-containing diglycerides to form the endocannabinoid 2-arachidonoylglycerol (2-AG) in the brain and peripheral tissues.^1^,^2^ Endocannabinoid signalling is involved in various neurophysiological functions, such as learning, memory, pain sensation, adult neurogenesis and regulation of the energy balance.^3^–^5^ 2-AG is hydrolysed by monoacylglycerol lipase into arachidonic acid, which is a precursor for pro-inflammatory prostaglandins.^6^–^8^ Consequently, the development of DAGL inhibitors that perturb 2-AG production is an emerging strategy for potential therapeutic intervention in various human diseases, including metabolic syndrome related diseases and neuroinflammation.^9^,^10^

**Fig. 1:**
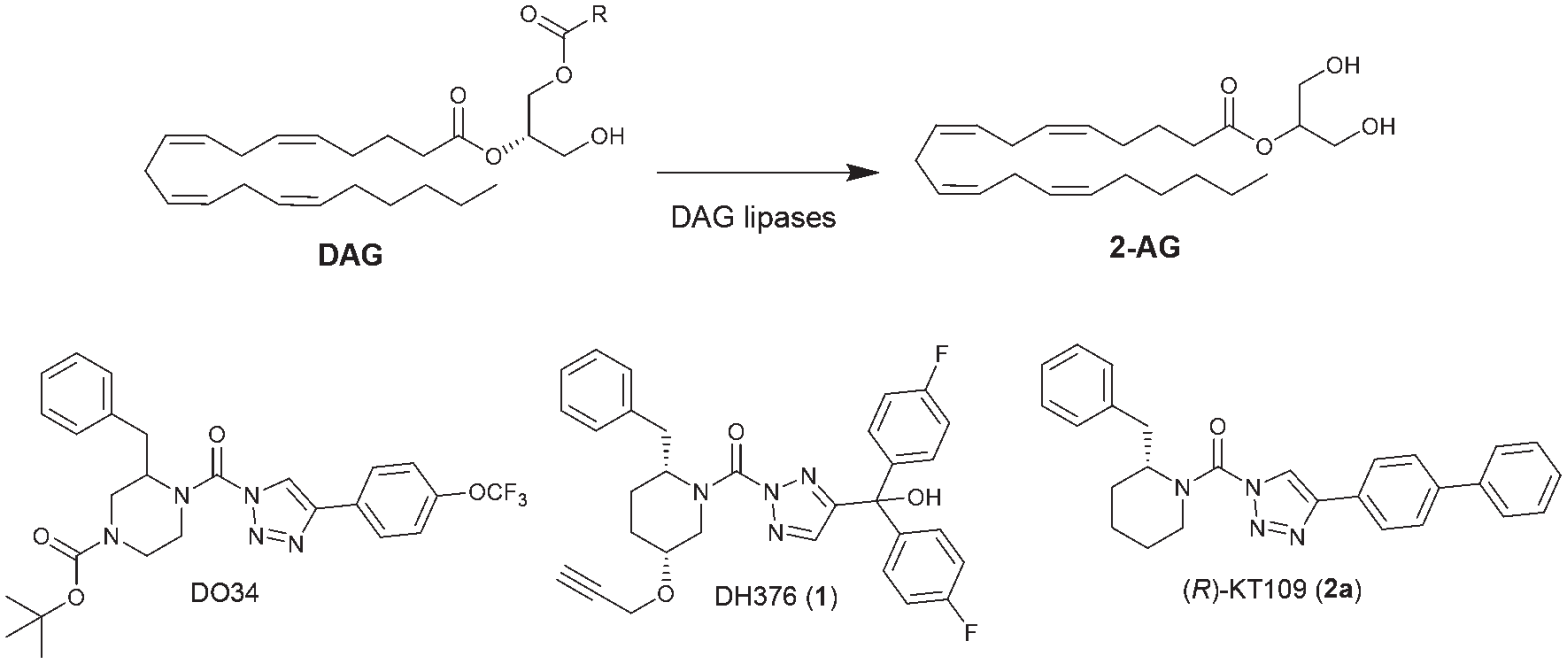
Conversion of diacylglycerol (DAG) into 2-arachidonoylglycerol (2-AG) by DAG lipases and chemical structures of their inhibitors DO34, DH376 and (*R*)-KT109.

Previously, we have reported the discovery of α-ketoheterocycles,^11^–^13^ glycinesulfonamides^14^ and triazole ureas (*e.g.* DO34 and DH376 (**1**)),^15^ as selective DAGL inhibitors (Fig. 1). DH376 and DO34 are brain active DAGL inhibitors that reduce 2-AG levels in a time- and dose-dependent manner in mouse brain. They also reduce lipopolysaccharide-induced pro-inflammatory prostaglandin and cytokine levels in mouse brain, as well as anapyrexia and refeeding in fasted mice.^15^,^30^ Of note, most DAGL inhibitors cross-react with α,β-hydrolase domain containing protein 6 (ABHD6), which has a minor role in the hydrolysis of 2-AG,^16^ degrades bis(monoacylglycero)phosphate,^17^ and acts as a lysophosphatidyl hydrolase.^18^ Inhibition of ABHD6 produces neuroprotective, anti-obesity and anti-inflammatory effects in preclinical disease models.^19^,^20^ Thus, dual inhibition of DAGLs and ABHD6 may actually be advantageous from a therapeutic point of view.

The design of DH376 and DO34 was inspired by (*R*)-KT109 (**2a**),^21^,^22^ the first *in vivo* active DAGLβ inhibitor. Both compounds are covalent irreversible inhibitors that feature a 2-benzylpiperidine moiety that confers selectivity and activity towards DAGLs and ABHD6. We have reported previously an enantioselective synthesis route for DH376 based on our ample experience with the synthesis of chiral piperidines from easily available starting materials following a strategy that encompasses enzyme-catalysed cyanohydrin synthesis followed by a transamination-reduction-ring-closing metathesis series of events.^23^–^25^

Our strategy, as we demonstrated earlier in the synthesis of polyhydroxylated piperidines (termed iminosugars), is especially suited for the construction of chiral, enantiopure 2-alkylpiperidines bearing one or more hydroxyl substituents. We realised that, in this way, piperidinylureas bearing multiple substituents, amongst which solubilizing hydroxyl groups, would be easy to accomplish. To demonstrate the validity of this reasoning, and to extend our panel of putative serine hydrolase inactivators, we set out to make a small library of chiral, disubstituted piperidinylureas. Here, these synthesis efforts as well as the inhibitory potential of the resulting compounds **3**, **4a–7a**, **4b–7b**, **6c**, **8** and **9–18**, in comparison with lead compounds **2a** and **2b** against DAGLα and ABHD6 are reported.

## Results and discussion

### Chemistry

**Fig. 2:**
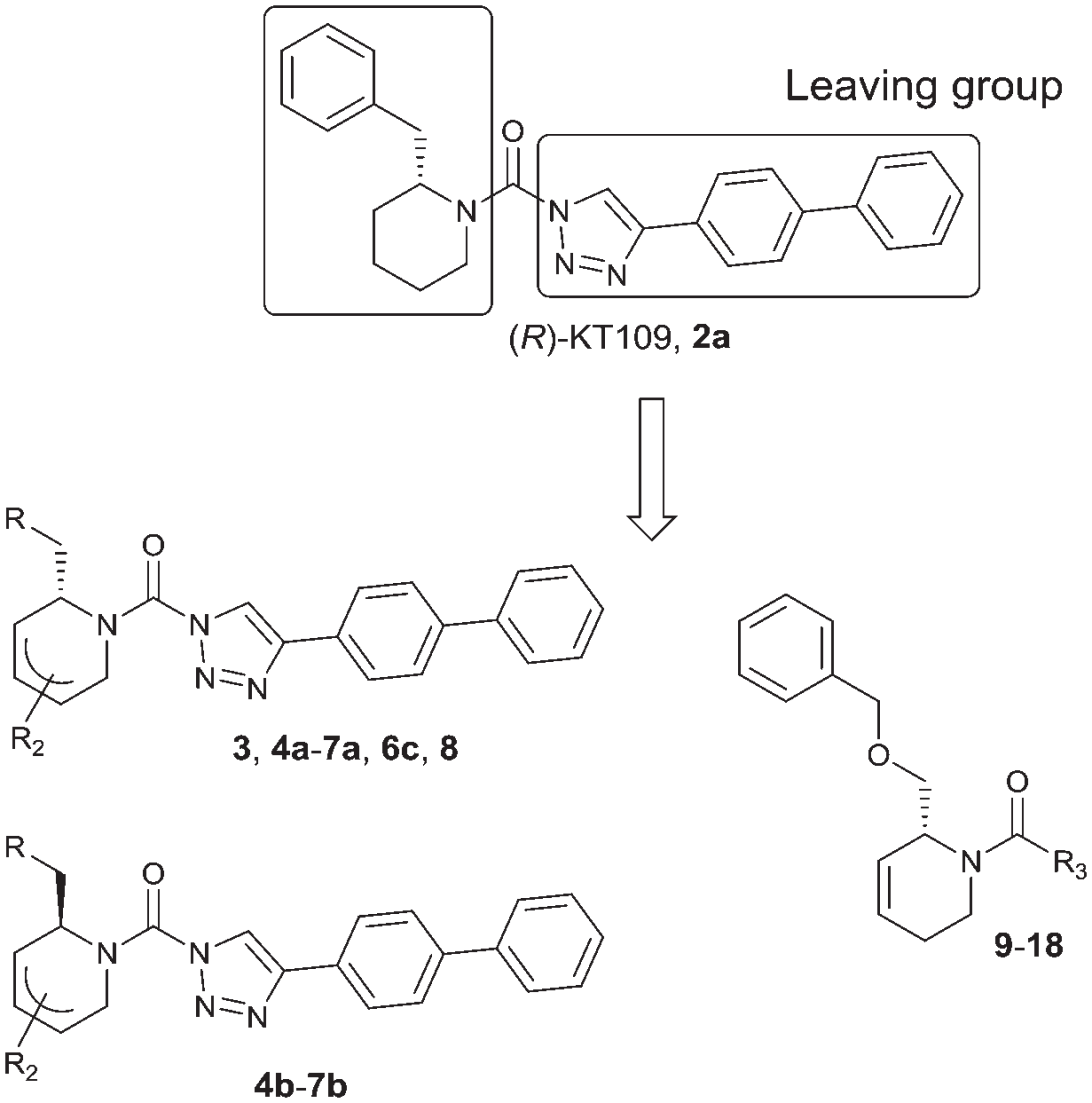
Design of compounds **3**, **4a–7a**, **4b–7b**, **6c**, **8** and **9–18** based on lead **2a**.

**Scheme 1:**
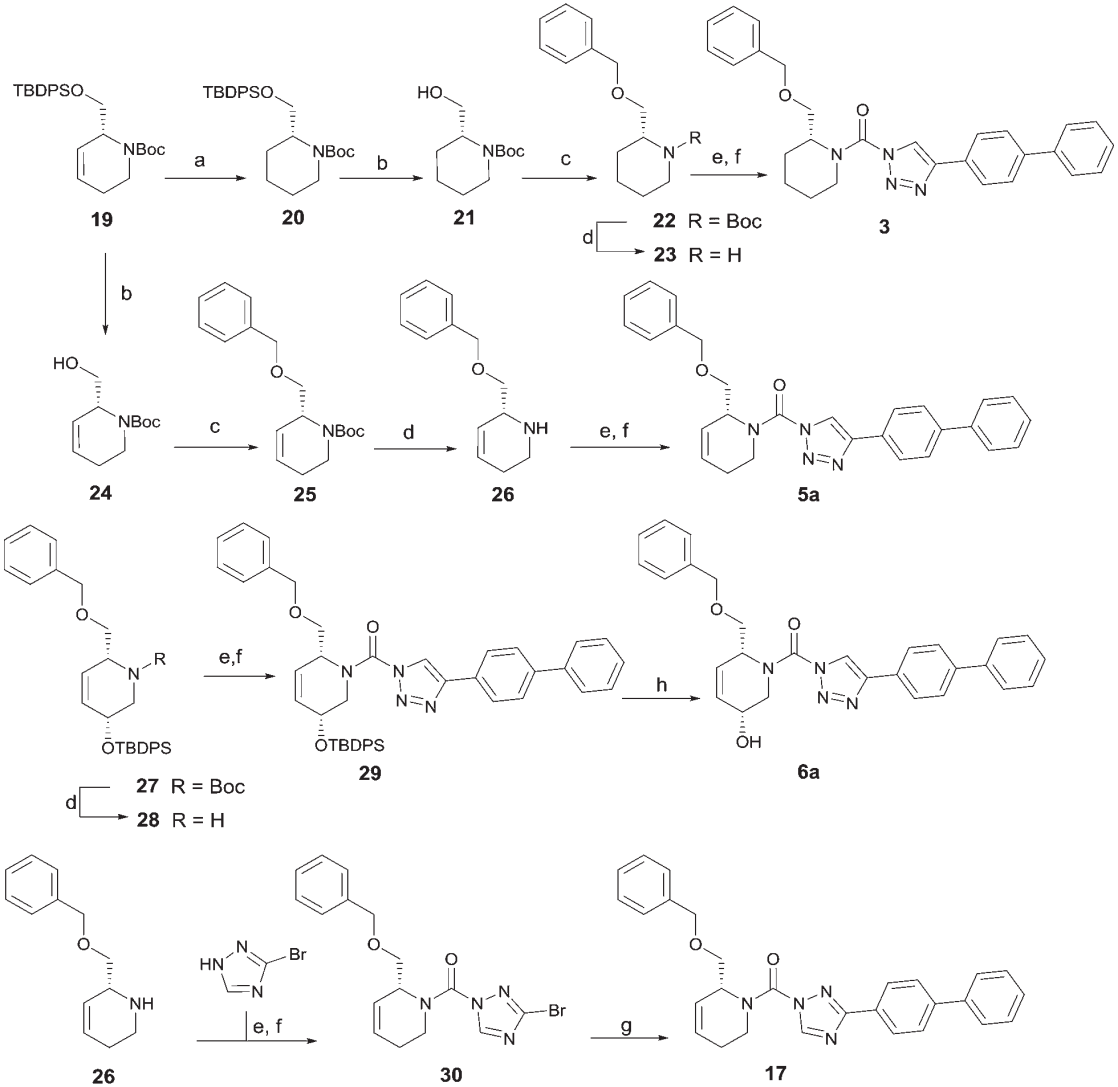
Reagents and conditions: (a) 10% Pd/C, H_2_, MeOH, 95%; (b) TBAF, THF, r.t., 98% (**21**), 92% (**24**); (c) BnBr, TBAI, NaH, DMF, 90% (**22**), 90% (**25**); (d) 25% TFA, DCM, 84% (**26**), 85% (**28**); (e) DIPEA, triphosgene, THF, 0 °C; (f) DIPEA, DMAP, 1,2,3-triazole, THF, 60 °C, 25% (**3**), 30% (**5a**), 40% (**30**) over 2 steps; (g) 1,4-dioxane : H_2_O (2 : 1), biphenyl boronic acid, PdCl_2_ (dppf), 80 °C, 75%; (h) HF-pyridine, THF : pyridine = 1 : 1 (v/v), 20% over 3 steps (based on **28**).

To systematically investigate the structure–activity relationship of the covalent irreversible inhibitors, we focused our attention first on the modification of 2-alkylpiperidine group, resulting 1,2,3-triazole ureas **3**, **4a–7a**, **4b–7b**, **6c**, **8** (Fig. 2, Tables 1 and 2). Next, we explored the influence of electrophilicity of the leaving group (*i.e.* triazole scaffold) by synthesizing compounds **9–18**. The synthesis started with compound **3**, as a close homologue of lead compound **2a** with a methyloxy moiety inserted into the benzylic position. The synthesis route commenced with *O*-TBDPS-protected intermediate **19** that was prepared according to previously established procedure.^26^ Treatment of **19** with hydrogen and 10% Pd/C in MeOH gave hydrogenated intermediate **20**, and ensuing desilylation and benzylation of the primary alcohol yielded Boc-protected intermediate **22** (Scheme 1). Removal of the Boc group using 25% (v/v) TFA in DCM gave amine **23** in near quantitative yield. Finally, triphosgene-mediated condensation of **23** with 4-([1,1′-biphenyl]-4-yl)-1*H*-1,2,3-triazole and isolation of the 1,4-regioisomer by silica gel chromatography provided compound **3** in >95% ee as determined by chiral HPLC.

**Table 1:** pIC_50_ ± SEM values of triazole ureas **3**, **4a–7a**, **4b–7b**, **6c**, and **8**. Inhibition of recombinant human DAGLα or ABHD6 was measured by indicated assays. Data represent average values ± SEM; *n* = 4 group

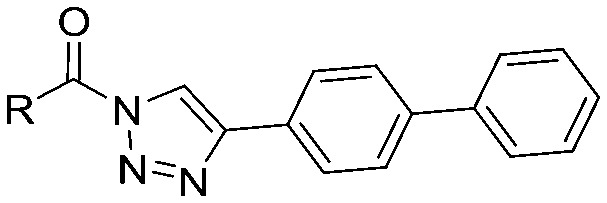							
Entry	R	pIC_50_ ± SEM (DAGLα)	pIC_50_ ± SEM (ABHD6)	Entry	R	pIC_50_ ± SEM (DAGLα)	pIC_50_ ± SEM (ABHD6)
**2a**	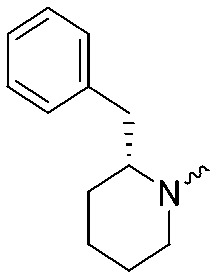	9.1 ± 0.1	8.6 ± 0.1	**2b**	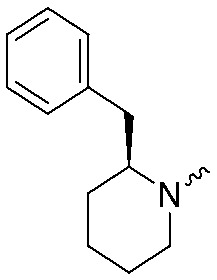	7.4 ± 0.1	6.2 ± 0.1
**3**	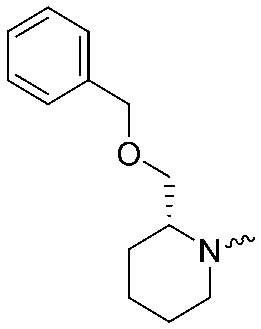	7.1 ± 0.1	8.5 ± 0.1	**8**	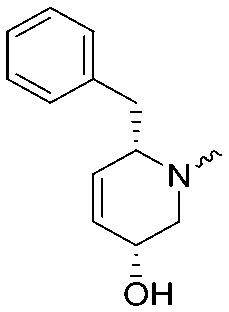	7.8 ± 0.1	8.5 ± 0.2
**4a**	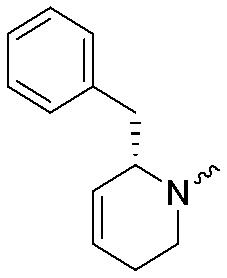	9.1 ± 0.1	8.6 ± 0.1	**4b**	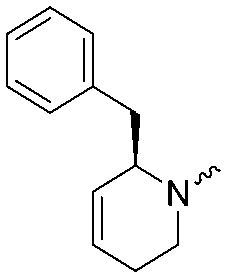	7.1 ± 0.1	7.6 ± 0.1
**5a**	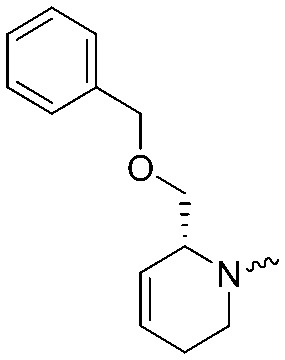	7.6 ± 0.1	7.9 ± 0.1	**5b**	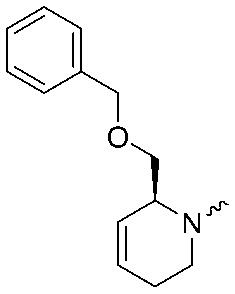	5.9 ± 0.2	7.0 ± 0.1
**6a**	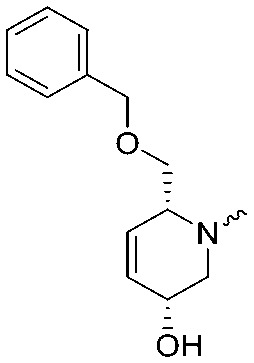	7.6 ± 0.1	8.3 ± 0.1	**6b**	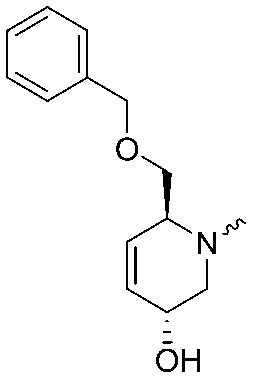	<5	6.5 ± 0.1
**6c**	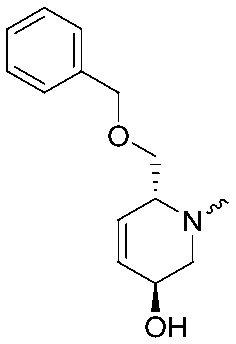	7.5 ± 0.2	8.0 ± 0.1				
**7a**	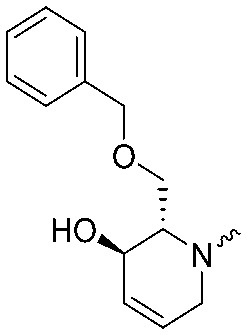	<5	6.1 ± 0.1	**7b**	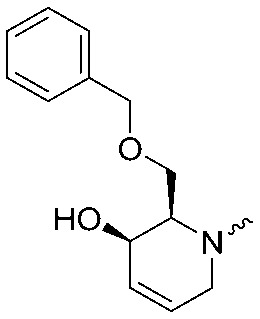	<5	6.6 ± 0.1

**Table 2:** pIC_50_ ± SEM values of compounds **9–18**. Inhibition of recombinant human DAGLα or ABHD6 was measured by indicated assays. Data represent average values ± SEM; *n* = 4 group

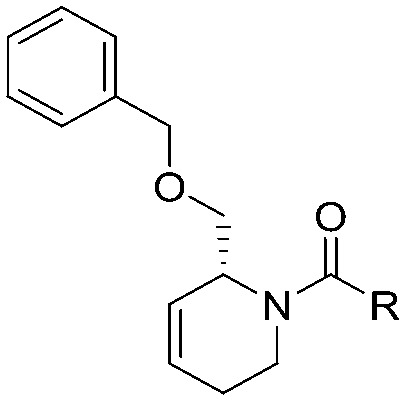			
Entry	R	pIC_50_ ± SEM (DAGLα)	pIC_50_ ± SEM (ABHD6)
**9**	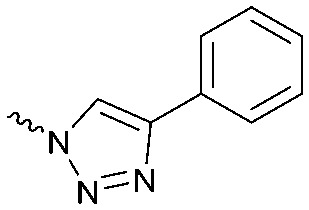	6.8 ± 0.1	6.8 ± 0.1
**10**	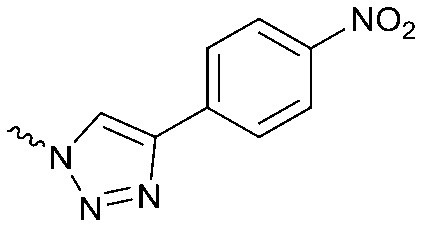	7.8 ± 0.1	7.5 ± 0.1
**11**	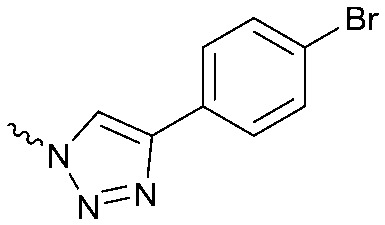	7.8 ± 0.1	7.8 ± 0.1
**12**	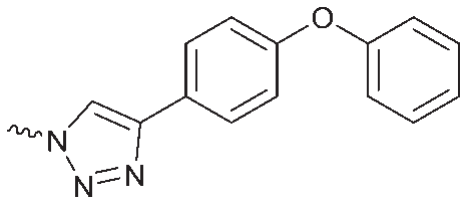	7.6 ± 0.1	8.2 ± 0.1
**13**	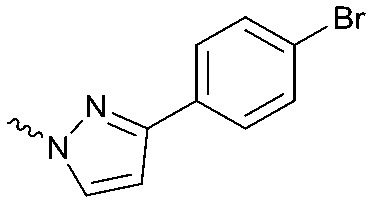	<5	<5
**14**	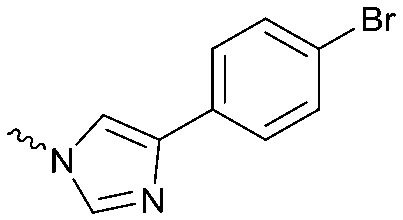	<5	<5
**15**	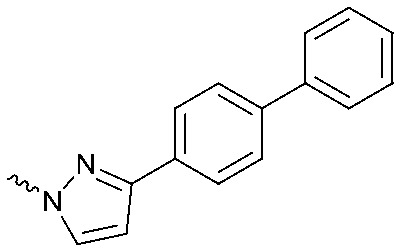	<5	<5
**16**	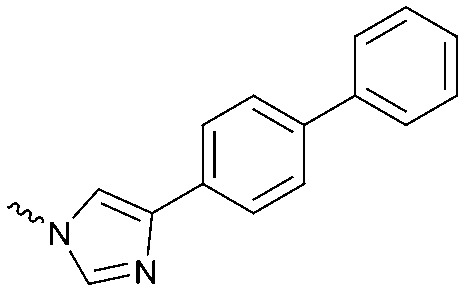	<5	<5
**17**	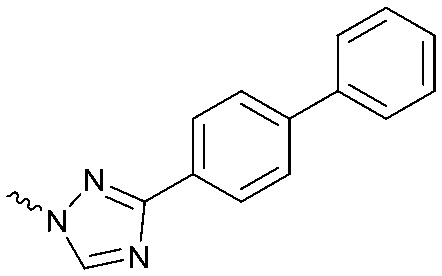	<5	<5
**18**	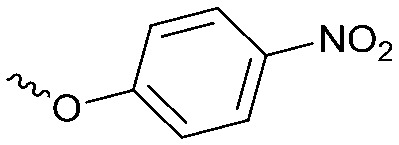	<5	<5

**Scheme 2:**
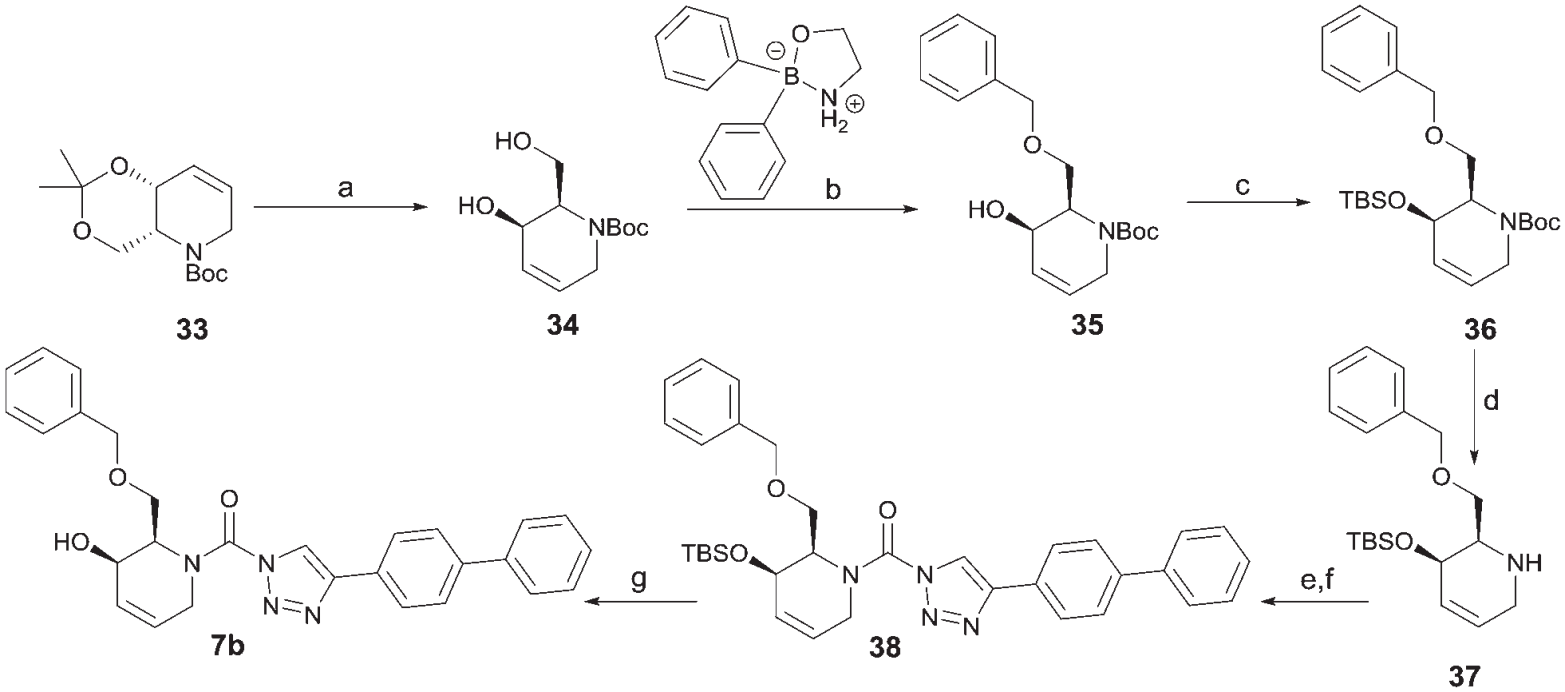
Reagents and conditions: (a) cat. *p*-TsOH, MeOH, 86%; (b) BnBr, K_2_CO_3_, KI, MeCN, 60 °C, 89%; (c) TBS-Cl, imidazole, DMF, 95%; (d) 10% TFA, DCM, 0 °C, 69%. (e) DIPEA, triphosgene, THF, 0 °C; (f) DIPEA, DMAP, 1,2,3-triazole, THF, 60 °C; (g) HF-pyridine, THF : pyridine = 1 : 1 (v/v), 15% over 3 steps.

Following a related sequence of events, we obtained compound **5a** (Scheme 1). Compound **5b** (the enantiomer of **5a**) was synthesized in the same fashion as described for **5a** (see ESI,‡ Scheme S1). For the synthesis of compound **6a**, we first prepared key intermediate **27** by employing a previously reported method.^15^,^24^ Subsequently, removal of the Boc group using 25% (v/v) TFA in DCM generated amine **28** that was directly coupled with 4-([1,1′-biphenyl]-4-yl)-1*H*-1,2,3-triazole. After silica gel chromatography, 1,4-regioisomer **29** was isolated and ensuing desilylation with HF-pyridine yielded target compound **6a** (Scheme 1). In a similar manner, we prepared compounds **4a**, **4b**, **6b**, **6c** and **8** with different stereochemistry and substitution pattern on the piperidine ring (see for synthesis details the ESI‡). The synthesis of compound **7b** started with piperidene **33** that was prepared according to previously reported method.^27^,^28^ Deprotection of **33** with a catalytic amount of *p*-TsOH yielded diol intermediate **34** that was then regioselectively benzylated using boronic amide as catalyst^29^ (Scheme 2). After *O*-silylation and *N*-Boc deprotection, we obtained free amine **37** that was successfully coupled with triazole using triphosgene. Finally, desylilation (HF-pyridine) gave target compound **7b**. Compound **7a** (diastereoisomer of **7b**) was obtained in the same fashion (see ESI‡).

Compounds **9–17** were prepared by triphosgene-mediated condensation of free amine **26** with the appropriate heterocycle. As an example, heterocycle **17** (Scheme 1) was synthesized by coupling of **26** with 3-bromo-1*H*-1,2,4-triazole followed by Suzuki coupling with (1,1′-biphenyl)-4-ylboronic acid (Scheme 1). Finally, the *para*-nitrophenyl carbamate derivative **18** was prepared following a strategy as followed for heterocycle **5a** with 4-nitrophenol instead of 4-([1,1′-biphenyl]-4-yl)-1*H*-1,2,3-triazole (see ESI‡).

### Biological evaluation

The potency of **3**, **4a–7a**, **4b–7b**, **6c**, **8** and **9–18**, as DAGLα inhibitors was established in a colorimetric assay using *para*-nitrophenylbutyrate as a surrogate substrate and membrane fractions from HEK293T cells overexpressing recombinant human DAGLα. As a reference, we report the biochemical data of (*R*)-KT109 (**2a**),^30^ which we established to be more potent than its enantiomer, (*S*)-KT109 (**2b**).^30^ The same stereochemistry at the C-2 position was preferred for compounds tested (*e.g.* compare compounds **4a–6a***vs.***4b–6b**). A 30–100-fold drop in potency of benzyloxy-containing compounds (**3** and **5a**) was found. This may suggest that a lipophilic pocket in DAGLα, which accommodates the 2-benzylpiperidine moiety, is restricted in size or, alternatively, that a polar, flexible linker is less preferred. Introduction of polar hydroxyl groups at other positions in the unsaturated piperidines (*e.g.***4a***vs.***8**) also reduced the activity over 20-fold. Of note, introduction of a chiral hydroxyl group at the C-3 position of an unsaturated piperidine ring (compounds **7a** and **7b**) abolished the activity against DAGLα (pIC_50_ < 5), whereas a hydroxyl at the C-5 position (compounds **6a** and **6c**) was allowed. This suggests that the position of the chiral hydroxyl group plays an important role in the binding site of DAGLα. However, a change in conformation of the piperidine ring induced by the double bond can also not be excluded to be responsible for the decrease in potency. Of note, the stereochemistry of the chiral hydroxyl at the C-5 position (**6a***vs.***6c**) is not important for DAGL activity, which may suggest that this functional group does not make any significant interaction in the binding pocket and may protrude into a solvent exposed region. Compounds **10–12** were equally potent as compound **5a**, but **9** showed ∼10-fold less activity. The pyrazoles (**13** and **15**), imidazoles (**14** and **16**), 1,2,4-triazole (**17**) and carbamate (**18**) were inactive. This is in line with a reduced electrophilicity of their warhead imparted by the heterocycle.^15^

**Fig. 3:**
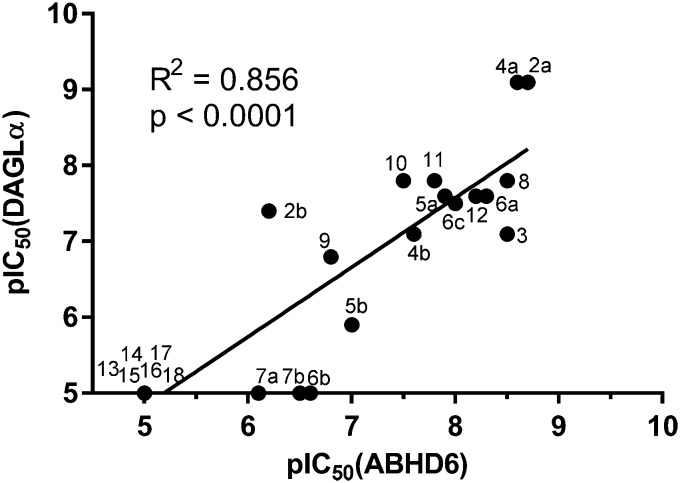
Graphical representation of DAGLα *versus* ABHD6 inhibition (pIC_50_) compounds **3**, **4a–7a**, **4b–7b**, **6c**, **8** and **9–18**.

To screen derivatives **3**, **4a–7a**, **4b–7b**, **6c**, **8** and **9–18** for ABHD6 inhibitory activity, we employed a real-time, fluorescence-based natural substrate assay with membranes from HEK293T cells expressing recombinant human ABHD6. In general, the inhibitory potency of the compounds followed the same trend as observed for DAGLα inhibition (Tables 1 and 2). To compare the DAGLα and ABHD6 activities of the compounds, we plotted their pIC_50_ values against both targets (Fig. 3). Most of the compounds were dual DAGLα/ABHD6 inhibitors and a linear relationship (*r*^2^ = 0.85) for the potency was observed. Compounds **6b**, **7a** and **7b** were inactive against DAGLα, but still showed inhibition against ABHD6 (pIC_50_ > 6). Therefore, these compounds could be interesting starting points for the discovery of selective ABHD6 inhibitors.

**Fig. 4:**
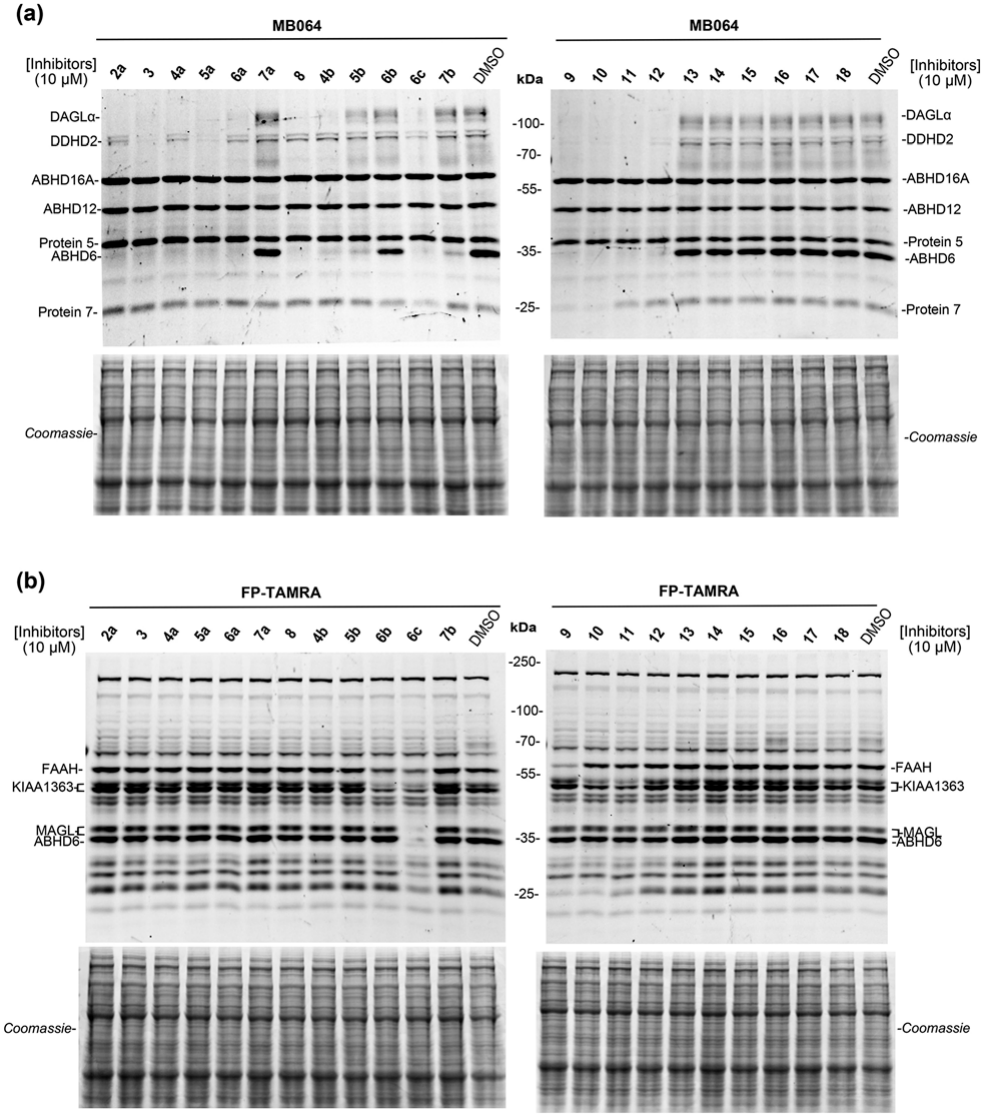
(a and b) Selectivity profile of compounds **2a**, **3**, **4a–7a**, **4b–7b**, **6c**, **8** and **9–18** (10 μM, 30 min) across mouse brain membrane serine hydrolases as determined by competitive ABPP using two broad-spectrum probes MB064 (0.25 μM, 20 min) (a) and FP-TAMRA (0.5 μM, 20 min) (b). Coomassie staining gel were used as a loading control.

Finally, to evaluate the selectivity of compounds **3**, **4a–7a**, **4b–7b**, **6c**, **8** and **9–18** across a broad panel of serine hydrolases, we applied activity-based protein profiling (ABPP) using mouse brain membrane proteome. Fluorophosphonate (FP)-based probes are routinely used in competitive ABPP experiments to determine the selectivity of serine hydrolase inhibitors.^31^,^32^ However, FP-based probes do not label DAGLα. MB064, a Bodipy-tagged tetrahydrolipstatin based β-lactone probe, was therefore previously developed by our group, to detect endogenous DAGLα in brain proteomes.^31^ Thus, we applied both TAMRA-FP and MB064 to assess the activity and selectivity of our dual DAGLα and ABHD6 inhibitors. In brief, we incubated inhibitors **3**, **4a–7a**, **4b–7b**, **6c**, **8** and **9–18** at 10 μM for 30 min with mouse brain membrane homogenates and performed a gel-based ABPP assay using MB064 (0.25 μM, 20 min) or TAMRA-FP (0.5 μM, 20 min). Almost complete blockade of DAGLα and ABHD6 was observed by compounds **3**, **4a–6a**, **4b**, **6c** and **8–12**, which is consistent with the results of the biochemical assay (Fig. 4a and Table 3). Most compounds showed excellent selectivity over the other serine hydrolases (Fig. 4). Compounds **3**, **5a**, **6c** and **9–12** did, however, reduce the labeling of DDHD2 (Fig. 4a), while compounds **6c**, **9** and **10** were non-selective and prevented the labelling of several unknown off-targets (Fig. 4a and b).

**Table 3:** Inhibitory values for compounds **2a**, **3**, **4a–7a**, **4b–7b**, **6c**, **8** and **9–18** (10 μM, 30 min) against native DAGLα and ABHD6 using competitive activity-based protein profiling (ABPP) with probe MB064 (0.25 μM, 20 min). Data represent means ± SEM, *n* = 3. Values are corrected for protein loading per lane as determined by coomassie staining

Entry	Inhibition (%)		Entry	Inhibition (%)	
	DAGLα	ABHD6		DAGLα	ABHD6
**2a**	99 ± 0	95 ± 1	**7b**	18 ± 11	83 ± 2
**3**	96 ± 1	96 ± 0	**9**	89 ± 5	89 ± 2
**4a**	99 ± 0	96 ± 1	**10**	95 ± 2	93 ± 1
**5a**	94 ± 2	96 ± 0	**11**	93 ± 3	94 ± 1
**6a**	87 ± 5	95 ± 2	**12**	93 ± 3	86 ± 2
**7a**	6 ± 13	18 ± 9	**13**	–3 ± 5	–3 ± 4
**8**	92 ± 2	96 ± 2	**14**	–9 ± 15	–15 ± 15
**4b**	83 ± 5	90 ± 3	**15**	–14 ± 18	–3 ± 19
**5b**	30 ± 12	84 ± 5	**16**	–13 ± 14	–28 ± 15
**6b**	5 ± 14	50 ± 7	**17**	0 ± 12	–16 ± 9
**6c**	85 ± 2	95 ± 2	**18**	–11 ± 12	–12 ± 9

## Conclusions

We reported the enantioselective synthesis and structure–activity relationship studies of chiral, disubstituted piperidinylureas as dual inhibitors of DAGLα and ABHD6. The SAR studies revealed the stereochemistry of the C-2 substitution on the piperidine ring plays an important role. Incorporation of a hydroxyl group at the C-5 position on piperidine ring maintained the activity against DAGLα and ABHD6, whereas a hydroxyl at the C-3 position completely abolished all DAGLα activity. Competitive activity-based protein profiling confirmed the activity of the inhibitors against endogenous DAGLα and ABHD6 and revealed differences in the selectivity profile against other serine hydrolases.

## Experimental section

### Synthesis of compound

The synthesis and characterization of all final compounds **3**, **4a–7a**, **4b–7b**, **6c**, **8**, **9–18** and intermediates are described in the ESI.‡

### Biological assays

#### Cloning procedures

DAGLα and ABHD6 constructs were obtained as reported previously.^11^ Plasmids were isolated from transformed XL-10 Z-competent cells (Maxi Prep, Qiagen) and verified by Sanger sequencing (BaseClear). The sequences were confirmed by sequence analysis at the Leiden Genome Technology Centre.

#### Cell culture and membrane preparation

Cell culture was performed as previously reported.^11^ In brief, HEK293T cells were grown in DMEM with stable glutamine and phenolred (PAA or Sigma) with 10% new born calf serum, penicillin and streptomycin. Cells were passaged every 2–3 days by resuspending in medium and seeding them to appropriate confluence. Membranes were prepared from transiently transfected HEK293T cells. One day prior to transfection 10^7^ cells were seeded in a 15 cm Petri dish. Cells were transfected by the addition of a 3 : 1 mixture of polyethyleneimine (60 μg) and plasmid DNA (20 μg) in 2 mL serum free medium. The medium was refreshed after 24 hours, and after 72 h the cells were harvested by suspending them in 20 mL medium. The suspension was centrifuged for 10 min at 1000 rpm, and the supernatant was removed. The cell pellet was stored at –80 °C until use.

Cell pellets were thawed on ice and suspended in lysis buffer A (20 mM HEPES, 2 mM DTT, 0.25 M sucrose, 1 mM MgCl_2_, 25 U mL^–1^ Benzonase). The suspension was homogenized by polytrone (3 × 7 s) and incubated for 30 min on ice. The suspension was subjected to ultracentrifugation (93.000 × *g*, 30 min, 4 °C, Beckman Coulter, Type Ti70 rotor) to yield the cytosolic fraction in the supernatant and the membrane fraction as a pellet. The pellet was resuspended in lysis buffer B (20 mM HEPES, 2 mM DTT). The protein concentration was determined with Quick Start Bradford reagent (BioRad) or Qubit™ fluorometric quantitation (Life Technologies). The protein fractions were diluted to a total protein concentration of 1 mg mL^–1^ and stored in small aliquots at –80 °C until use.

#### Biochemical DAGL activity assay

The biochemical hDAGLα assay was performed as reported previously.^11^ In brief, the biochemical hDAGLα activity assay is based on the hydrolysis of *para*-nitrophenylbutyrate (PNP-butyrate) by membrane preparations from HEK293T cells transiently transfected with hDAGLα. Reactions were performed in 50 mM pH 7.2 HEPES buffer with 0.05 μg μL^–1^ final protein concentration hDAGLα transfected protein.

#### Natural substrate based fluorescence assay (ABHD6)

The natural substrate assay was performed as reported previously.^14^,^33^ Standard assay conditions: 25 μM 2-AG, 0.2 U mL^–1^ glycerol kinase (GK), glycerol-3-phosphate oxidase (GPO) and horseradish peroxidase (HRP), 0.125 mM ATP, 10 μM Amplifu™ Red, 5% DMSO and 0.5% acetonitrile in a total volume of 200 μL. The final protein (ABHD6) concentration is 40 μg mL^–1^.

#### Preparation of mouse brain membrane proteome

Mouse brain membrane proteome preparation was performed as previously reported.^15^,^11^ In brief, mouse brains were isolated according to guidelines approved by the ethical committee of Leiden University (DEC#10095). Mouse brains were Dounce homogenized in pH 7.2 lysis buffer A (20 mM HEPES pH 7.2, 2 mM DTT, 1 mM MgCl_2_, 25 U mL^–1^ Benzonase) and incubated for 5 min on ice, followed by low speed spin (2500 × *g*, 3 min, 4 °C) to remove debris. The supernatant was subjected to ultracentrifugation (100 000 × *g*, 45 min, 4 °C, Beckman Coulter, Type Ti70 rotor) to yield the cytosolic fraction in the supernatant and the membrane fraction as a pellet. The pellet was resuspended in storage buffer B (20 mM HEPES pH 7.2, 2 mM DTT). The total protein concentration was determined with Quick Start Bradford reagent (Bio-Rad) or Qubit™ fluorometric quantitation (Life Technologies). Membranes and supernatant were flash frozen in liquid nitrogen and stored in aliquots at –80 °C until use.

#### Activity based protein profiling in mouse brain

Mouse brain proteome (2 mg mL^–1^, 19.5 μL) was incubated with DMSO or inhibitor in 0.5 μL DMSO for 30 min at r.t. and subsequently incubated with 500 nM (final concentration) ABP TAMRA-FP for 20 min at r.t. before the reaction was quenched with standard 3× Laemmli sample buffer. The gels were scanned using a ChemiDoc MP system and analyzed using Image Lab 4.1.

## Supplementary Material

Supplementary information
