# Supplementary material for: Chiral disubstituted piperidinyl ureas: a class of dual diacylglycerol lipase-α and ABHD6 inhibitors
Source: Medchemcomm. 2017 Mar 10;8(5):982–8. doi: 10.1039/c7md00029d (PMC6071720; doi:10.1039/c7md00029d)
Supplement: Supplementary file 1 [file MD-008-C7MD00029D-s001.pdf]

Electronic Supplementary Material (ESI) for MedChemComm.
This journal is © The Royal Society of Chemistry 2017

SUPPORTING INFORMATION

Chiral Disubstituted Piperidinyl Ureas: a Class of Dual Diacylglycerol Lipase-α and ABHD6 Inhibitors

Hui Deng[a], *Tom van der Wel*[a], *Richard J. B. H. N. van den Berg*[b], *Adrianus M.C.H. van den Nieuwendijk,*[b] *Freek J. Janssen*[a], *Marc P. Baggelaar*[a], *Hermen S. Overkleeft*[b] & *Mario van der Stelt*[a*]

[a] Department of Molecular Physiology, Leiden Institute of Chemistry, Leiden University, Leiden, The Netherlands

[b] Department of Bio-organic Synthesis, Leiden Institute of Chemistry, Leiden University, Leiden, The Netherlands

I. Experimental Section

General Methods S2

Synthesis and Characterization of final compounds and intermediates S2

Chiral HPLC analysis for the representative enantiomers S19

^{1}H NMR spectra and LC/MS data S21

II. References S43

I. EXPERIMENTAL SECTION

General Synthetic Methods. All reactions were performed using oven or flame-dried glassware and dry solvents. Reagents were purchased from Sigma Aldrich, Acros or Merck and used without further purification unless noted otherwise. All moisture sensitive reactions were performed under an argon atmosphere. Traces of water were removed from starting compounds by co-evaporation with toluene. ^{1}H-and ^{13}C-NMR spectra were recorded on a Bruker AV 400 MHz spectrometer at 400 (^{1}H) and 101 (^{13}C) MHz, or on a Bruker DMX-600 spectrometer 600 (^{1}H) and 150 (^{13}C) MHz using $CDCl_3$, or CD_3OD as solvent, unless stated otherwise. Chemical shift values are reported in ppm with tetramethylsilane or solvent resonance as the internal standard ($CDCl_3$, δ 7.26 for ^{1}H, δ 77.16 for ^{13}C; CD_3OD, δ 3.31 for ^{1}H, δ 49.00 for ^{13}C; $(CD_3)_2SO$, δ 2.50 for ^{1}H, δ 39.52 for ^{13}C). Data are reported as follows: chemical shifts (δ), multiplicity (s = singlet, d = doublet, dd = double doublet, td = triple doublet, t = triplet, q = quartet, m = multiplet, br = broad), coupling constants *J* (Hz), and integration. HPLC purification was performed on a preparative LC-MS system (Agilent 1200 series) with an Agilent 6130 Quadruple MS detector. High-resolution mass spectra (HRMS) were recorded on a Thermo Scientific LTQ Orbitrap XL. IR spectra were recorded on a Shimadzu FTIR-8300 and are reported in cm-1. Optical rotations were measured on a Propol automatic polarimeter (Sodium D-line, λ = 589 nm). Flash chromatography was performed using SiliCycle silica gel type SilicaFlash P60 (230 – 400 mesh). TLC analysis was performed on Merck silica gel 60/Kieselguhr F254, 0.25 mm. Compounds were visualized using either Seebach's reagent (a mixture of phosphomolybdic acid (25 g), cerium (IV) sulfate (7.5 g), H_2O (500 mL) and H_2SO_4 (25 mL)) or a $KMnO_4$ stain (K_2CO_3 (40 g), $KMnO_4$ (6 g), H_2O (600 mL) and 10% NaOH (5 mL)). Compound purity was determined by an LCQ Adventage Max (Thermo Finnigan) ion-trap spectrometer (ESI+) coupled to a Surveyor HPLC system (Thermo Finnigan) equipped with a C18 (Gemini, 4.6 mm x 50 mm, 3 μm particle size, Phenomenex) equipped with buffer A: H_2O, B: acetonitrile (MeCN) and C: 1% aqueous TFA. All final compounds were determined to be above 90% pure by this method.

(*R*)-(4-([1,1'-Biphenyl]-4-yl)-1*H*-1,2,3-triazol-1-yl)(2-((benzyloxy)methyl)piperidin-1-yl)methanone (3). A solution of (*R*)-2-((benzyloxy)methyl)piperidine (50.0 mg, 0.244 mmol) in THF was treated with DIPEA (0.128 mL, 0.731 mmol) and bis(trichloromethyl) carbonate (36.1 mg, 0.122 mmol) and the reaction mixture was stirred for 30 min at 0 °C. After that the reaction mixture was poured into water and extracted with ethyl acetate (3 x 10 mL). The organic layer was washed with water, brine, dried over $MgSO_4$, filtered, and concentrated under reduced pressure. The intermediate was dissolved in THF and DIPEA (0.128 mL, 0.731 mmol), DMAP (29.8 mg, 0.244 mmol) and 4-([1,1'-biphenyl]-4-yl)-1*H*-1,2,3-triazole (48.5 mg, 0.219 mmol) were added to the solution. The mixture was stirred for 2h at 60 °C and poured into saturated aqueous NH_4Cl solution (20 mL). The mixture was extracted with ethyl acetate (3 x 20 mL), washed with water, brine, dried over $MgSO_4$ and filtered. The solvents are removed under reduced pressure to yield the crude triazole urea as a mixture of N1- and N2-carbamoylated regioisomers (2 to 1 ratio). The N1-carbamoyl triazole was

isolated by silica gel chromatography (pentane/EtOAc 100:1 → 5:1) to afford compound **3** (27.6 mg, 0.061 mmol, 25 % yield). $[\alpha]_D^{22}$ = 58.7 (*c* = 0.3, $CHCl_3$). HRMS calculated for $C_{28}H_{28}N_4O_2$ $[M+H]^+$ 453.2285, found: 453.2286. ^1H NMR (400 MHz, $CDCl_3$) δ 8.08 (br, 1H), 7.87 (d, *J* = 7.7 Hz, 2H), 7.69 – 7.59 (m, 4H), 7.49 – 7.43 (m, 2H), 7.41 – 7.23 (m, 6H), 4.83 (br, 1H), 4.46 (br, 2H), 4.25 (d, *J* = 6.0 Hz, 1H), 3.86 (t, *J* = 9.6 Hz, 1H), 3.44 (br, 1H), 3.15 (br, 1H), 1.98 – 1.53 (m, 6H). ^{13}C NMR (101 MHz, $CDCl_3$) δ 151.44, 146.48, 141.36, 140.63, 137.93, 128.98, 128.84, 128.59, 127.95, 127.86, 127.68, 127.65, 127.14, 126.37, 121.21, 73.30, 68.05, 53.34, 41.93, 25.67, 25.22, 19.58.

(*S*)-(4-([1,1'-Biphenyl]-4-yl)-1*H*-1,2,3-triazol-1-yl)(6-benzyl-3,6-dihydropyridin-1(2*H*)-yl)methanone (4a). The title compound was synthesized from (*S*)-6-benzyl-1,2,3,6-tetrahydropyridine (43.0 mg, 0.248 mmol) according to the procedures described for compound **3**. This furnished compound **4a** (34.8 mg, 0.083 mmol, 33 % yield). $[\alpha]_D^{22}$ = 59.6 (*c* = 0.4, $CHCl_3$). HRMS calculated for $C_{27}H_{25}N_4O$ $[M+H]^+$ 421.2023, found: 421.2021. ^1H NMR ($CDCl_3$, 600 MHz, mixture of two rotamers ratio A/B = 53/47) Major rotamer: δ 8.36 (br, 0.5H), 7.95 – 7.87 (m, 2H), 7.77 (br, 0.5H) 7.69 (d, *J* = 5.2 Hz, 2H), 7.64 (d, *J* = 4.8 Hz, 2H), 7.50 – 7.43 (m, 2H), 7.38 – 7.28 (m, 3H), 7.25 – 7.12 (m, 3H), 5.97 – 5.93 (m, 1H), 5.69 – 5.63 (m, 1H), 5.37 (br, 0.5H), 4.93 (br, 0.5H), 4.54 (dd, *J* = 4.0, 12.0 Hz, 1H), 3.29 – 3.26 (m, 1H), 3.23 – 3.18 (m, 1H), 3.05 – 2.90 (m, 1H), 2.65 – 2.51 (m, 1H), 2.23 – 2.11 (m, 1H). ^{13}C NMR ($CDCl_3$, 151 MHz) Major rotamer: δ 151.36, 148.86, 146.60, 141.58, 140.59, 137.18, 129.74, 129.01, 128.82, 128.66, 127.77, 127.69, 127.14, 126.94, 126.40, 125.75, 120.93, 57.43, 41.94, 40.95, 25.82.

(*R*)-(4-([1,1'-Biphenyl]-4-yl)-1*H*-1,2,3-triazol-1-yl)(6-((benzyloxy)methyl)-3,6-dihydropyridin-1(2*H*)-yl)methanone (5a). The title compound was synthesized from (*R*)-6-((benzyloxy)methyl)-1,2,3,6-tetrahydropyridine (50.0 mg, 0.246 mmol) according to the procedures described for compound **3**. This furnished compound **5a** (33.2 mg, 0.074 mmol, 30% yield). $[\alpha]_D^{22}$ = 146.5 (*c* = 0.4, $CHCl_3$). The enantiomeric purity was determined on a Daicel Chiralcel OD-H column (4.5 X 250 mm, 20:80 IPA/Hex, flow rate of 1 mL/min): 23.2 min, e.e.>96%. HRMS calculated for $C_{28}H_{26}N_4O_2$ $[M+H]^+$ 451.2129, found: 451.2130. ^1H NMR (400 MHz, $CDCl_3$) δ 8.23 (br, 1H), 7.87 (br, 2H), 7.87 – 7.62 (m, 4H), 7.48 – 7.44 (m, 2H), 7.40 – 7.34 (m, 1H), 7.34 – 7.24 (m, 5H), 6.05 (br, 1H), 5.71 (br, 1H), 5.34 (br, 0.5H), 4.96 (br, 0.5H), 4.51 (br, 3H), 3.86 – 3.32 (m, 3H), 2.59 (br, 1H), 2.16 (br, 1H). ^{13}C NMR (151 MHz, $CDCl_3$) δ 151.76, 146.42, 141.45, 140.58, 137.83, 128.98, 128.68, 128.56, 127.88, 127.79, 127.68, 127.61, 127.35, 127.13, 126.40, 124.90, 121.31, 73.39, 71.00, 55.65, 39.06, 25.77.

(4-([1,1'-Biphenyl]-4-yl)-1*H*-1,2,3-triazol-1-yl)(*(3R,6R)*-6-((benzyloxy)methyl)-3-hydroxy-3,6-dihydropyridin-1(2*H*)-yl)methanone (6a). A solution of (*3R*,*6R*)-6-((benzyloxy)methyl)-3-((*tert*-butyldiphenylsilyl)oxy)-1,2,3,6-tetrahydropyridine **37** (200 mg, 0.437 mmol) in THF was treated with DIPEA (0.229 mL, 1.31 mmol) and bis(trichloromethyl) carbonate (64.8 mg, 0.218 mmol) and the reaction mixture was stirred for 30 min at 0 ^oC. The mixture was poured into water and extracted with ethyl acetate (3 x 30 mL). The organic layer was washed with water, brine dried over $MgSO_4$, filtered and concentrated under reduced pressure. The intermediate was dissolved in THF and DIPEA (0.229 mL, 1.31 mmol), DMAP (53.4 mg, 0.437 mmol) and 4-([1,1'-biphenyl]-4-yl)-1*H*-1,2,3-triazole (106 mg, 0.481 mmol) were added to the solution. The

mixture was stirred for 2h at 60 °C and poured into saturated aqueous NH_4Cl solution. The mixture was extracted with ethyl acetate, washed with water, brine, dried over $MgSO_4$, filtered and concentrated under reduced pressure. The N1-carbamoyl triazole urea **29** was isolated by silica gel chromatography (1-10% ethyl acetate/pentane) as top TLC spot. HF-Pyridine (0.235 mL, 2.61 mmol) was subsequently added to a solution of N1-carbamoyl triazole urea in THF and pyridine (1:1; 2 mL) with ice cooling, and the reaction mixture was stirred over night at room temperature. The mixture was diluted with ethyl acetate (40 mL), and then washed with $NaHCO_3$, brine, dried with $MgSO_4$, and concentrated under reduced pressure. Purification by flash chromatography to furnish compound **6a** (40 mg, 0.086 mmol, 20% yield). $[\alpha]_D^{22}$ = 7.2 (*c* = 1.4, $CHCl_3$). HRMS calculated for $C_{28}H_{26}N_4O_3$ $[M+H]^+$ 467.2078, found: 467.2078. ^1H NMR (400 MHz, $CDCl_3$) δ 8.26 (br, 1H), 7.88 (d, *J* = 4.8 Hz, 2H), 7.68 (d, *J* = 8.3 Hz, 2H), 7.66 – 7.59 (m, 2H), 7.48 – 7.43 (m, 2H), 7.39 – 7.34 (m, 1H), 7.34 – 7.23 (m, 5H), 6.04 (d, *J* = 10.4 Hz, 1H), 5.81 (br, 1H), 4.65 (d, *J* = 8.3 Hz, 2H), 4.51 (br, 2H), 3.76 (br, 2H), 3.24 (br, 1H), 2.51 (br, 1H). ^{13}C NMR (101 MHz, $CDCl_3$) δ 150.61, 146.51, 141.53, 140.41, 137.57, 132.51, 128.89, 128.50, 128.32, 127.88, 127.75, 127.65, 127.62, 127.03, 126.34, 125.55, 121.18, 73.40, 70.02, 63.86, 51.29, 36.61.

(4-([1,1'-Biphenyl]-4-yl)-1*H*-1,2,3-triazol-1-yl)(*(2S,3R)*-2-((benzyloxy)methyl)-3-hydroxy-3,6-dihydropyridin-1(2*H*)-yl)methanone (7a). The title compound was synthesized from (*2S,3R*)-2-((benzyloxy)methyl)-3-((*tert*-butyldimethylsilyl)oxy)-1,2,3,6-tetrahydropyridine (30.0 mg, 0.09 mmol) and 4-([1,1'-biphenyl]-4-yl)-1*H*-1,2,3-triazole (20.0 mg, 0.09 mmol), according to the procedures described for compound **6a**. This furnished compound **7a** (6.2 mg, 0.013 mmol, 15% yield). $[\alpha]_D^{22}$ = 8.13 (*c* = 0.2, $CHCl_3$). HRMS calculated for $C_{28}H_{26}N_4O_3$ $[M+H]^+$ 467.2078, found: 467.2077. ^1H NMR (600 MHz, $CDCl_3$) δ 8.15 (s, 1H), 7.94 (d, *J* = 8.3 Hz, 2H), 7.70 (d, *J* = 8.4 Hz, 2H), 7.64 (d, *J* = 7.1 Hz, 2H), 7.49 – 7.45 (m, 2H), 7.42 – 7.37 (m, 1H), 7.34 – 7.28 (m, 5H), 6.07 – 6.01 (m, 1H), 5.95 (br, 1H), 4.82 (br, 1H), 4.56 – 4.38 (m, 3H), 4.12 (br, 1H), 3.81 (br, 1H), 3.56 (br, 1H), 3.42 (br, 1H). ^{13}C NMR (151 MHz, $CDCl_3$) δ 151.24, 149.41, 142.62, 140.33, 137.56, 133.90, 129.05, 128.62, 128.02, 127.93, 127.89, 127.82, 127.65, 127.20, 127.11, 126.32, 126.27, 73.26, 67.00, 63.89, 59.94, 42.01.

(4-([1,1'-Biphenyl]-4-yl)-1*H*-1,2,3-triazol-1-yl)(*(3R,6S)*-6-benzyl-3-hydroxy-3,6-dihydropyridin-1(2*H*)-yl)methanone (8). The title compound was synthesized from (*3R,6S*)-6-benzyl-1,2,3,6-tetrahydropyridin-3-ol (80.0 mg, 0.187 mmol) and 4-([1,1'-biphenyl]-4-yl)-1*H*-1,2,3-triazole (41.4 mg, 0.187 mmol) according to the procedure described for compound **6a**. This furnished compound **8** (13.0 mg, 0.030 mmol, 16% yield). $[\alpha]_D^{20}$ = 3.70 (*c* = 1.0, $CHCl_3$). HRMS calculated for $C_{27}H_{24}N_4O_2$ $[M+H]^+$ 437.1972, found: 437.1971. ^1H NMR (400 MHz, $CDCl_3$) δ 8.38 (br, 1H), 7.90 (br, 2H), 7.70 (d, *J* = 8.3 Hz, 2H), 7.64 (d, *J* = 8.5 Hz, 2H), 7.50 – 7.45 (m, 2H), 7.41 – 7.14 (m, 6H), 5.93 (d, *J* = 11.7 Hz, 1H), 5.72 (dd, *J* = 10.4, 3.7 Hz, 1H), 5.41 (br, 0.4H), 4.86 (br, 0.6H), 4.70 (dd, *J* = 12.9, 5.1 Hz, 2H), 3.25 (dd, *J* = 13.0, 6.5 Hz, 1H), 3.12 – 2.93 (m, 2H). ^{13}C NMR (101 MHz, $CDCl_3$) δ 151.05, 146.48, 141.68, 140.52, 136.85, 131.10, 130.89, 129.58, 129.01, 128.85, 128.41, 127.81, 127.75, 127.16, 127.03, 126.43, 121.01, 64.19, 56.74, 47.83, 46.24.

(*R*)-(4-([1,1'-Biphenyl]-4-yl)-1*H*-1,2,3-triazol-1-yl)(6-benzyl-3,6-dihydropyridin-

1(2*H*)-yl)methanone (4b). The title compound was synthesized from (*R*)-6-benzyl-1,2,3,6-tetrahydropyridine (75.0 mg, 0.433 mmol) according to the procedure described for compound **3**. This furnished compound **4b** (58.3 mg, 0.139 mmol, 32% yield). $[\alpha]_D^{22}$ = -75.20 (c = 0.5, $CHCl_3$). HRMS calculated for $C_{27}H_{24}N_4O$ $[M+H]^+$ 421.2023, found: 421.2021. ^1H NMR (500 MHz, $CDCl_3$, mixture of two rotamers ratio A/B = 56/44) Major rotamer: δ 8.36 (br, 0.5H), 7.97 – 7.87 (m, 2H), 7.69 (d, J = 8.0 Hz, 2H), 7.64 (d, J = 4.0 Hz, 2H), 7.48 – 7.40 (m, 2H), 7.38 – 7.28 (m, 3H), 7.25 – 7.11 (m, 3H), 5.95 – 5.92 (m, 1H), 5.66 – 5.63 (m, 1H), 5.37 (br, 0.5H), 4.93 (br, 0.5H), 4.54 (dd, J = 4.0, 12.0 Hz, 1H), 3.30 – 3.26 (m, 1H), 3.23 – 3.18 (m, 1H), 3.06 – 2.98 (m, 1H), 2.61 – 2.49 (m, 1H), 2.22 – 2.11 (m, 1H). ^{13}C NMR (126 MHz, $CDCl_3$) Major rotamer: δ 151.55, 148.86, 146.59, 141. 63, 140.57, 137.21, 129.72, 128.98, 128.81, 128.64, 127.74, 127.68, 127.13, 126.67, 126.36, 125.73, 120.92, 57.39, 41.95, 39.73, 25.79.

(*S*)-(4-([1,1'-Biphenyl]-4-yl)-1*H*-1,2,3-triazol-1-yl)(6-((benzyloxy)methyl)-3,6-dihydropyridin-1(2*H*)-yl)methanone (5b). The title compound was synthesized from (*S*)-6-((benzyloxy)methyl)-1,2,3,6-tetrahydropyridine (50.0 mg, 0.251 mmol) according to the procedures described for compound **3**. This furnished compound **5b** (31.1 mg, 0.069 mmol, 28% yield). $[\alpha]_D^{22}$ = -154.0 (c = 0.8, $CHCl_3$). The enantiomeric purity was determined on a Daicel Chiralcel OD-H column (4.6 X 250 mm, 20:80 IPA/Hex, flow rate of 1 mL/min): 15.6 min, e.e.>95%. HRMS calculated for $C_{28}H_{26}N_4O_2$ $[M+H]^+$ 451.2129, found: 451.2128. ^1H NMR (400 MHz, $CDCl_3$): δ 8.37 (br, 1H), 7.87 (br, 2H), 7.69 – 7.63 (m, 4H), 7.48 – 7.44 (m, 2H), 7.40 – 7.32 (m, 1H), 7.32 – 7.15 (m, 5H), 6.11 – 5.97 (m, 1H), 5.71 (br, 1H), 5.32 (br, 0.5H), 4.96 (br, 0.5H), 4.49 (br, 3H), 3.73 (br, 2H), 3.32 (br, 1H), 2.56 (br, 1H), 2.15 (br, 1H). ^{13}C NMR (101 MHz, $CDCl_3$) δ 151.07, 146.41, 141.42, 140.55, 137.88, 128.96, 128.67, 128.53, 127.85, 127.77, 127.65, 127.56, 127.33, 127.11, 126.38, 124.88, 121.23, 73.37, 70.84, 55.16, 38.82, 24.95.

(4-([1,1'-Biphenyl]-4-yl)-1*H*-1,2,3-triazol-1-yl)((*3R,6S*)-6-((benzyloxy)methyl)-3-hydroxy-3,6-dihydropyridin-1(2*H*)-yl)methanone (**6b**). The title compound was synthesized from (*3R,6S*)-6-((benzyloxy)methyl)-3-((tert-butyldiphenylsilyl)oxy)-1,2,3,6-tetrahydropyridine (100 mg, 0.221 mmol) according to the procedure described for compound **6a**. This furnished compound **6b** (16.3 mg, 0.035 mmol, 16% yield). $[\alpha]_D^{22}$ = -144.2 (c = 0.7, $CHCl_3$). HRMS calculated for $C_{28}H_{26}N_4O_3$ $[M+H]^+$ 467.2078, found: 467.2077. ^1H NMR (400 MHz, $CDCl_3$) δ 8.38 (br, 0.5H), 8.05 (br, 0.5H), 7.85 (br, 2H), 7.71 – 7.56 (m, 4H), 7.50 – 7.44 (m, 2H), 7.41 – 7.15 (m, 6H), 6.23 – 6.16 (m, 1H), 5.90 (br, 1H), 5.41 (br, 0.4H), 5.07 (br, 0.6H), 4.70 – 4.35 (m, 3H), 4.26 (d, J = 5.4 Hz, 1H), 3.86 – 3.49 (m, 3H). ^{13}C NMR (101 MHz, $CDCl_3$) δ 150.99, 146.77, 141.67, 140.49, 137.70, 134.90, 129.03, 128.99, 128.61, 128.40, 127.98, 127.89, 127.71, 127.17, 127.12, 126.38, 121.31, 73.48, 69.80, 62.49, 54.20, 49.40.

(4-([1,1'-Biphenyl]-4-yl)-1*H*-1,2,3-triazol-1-yl)((*3S*,*6R*)-6-((benzyloxy)methyl)-3-hydroxy-3,6-dihydropyridin-1(2*H*)-yl)methanone (6c). The title compound was synthesized from (*3S,6R*)-6-((benzyloxy)methyl)-3-((*tert*-butyldimethylsilyl)oxy)-1,2,3,6-tetrahydropyridine (82.0 mg, 0.246 mmol) according to the procedures described for compound **6a** This furnished compound **6c** (19.6 mg, 0.042 mmol, 17% yield). $[\alpha]_D^{22}$ = -142.7 (c = 0.2, $CHCl_3$). HRMS calculated for $C_{28}H_{26}N_4O_3$ $[M+H]^+$ 467.2078, found: 467.2079. ^1H NMR (400 MHz, $CDCl_3$) δ 8.39 (br, 1H), 7.94 – 7.78 (m, 2H), 7.71 – 7.60

(m, 4H), 7.48 – 7.42 (m, 2H), 7.41 – 7.23 (m, 6H), 6.25 – 6.16 (m, 1H), 5.90 (br, 1H), 5.41 (br, 0.4H), 5.05 (br, 0.6H), 4.62 (br, 2H), 4.40 (br, 1H), 4.27 (d, *J* = 5.4 Hz, 1H), 3.91 – 3.48 (m, 3H). ^{13}C NMR (101 MHz, $CDCl_3$) δ 151.91, 146.74, 141.64, 140.51, 137.69, 135.37, 133.11, 128.99, 128.62, 128.43, 128.35, 128.00, 127.79, 127.72, 127.13, 126.41, 121.35, 73.50, 69.80, 62.59, 54.33, 49.93.

(4-([1,1'-Biphenyl]-4-yl)-1*H*-1,2,3-triazol-1-yl)((*2R,3R*)-2-((benzyloxy)methyl)-3-hydroxy-3,6-dihydropyridin-1(2*H*)-yl)methanone (7b). The title compound was synthesized from (*2R,3R*)-2-((benzyloxy)methyl)-3-((*tert*-butyldimethylsilyl)oxy)-1,2,3,6-tetrahydropyridine **37** (30.0 mg, 0.090 mmol) according to the procedures described for compound **6a**. The N1-carbamoyl triazole urea **38** was obtained and furnished final compound **7b** (6.6 mg, 0.014 mmol, 15% yield). $[\alpha]_D^{22}$ = -17.4 (*c* = 0.4, $CHCl_3$). HRMS calculated for $C_{28}H_{26}N_4O_3$ $[M+H]^+$ 467.2077, found: 467.2078. ^{1}H NMR (600 MHz, $CDCl_3$) δ 8.12 (s, 1H), 7.95 (d, *J* = 8.4 Hz, 2H), 7.70 (d, *J* = 8.4 Hz, 2H), 7.66 – 7.62 (m, 2H), 7.50 – 7.45 (m, 2H), 7.42 – 7.35 (m, 1H), 7.34 – 7.27 (m, 5H), 5.84 (d, *J* = 10.4 Hz, 1H), 5.74 (br, 1H), 5.10 – 4.85 (m, 2H), 4.52 (br, 2H), 4.29 (br, 1H), 3.90 – 3.76 (m, 3H). ^{13}C NMR (151 MHz, $CDCl_3$) δ 150.36, 149.33, 142.49, 140.36, 137.59, 133.79, 129.05, 128.96, 128.61, 127.98, 127.94, 127.91, 127.83, 127.82, 127.20, 127.10, 123.48, 73.46, 65.95, 65.83, 56.73, 42.07.

(*R*)-(6-((Benzyloxy)methyl)-3,6-dihydropyridin-1(2*H*)-yl)(4-phenyl-1*H*-1,2,3-triazol-1-yl)methanone (9). The title compound was synthesized from (*R*)-6-((benzyloxy)methyl)-1,2,3,6-tetrahydropyridine (70.0 mg, 0.344 mmol) and 4-phenyl-1*H*-1,2,3-triazole (55.0 mg, 0.379 mmol) according to the procedures described for compound **3**. This furnished compound **9** (45.1 mg, 0.121 mmol, 35% yield). $[\alpha]_D^{20}$ = 125.1 (*c* = 1.0, $CHCl_3$). HRMS calculated for $C_{22}H_{22}N_4O_2$ $[M+H]^+$ 375.1816, found: 375.1815. ^{1}H NMR (400 MHz, $CDCl_3$) δ 8.20 (br, 1H), 7.80 (br, 2H), 7.46 – 7.40 (m, 2H), 7.35 – 7.17 (m, 6H), 6.03 (s, 1H), 5.68 (br, 1H), 5.30 (br, 0.5H), 4.96 (br, 0.5H), 4.67 – 4.30 (m, 3H), 3.85 – 3.20 (m, 3H), 2.53 (br, 1H), 2.16 (br, 1H). ^{13}C NMR (101 MHz, $CDCl_3$) δ 146.66, 137.81, 129.70, 128.98, 128.67, 128.49, 127.82, 127.74, 127.53, 125.96, 125.63, 124.86, 121.23, 73.33, 70.55, 55.37, 42.72, 24.87.

(*R*)-(6-((Benzyloxy)methyl)-3,6-dihydropyridin-1(2*H*)-yl)(4-(4-nitrophenyl)-1*H*-1,2,3-triazol-1-yl)methanone (10). The title compound was synthesized from (*R*)-6-((benzyloxy)methyl)-1,2,3,6-tetrahydropyridine (70.0 mg, 0.344 mmol) and 4-(4-nitrophenyl)-1*H*-1,2,3-triazole (72.0 mg, 0.379 mmol) according to the procedure described for compound **3**. This furnished compound **10** (54.9 mg, 0.13 mmol, 38% yield). $[\alpha]_D^{22}$ = 123 (*c* = 0.9, $CHCl_3$). HRMS calculated for $C_{22}H_{21}N_5O_4$ $[M+H]^+$. 420.1666, found: 420.1666. ^{1}H NMR (400 MHz, $CDCl_3$) δ 8.38 – 8.21 (m, 2H), 8.18 – 7.81 (m, 3H), 7.36 - 7.21 (m, 5H), 6.08 – 6.04 (m, 1H), 5.67 (br, 1H), 5.28 (br, 0.5H), 4.97 (br, 0.5H), 4.71 – 4.32 (m, 3H), 3.85 – 3.20 (m, 3H), 2.54 (br, 1H), 2.22 (br, 1H). ^{13}C NMR (101 MHz, $CDCl_3$) δ 147.63, 144.67, 137.61, 135.98, 135.55, 128.54, 127.92, 127.66, 127.14, 126.51, 124.43, 124.38, 122.96, 73.44, 70.71, 55.84, 41.11, 24.42.

***(R)*-(6-((Benzyloxy)methyl)-3,6-dihydropyridin-1(2*H*)-yl)(4-(4-bromophenyl)-1*H*-1,2,3-triazol-1-yl)methanone (11)**. The title compound was synthesized from (*R*)-6-((benzyloxy)methyl)-1,2,3,6-tetrahydropyridine (50.0 mg, 0.246 mmol) according to the procedure described for compound **3**. This furnished compound **11** (35.7 mg, 0.079

mmol, 32% yield). $[\alpha]_D^{22}$ = 136.3 (c = 2.5, $CHCl_3$). The enantiomeric purity was determined on a Daicel Chiralcel OD-H column (4.6 X 250 mm, 20:80 IPA/Hex, flow rate of 1 mL/min): 17.4 min, e.e.>93%. HRMS calculated for $C_{22}H_{21}BrN_4O_2$ $[M+H]^+$. 453.0921, found: 453.0920. ^1H NMR (($(CD_3)_2SO$, 400 MHz, 100 °C): δ 8.82 (s, 1H), 7.85 (d, J = 6.8 Hz, 2H), 7.66 (d, J = 6.8 Hz, 2H), 7.31-7.26 (m, 5H), 6.05 - 6.01 (m, 1H), 5.80 – 5.76 (m, 1H), 4.92 (s, 1H), 4.50 (s, 2H), 4.12 (dd, J = 5.6 Hz, 13.2 Hz, 1H), 3.75-3.67 (m, 2H), 3.38 (t, J = 13.2 Hz, 1H), 2.49 – 2.40 (m, 1H), 2.18 – 2.16 (m, 1H). ^{13}C NMR ($CDCl_3$, 400MHz) δ 145.54, 137.70, 132.06, 129.60, 128.62, 128.44, 127.79, 127.71, 127.52, 127.40, 122.52, 121.39, 73.27, 70.71, 55.56, 38.87, 24.83.

(*R*)-(6-((Benzyloxy)methyl)-3,6-dihydropyridin-1(2*H*)-yl)(4-(4-phenoxyphenyl)-1*H*-1,2,3-triazol-1-yl)methanone (12). The title compound was synthesized from ((*R*)-6-((benzyloxy)methyl)-1,2,3,6-tetrahydropyridine (70.0 mg, 0.344 mmol) and 4-(4-phenoxyphenyl)-1*H*-1,2,3-triazole (90.0 mg, 0.443 mmol) according to the procedure described for compound **3**. This furnished compound **12** (52.0 mg, 0.112 mmol, 32% yield). $[\alpha]_D^{20}$ = 112.5 (c = 1.0, $CHCl_3$). HRMS calculated for $C_{28}H_{26}N_4O_3$ $[M+H]^+$. 467.2078, found: 467.2077. ^1H NMR (400 MHz, $CDCl_3$) δ 8.15 (br, 1H), 7.76 (br, 2H), 7.39 – 7.27 (m, 7H), 7.17 – 7.10 (m, 1H), 7.08 – 7.04 (m, 4H), 6.09 – 5.96 (m, 1H), 5.70 (br s, 1H), 5.31 (br, 0.5H), 4.95 (br, 0.5H), 4.48 (br, 3H), 3.90 – 3.21 (m, 3H), 2.55 (br, 1H), 2.16 (br, 1H). ^{13}C NMR (101 MHz, $CDCl_3$) δ 157.85, 156.84, 149.76, 146.23, 137.78, 129.95, 128.51, 127.83, 127.75, 127.55, 127.51, 126.15, 124.74, 123.74, 120.73, 119.30, 119.07, 73.34, 70.70, 55.57, 38.83, 24.83.

(*R*)-(6-((Benzyloxy)methyl)-3,6-dihydropyridin-1(2*H*)-yl)(3-(4-bromophenyl)-1*H*-pyrazol-1-yl)methanone (13). The title compound was synthesized from (*R*)-6-((benzyloxy)methyl)-1,2,3,6-tetrahydropyridine (63.0 mg, 0.310 mmol) and 3-(4-bromophenyl)-1H-pyrazole (76.0 mg, 0.341 mmol) according to the procedures described for compound **3**. This furnished compound **13** (119 mg, 0.263 mmol, 85% yield). $[\alpha]_D^{20}$ = 93.8 (c = 1.0, $CHCl_3$). HRMS calculated for $C_{23}H_{22}BrN_3O_2$ $[M+H]^+$. 452.0968, found: 452.0969. ^1H NMR (400 MHz, $CDCl_3$) δ 8.12 (d, J = 2.8 Hz, 1H), 7.68 (d, J = 8.5 Hz, 2H), 7.50 (d, J = 8.4 Hz, 2H), 7.36 – 7.20 (m, 5H), 6.61 (d, J = 2.4 Hz, 1H), 6.05 – 5.96 (m, 1H), 5.82 – 5.74 (m, 1H), 5.35 (br, 1H), 4.55 (br, 3H), 3.85 (br, 1H), 3.79 – 3.75 (m, 1H), 3.32 (br, 1H), 2.55 (br, 1H), 2.10 (dt, J = 17.4, 4.1 Hz, 1H). ^{13}C NMR (101 MHz, $CDCl_3$) δ 152.18, 138.12, 133.54, 131.88, 131.46, 128.40, 127.67, 127.62, 127.52, 127.10, 125.48, 122.66, 114.16, 104.49, 73.30, 71.24, 54.98, 41.93, 25.14.

(*R*)-(6-((Benzyloxy)methyl)-3,6-dihydropyridin-1(2*H*)-yl)(4-(4-bromophenyl)-1*H*-imidazol-1-yl)methanone (14). The title compound was synthesized from (*R*)-6-((benzyloxy)methyl)-1,2,3,6-tetrahydropyridine (68.0 mg, 0.335 mmol) and 4-(4-bromophenyl)-1*H*-imidazole (82.0 mg, 0.368 mmol) according to the procedure described for compound **3**. This furnished compound **14** (129 mg, 0.284 mmol, 85% yield). $[\alpha]_D^{20}$ = 80.8 (c = 1.0, $CHCl_3$). HRMS calculated for $C_{23}H_{22}BrN_3O_2$ $[M+H]^+$. 452.0968, found: 452.0965. ^1H NMR (400 MHz, $CDCl_3$) δ 8.03 (s, 1H), 7.67 (s, 1H), 7.54 (d, J = 8.3 Hz, 2H), 7.45 (d, J = 8.4 Hz, 2H), 7.37 – 7.27 (m, 5H), 5.98 – 5.95 (m, 1H), 5.59 (d, J = 8.4 Hz, 1H), 4.66 (br, 1H), 4.54 (s, 2H), 4.17 – 4.05 (m, 1H), 3.67 – 3.61 (m, 2H), 3.29 – 3.19 (m, 1H), 2.53 – 2.36 (m, 1H), 2.10 (dt, J = 16.0, 4.0 Hz, 1H). ^{13}C NMR (101 MHz, $CDCl_3$) δ 151.50, 140.97, 137.57, 137.38, 132.18, 131.67, 128.59,

128.06, 127.92, 126.70, 126.66, 123.97, 121.01, 114.11, 73.48, 69.66, 55.31, 38.52, 24.66.

(*R*)-(3-([1,1'-Biphenyl]-4-yl)-1*H*-pyrazol-1-yl)(6-((benzyloxy)methyl)-3,6-dihydropyridin-1(2*H*)-yl)methanone (15). A solution of (*R*)-(6-((benzyloxy)methyl)-3,6-dihydropyridin-1(2*H*)-yl)(3-(4-bromophenyl)-1*H*-pyrazol-1-yl)methanone **13** (40.0 mg, 0.088 mmol) in dioxane and water (2:1; 6 mL) was treated with phenylboronic acid (21.6 mg, 0.177 mmol), K_2CO_3 (36.7 mg, 0.265 mmol), $PdCl_2$(dppf) (9.71 mg, 0.013 mmol) and the reaction mixture was stirred for 6h at 80 ^oC under Ar. The mixture was poured into water and extracted with ethyl acetate (3 x 20 mL), the organic layer was washed with water and brine, dried over $MgSO_4$ and concentrated under reduced pressure. The residue was purified by flash chromatography to furnish compound **15** (30.6 mg, 0.068 mmol, 77% yield). $[\alpha]_D^{20}$ = 52.9 (*c* = 1.0, $CHCl_3$). HRMS calculated for $C_{29}H_{27}N_3O_2$ $[M+H]^+$. 450.2176, found: 450.2173. ^1H NMR (400 MHz, $CDCl_3$) δ 8.15 (d, *J* = 2.7 Hz, 1H), 7.91 (d, *J* = 8.3 Hz, 2H), 7.65 – 7.62 (m, 4H), 7.49 – 7.43 (m, 2H), 7.39 – 7.25 (m, 6H), 6.70 (d, *J* = 2.4 Hz, 1H), 6.08 – 5.98 (m, 1H), 5.81 (d, *J* = 8.2 Hz, 1H), 5.42 (br, 1H), 4.58 (s, 3H), 3.89 (br, 1H), 3.82 (dd, *J* = 8.0, 4.0 Hz, 1H), 3.35 (br s, 1H), 2.65 – 2.53 (m, 1H), 2.13 (dt, *J* = 17.4, 4.1 Hz, 1H). ^{13}C NMR (101 MHz, $CDCl_3$) δ 153.01, 141.46, 140.72, 138.27, 134.16, 133.47, 131.55, 128.97, 128.49, 127.72, 127.62, 127.53, 127.24, 127.13, 126.78, 126.58, 125.67, 104.73, 73.38, 71.73, 54.41, 41.64, 25.18.

***(R)*-(3-([1,1'-Biphenyl]-4-yl)-1*H*-pyrazol-1-yl)(6-((benzyloxy)methyl)-3,6-dihydropyridin-1(2*H*)-yl)methanone (16)**. The title compound was synthesized from compound **14** (40.0 mg, 0.088 mmol) according to the procedure described for compound **15**. This furnished compound **16** (27.8 mg, 0.062 mmol, 70% yield). $[\alpha]_D^{20}$ = 82.0 (*c* = 1.0, $CHCl_3$). HRMS calculated for $C_{29}H_{27}N_3O_2$ $[M+H]^+$. 450.2176, found: 450.2166. ^1H NMR (400 MHz, $CDCl_3$) δ 8.06 (s, 1H), 7.78 (d, *J* = 8.1 Hz, 2H), 7.70 (s, 1H), 7.68 – 7.60 (m, 4H), 7.46 – 7.42 (m, 2H), 7.39 – 7.31 (m, 6H), 6.01 – 5.97 (m, 1H), 5.61 (d, *J* = 8.0 Hz, 1H), 4.71 (br, 1H), 4.57 (d, *J* = 12.0 Hz, 2H), 4.18 – 4.03 (m, 1H), 3.69 – 3.60 (m, 2H), 3.28 (br, 1H), 2.46 (br, 1H), 2.11 (dt, *J* = 17.5, 4.0 Hz, 1H). ^{13}C NMR (101 MHz, $CDCl_3$) δ 151.76, 141.86, 140.88, 140.13, 137.62, 137.54, 132.27, 128.88, 128.70, 128.14, 128.00, 127.88, 127.41, 127.35, 127.03, 125.60, 124.21, 113.91, 73.58, 69.82, 55.37, 39.91, 24.85.

(*R*)-(3-([1,1'-Biphenyl]-4-yl)-1*H*-1,2,4-triazol-1-yl)(6-((benzyloxy)methyl)-3,6-dihydropyridin-1(2*H*)-yl)methanone (17). A solution of (*R*)-6-((benzyloxy)methyl)-1,2,3,6-tetrahydropyridine (60.0 mg, 0.295 mmol) in THF was treated with DIPEA (0.155 mL, 0.885 mmol) and bis(trichloromethyl) carbonate (43.8 mg, 0.148 mmol) and the reaction mixture was stirred for 30 min at 0 ^oC. After that the reaction mixture was poured into water and extracted with ethyl acetate (3 x 10 mL).The organic layer was washed with water, brine and dried over $MgSO_4$, filtered, and concentrated under reduced pressure. The intermediate was dissolved in THF and DIPEA (0.155 mL, 0.885 mmol), DMAP (36.1 mg, 0.295 mmol) and 3-bromo-1*H*-1,2,4-triazole (48.0 mg, 0.325 mmol) were added to the solution. The mixture was stirred for 2h at 60 ^oC and poured into saturated aqueous NH_4Cl solution (20 mL). The mixture was extracted with ethyl acetate (3 x 20 mL), washed with water, brine, dried over $MgSO_4$ and filtered. The solvents are

removed under reduced pressure to yield the crude 1,2,4-triazole urea, which was purified by silica gel chromatography (1-10% ethyl acetate/pentane). The purified 1,2,4-triazole urea (40.0 mg, 0.106 mmol) was subsequently reacted with [1,1'-biphenyl]-4-ylboronic acid (46.2 mg, 0.233 mmol) according to the same procedure described for compound **15**. This furnished compound **17** (35.8 mg, 0.080 mmol, 30% yield overall). $[\alpha]_D^{22}$ = 109.3 (*c* = 0.6, $CHCl_3$). HRMS calculated for $C_{28}H_{26}N_4O_2$ $[M+H]^+$. 451.2129, found: 451.2128. ^1H NMR (400 MHz, $CDCl_3$) δ 8.76 (s, 1H), 8.20 (d, *J* = 8.1 Hz, 2H), 7.69 (d, *J* = 8.2 Hz, 2H), 7.65 (d, *J* = 7.5 Hz, 2H), 7.50 – 7.43 (m, 2H), 7.39 – 7.25 (m, 6H), 6.08 – 6.01 (m, 1H), 5.76 (d, *J* = 8.1 Hz, 1H), 5.48 (br, 1H), 4.54 (br, 3H), 3.81 – 3.69 (m, 2H), 3.34 (br, 1H), 2.55 (br, 1H), 2.15 (dt, *J* = 16.0, 4.0 Hz, 1H). ^{13}C NMR (101 MHz, $CDCl_3$) δ 162.33, 147.48, 142.84, 140.56, 137.96, 129.06, 128.99, 128.94, 128.55, 127.88, 127.81, 127.67, 127.50, 127.36, 127.21, 124.84, 114.20, 73.41, 71.32, 55.34, 42.71, 25.68.

4-Nitrophenyl (*R*)-6-((benzyloxy)methyl)-3,6-dihydropyridine-1(2*H*)-carboxylate (18). To a stirred solution of 4-nitrophenol (38.3 mg, 0.275 mmol), pyridine (0.032 mL, 0.394 mmol) in dichloromethane, triphosgene (29.2 mg, 0.098 mmol) was added. After stirring at room temperature for 1h, TLC was used to confirm that reaction was complete. (*R*)-6-((benzyloxy)methyl)-1,2,3,6-tetrahydropyridine (40.0 mg, 0.197 mmol) and pyridine were then added to the mixture, and the reaction mixture was stirred for 12h at room temperature. Dichloromethane was removed *in vacuo* and the residual was extracted with ethyl acetate (3 x 20 mL). The organic layer was washed with water, brine, and dried over $MgSO_4$. The crude product was purified by column chromatography (2-20% ethyl acetate/pentane) to afford compound **18** (56.5 mg, 0.153 mmol, 78% yield). $[\alpha]_D^{20}$ = 87.2 (*c* = 1.0, $CHCl_3$). HRMS [ESI+] m/z: calculated for $C_{20}H_{20}N_2O_5$ $[M+H]^+$. 369.3914, found: 369.3915. ^1H NMR (400 MHz, $CDCl_3$) δ 8.24 (d, *J* = 8.0 Hz, 1H), 8.14 (d, *J* = 12 Hz, 1H), 7.37 – 7.27 (m, 6H), 7.12 (d, *J* = 8.8 Hz, 1H), 6.02 (br, 1H), 5.74 (t, *J* = 12.7 Hz, 1H), 4.77 (br, 1H), 4.64 – 4.52 (m, 2H), 4.27 (dd, *J* = 13.3, 5.9 Hz, 1H), 3.71 – 3.58 (m, 2H), 3.31 (t, *J* = 11.0 Hz, 0.4H), 3.10 (td, *J* = 12.7, 3.4 Hz, 0.6H), 2.39 – 2.30 (m, 1H), 2.10 (d, *J* = 17.3 Hz, 1H). ^{13}C NMR (101 MHz, $CDCl_3$) δ 156.50, 152.99, 144.84, 137.90, 128.60, 128.00, 127.76, 125.50, 125.09, 124.41, 122.42, 73.49, 70.93, 52.97, 37.91, 24.85.

***tert*-Butyl (*R*)-6-(((*tert*-butyldiphenylsilyl)oxy)methyl)-3,6-dihydropyridine-1(2H)-carboxylate (19).** Compound **19** was prepared according to the reported method [1].

***tert*-Butyl (*R*)-2-(((*tert*-butyldiphenylsilyl)oxy)methyl)piperidine-1-carboxylate (20)**. Compound **19** was prepared according to the reported method [1]. Obtained *tert*-Butyl (*R*)-6-(((*tert*-butyldiphenylsilyl)oxy)methyl)-3,6-dihydropyridine-1(2*H*)-carboxylate **19** (800 mg, 1.77 mmol) was dissolved in MeOH (40 mL) and Pd/C (188 mg, 0.177 mmol) were added subsequently. The reaction was stirred overnight under a hydrogen atmosphere. After filtering over Celite and evaporation of the solvents the crude target compound was obtained. The residue was purified by flash chromatography (pentane/EtOAc = 99 : 1 → 90 : 10) to furnish the title compound (763 mg, 1.68 mmol, 95% yield) as a colorless oil ^1H NMR (400 MHz, $CDCl_3$) δ 7.67 (d, *J* = 7.0 Hz, 4H), 7.43 – 7.37 (m, 6H), 4.36 (br, 1H), 3.95 (d, *J* = 11.2 Hz, 1H), 3.72- 3.65 (m, 2H), 2.63 (t, *J* = 12.1 Hz, 1H), 1.92 (d, *J* = 12.0 Hz, 1H), 1.55 (d, *J* = 8.0 Hz, 3H), 1.43 (app. s, 11H), 1.05 (s, 9H). ^{13}C NMR (101 MHz, $CDCl_3$) δ 155.08, 135.56, 133.54, 129.66, 127.70, 79.10,

61.55, 51.84, 40.10, 28.47, 26.84, 25.31, 25.01, 19.20. LC-MS m/z: calculated for $C_{27}H_{39}NO_3Si$ $[M+H]^+$ 454.27, found: 454.06.

***tert*-Butyl (*R*)-2-(hydroxymethyl)piperidine-1-carboxylate (21)**. A solution of TBAF (3.17 mL, 3.17 mmol) was added to a solution of *tert*-butyl (*R*)-2-(((*tert*-butyldiphenylsilyl)oxy)methyl)piperidine-1-carboxylate **20** (960 mg, 2.12 mmol) in THF (30 mL) with ice cooling and the mixture was stirred at r.t. for 18h. After being diluted with water, the mixture was extracted with ethyl acetate (3 x 30 mL), the organic layer was washed with water and brine, dried over $MgSO_4$, filtered and concentrated under reduced pressure. The residue was purified by flash chromatography (pentane/EtOAc = 10 : 1 → 3 : 1) to furnish the title compound (446 mg, 2.07 mmol, 98% yield) as a colorless oil. ^1H NMR (400 MHz, $CDCl_3$) δ 4.33 – 4.17 (m, 1H), 3.90 (d, *J* = 12.2 Hz, 1H), 3.76 – 3.71 (m, 1H), 3.57 (dd, *J* = 11.0, 6.4 Hz, 1H), 2.81 (t, *J* = 12.2 Hz, 1H), 2.70 (br, 1H), 1.67 (d, *J* = 11.2 Hz, 1H), 1.62 – 1.50 (m, 3H), 1.41 (app. s, 11H). ^{13}C NMR (101MHz, $CDCl_3$) δ 155.23, 79.81, 61.25, 52.40, 39.93, 28.34, 25.31, 25.15, 19.56. LC-MS m/z: calculated for $C_{11}H_{21}NO_3$ $[M+H]^+$ 216.15, found: 216.52.

***tert*-Butyl (*R*)-2-((benzyloxy)methyl)piperidine-1-carboxylate (22)**. To a solution of **21** (217 mg, 1.01 mmol), BnBr (345 mg, 2.02 mmol), TBAI (14.9 mg, 0.040 mmol) in dry DMF 5 mL, was added NaH (81.0 mg, 2.02 mmol, 60% in mineral oil) with ice cooling. The reaction mixture was stirred overnight and quenched with saturated aqueous ammonium chloride. The mixture was diluted with DCM and washed with water, brine, dried over $MgSO_4$, filtered and concentrated under reduced pressure. The residue was purified by flash chromatography (pentane/EtOAc = 10 : 1 → 3 : 1) to furnish the title compound (277 mg, 0.907 mmol, 90% yield) as a colorless oil. ^1H NMR (400 MHz, $CDCl_3$) δ 7.36 – 7.29 (m, 4H), 7.28 – 7.25 (m, 1H), 4.53 (d, *J* = 12.0 Hz, 2H), 4.44 (br, 1H), 3.97 (d, *J* = 12.9 Hz, 1H), 3.53 (d, *J* = 7.3 Hz, 2H), 2.72 (t, *J* = 12.6 Hz, 1H), 1.86 (d, *J* = 12.0 Hz, 1H), 1.67 – 1.49 (m, 3H), 1.44 (app. s, 11H). ^{13}C NMR (101 MHz, $CDCl_3$) δ 155.22, 138.44, 128.32, 127.51, 79.25, 72.77, 67.87, 49.29, 40.01, 28.45, 25.32, 25.22, 19.24. LC-MS m/z: calculated for $C_{18}H_{27}NO_3$ $[M+H]^+$ 306.20, found: 306.01.

(*R*)-2-((Benzyloxy)methyl)piperidine (23). Compound **22** (264 mg, 0.864 mmol) was dissolved in a mixture of 25% TFA in DCM (5 mL). The reaction mixture was stirred at r.t. for 2.5h until TLC analysis showed the reaction was completely converted. The mixture was co-evaporated with toluene (3 x 20 mL), the residue diluted with ethyl acetate and washed with 10% Na_2CO_3, water, brine and dried over $MgSO_4$, filtered and concentrated under reduced pressure to afford the crude product that was used without further purification (151 mg, 0.735 mmol, 85% yield) as a colorless oil. ^1H NMR (400 MHz, $CDCl_3$) δ 7.35 – 7.30 (m, 4H), 7.28 – 7.23 (m, 1H), 4.50 (d, *J* = 12.0 Hz, 2H), 3.44 (dd, *J* = 9.0, 3.6 Hz, 1H), 3.31 (t, *J* = 8.8 Hz, 1H), 3.06 (d, *J* = 11.6 Hz, 1H), 3.00 (br, 1H),

2.80 – 2.74 (m, 1H), 2.61 (td, J = 11.7, 2.8 Hz, 1H), 1.78 (d, J = 11.8 Hz, 1H), 1.59 (d, J = 13.1 Hz, 1H), 1.52 (d, J = 13.0 Hz, 1H), 1.49 – 1.26 (m, 2H), 1.21 – 1.03 (m, 1H). ^{13}C NMR (101 MHz, $CDCl_3$) δ 138.27, 128.36, 127.73, 127.60, 75.16, 73.37, 56.27, 46.41, 28.63, 26.18, 24.37. LC-MS m/z: calculated for $C_{13}H_{19}NO$ $[M+H]^+$ 206.15, found: 206.43.

***tert*-Butyl (*R*)-6-(hydroxymethyl)-3,6-dihydropyridine-1(2*H*)-carboxylate (24)**. The title compound was synthesized from *tert*-butyl (*R*)-6-(((*tert*-butyldiphenylsilyl)oxy)methyl)-3,6-dihydropyridine-1(2*H*)-carboxylate **19** (596 mg, 1.32 mmol) and TBAF (1.59 mL, 1.59 mmol) according to the procedures described for compound **21**. This furnished title compound (196 mg, 0.920 mmol, 92% yield) as a yellow oil. ^{1}H NMR ($CDCl_3$, 400 MHz,) δ 5.86 (br, 1H), 5.58 (dt, J = 10.2, 2.8 Hz, 1H), 4.43 (br, 1H), 3.99 (br, 1H), 3.57 (d, J = 6.6 Hz, 2H), 3.29 (br, 1H), 2.86 (br, 1H), 2.10 (br, 1H), 1.88 (dt, J = 17.2, 4.2 Hz, 1H), 1.38 (s, 9H). ^{13}C NMR ($CDCl_3$, 101 MHz,) δ 154.48, 127.35, 124.97, 79.97, 64.71, 54.01, 38.22, 28.37, 24.80. LC-MS m/z: calculated for $C_{11}H_{19}NO_3$ $[M+H]^+$ 214.28, found: 214.72.

***tert*-Butyl (*R*)-6-((benzyloxy)methyl)-3,6-dihydropyridine-1(2*H*)-carboxylate (25)**. The title compound was synthesized from compound **24** (520 mg, 2.44 mmol) according to the procedures described for the preparation of compound **22**. This furnished title compound (666 mg, 2.19 mmol, 90% yield) as a yellow oil. ^{1}H NMR ($CDCl_3$, 400 MHz): δ 7.35 – 7.23 (m, 5H), 5.88 – 5.84 (m, 1H), 5.58 (dt, J =12.0, 4.0 Hz, 1H), 4.52 (s, 2H), 4.28 (br, 1H), 4.05 (br, 1H), 3.56 (br, 1H), 3.51 (br, 1H), 2.93 (br, 1H), 2.29 (br, 1H), 1.97 (d, J = 8.7 Hz, 1H), 1.38 (s, 9H). ^{13}C NMR ($CDCl_3$, 101MHz) δ 154.53, 138.32, 128.31, 128.25, 127.66, 127.53, 127.37, 79.40, 72.03, 71.26, 51.90, 37.06, 28.40, 25.00. LC-MS m/z: calculated for $C_{18}H_{25}NO_3$ $[M+H]^+$ 304.40, found: 303.99.

(*R*)-6-((Benzyloxy)methyl)-1,2,3,6-tetrahydropyridine (26). The title compound was synthesized from compound **25** (303 mg, 0.999 mmol) according to the procedures described for the preparation of compound **23**. This furnished the title compound (181 mg, 0.890 mmol, 89% yield) as a colorless oil. ^{1}H NMR (400 MHz, $CDCl_3$) δ 7.37 – 7.30 (m, 4H), 7.29 – 7.24 (m, 1H), 5.86 – 5.83 (m, 1H), 5.53 (dt, J = 10.2, 1.9 Hz, 1H), 4.54 (d, J = 12.0 Hz, 2H), 3.62 – 3.54 (m, 1H), 3.56 – 3.46 (m, 1H), 3.46 – 3.37 (m, 1H), 3.09 – 3.03 (m, 1H), 2.87 – 2.81 (m, 1H), 2.24 – 2.13 (m, 2H), 2.04 – 1.97 (m, 1H). ^{13}C NMR (101 MHz, $CDCl_3$) δ 138.26, 128.43, 127.78, 127.68, 126.81, 73.61, 73.38, 53.82, 41.32, 25.98. LC-MS m/z: calculated for $C_{13}H_{17}NO$ $[M+H]^+$ 204.29, found: 204.68.

(*R*)-(6-((Benzyloxy)methyl)-3,6-dihydropyridin-1(2*H*)-yl)(3-bromo-1*H*-1,2,4-triazol-1-yl)methanone (30). A solution of (*R*)-6-((benzyloxy)methyl)-1,2,3,6-tetrahydropyridine **26** (60.0 mg, 0.295 mmol) in THF was treated with DIPEA (0.155 mL,

0.885 mmol) and bis(trichloromethyl) carbonate (43.8 mg, 0.148 mmol) and the reaction mixture was stirred for 30 min at 0 ^oC. After that the reaction mixture was poured into water and extracted with ethyl acetate (3 x 10 mL). The organic layer was washed with water and brine, dried over $MgSO_4$, filtered, and concentrated under reduced pressure. The intermediate was dissolved in THF and DIPEA (0.155 mL, 0.885 mmol), DMAP (36.1 mg, 0.295 mmol) and 4-(4-bromophenyl)-1*H*-1,2,3-triazole (48.0 mg, 0.325 mmol) were added to the solution. The mixture was stirred for 2h at 60 ^oC and poured into saturated aqueous NH_4Cl solution (20 mL). The mixture was extracted with ethyl acetate (3 x 20 mL), washed with water, brine, dried over $MgSO_4$ and filtered. The solvents were removed under reduced pressure to yield the crude product. Purification by silica gel chromatography (pentane/EtOAc 100:1 → 5:1) afforded title compound (45.0 mg, 0.119 mmol, 40% yield). ^1H NMR (400 MHz, $CDCl_3$) δ 8.49 (s, 1H), 7.54 – 7.02 (m, 5H), 6.01 (dd, *J* = 10.3, 6.0 Hz, 1H), 5.69 (s, 1H), 5.51 – 4.79 (m, 1H), 4.70 – 4.21 (m, 3H), 3.65 (s, 2H), 3.26 (s, 1H), 2.43 (d, *J* = 14.4 Hz, 1H), 2.22 – 2.01 (m, 1H). ^{13}C NMR (101 MHz, $CDCl_3$) δ 155.54, 147.91, 141.23, 137.74, 128.56, 127.94, 127.69, 127.42, 124.86, 73.32, 70.52, 54.66, 29.80, 25.04. LC-MS m/z: calculated for $C_{16}H_{17}BrN_4O_2$ $[M+H]^+$ 378.24, found: 378.59.

***tert*-butyl (*3R,6R*)-6-((benzyloxy)methyl)-3-((*tert*-butyldiphenylsilyl)oxy)-3,6-dihydropyridine-1(2*H*)-carboxylate (27).** Compound **27** was prepared according the route reported [2,3].

(*3R,6R*)-6-((Benzyloxy)methyl)-3-((*tert*-butyldiphenylsilyl)oxy)-1,2,3,6-tetrahydropyridine (28). The Boc-protected compound *tert*-butyl (*3R,6R*)-6-((benzyloxy)methyl)-3-((*tert*-butyldiphenylsilyl)oxy)-3,6-dihydropyridine-1(2*H*)-carboxylate **27** was prepared according the route reported [2,3]. The title compound was synthesized from **27** (500 mg, 0.896 mmol) according to the procedures described for compound **23** and furnished the free amine **28** (347 mg, 0.758 mmol, 85% yield) as a light yellow oil. ^1H NMR (400 MHz, $CDCl_3$) δ 7.70 – 7.65 (m, 4H), 7.39 – 7.22 (m, 11H), 5.71 (s, 2H), 4.54 (s, 2H), 4.0 – 4.06 (m, 1H), 3.55 – 3.51 (m, 1H), 3.49 – 3.45 (m, 1H), 3.39 – 3.35(m, 1H), 2.98 (dd, *J* = 13.8, 4.2Hz, 1H), 2.77 (dd, *J* = 12.2, 4.0 Hz, 1H), 2.56 (br s, 1H), 1.07 (s, 9H). ^{13}C NMR (101 MHz, $CDCl_3$) δ 138.19, 135.80, 135.69, 134.15, 134.01, 130.67, 130.39, 129.65, 129.58, 128.33, 127.62, 127.54, 127.52, 127.51, 73.18, 71.82, 64.24, 53.71, 48.88, 26.96, 19.15.

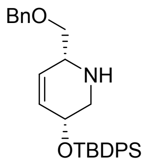

(*3R,6S*)-6-((Benzyloxy)methyl)-3-((*tert*-butyldiphenylsilyl)oxy)-1,2,3,6-tetrahydropyridine (31). The Boc-protected *tert*-butyl (*3R,6S*)-6-((benzyloxy)methyl)-3-((*tert*-butyldiphenylsilyl)oxy)-3,6-dihydropyridine-1(2*H*)-carboxylate was prepared according the route reported [2,3]. The target compound was

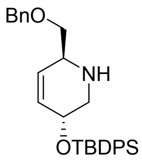

synthesized from Boc-protected compound (500 mg, 0.896 mmol) according to the procedures described for compound **23**. This furnished the title compound (357 mg, 0.781 mmol, 87% yield) as light yellow oil. ^{1}H NMR (400 MHz, $CDCl_3$) δ 7.75 (br s, 1H), 7.69 – 7.63 (m, 4H), 7.43 – 7.34 (m, 6H), 7.32 – 7.23(m, 5H), 5.77 (d, *J* = 12.2 Hz, 1H), 5.57 (d, *J* = 11.8 Hz, 1H), 4.47 (s, 2H), 4.33 (s, 1H), 3.67 – 3.65(m, 1H), 3.50 (dd, *J* = 8.1, 3.7 Hz, 1H), 3.37 – 3.33 (m, 1H), 3.18 (dd, *J* = 12.3, 4.1 Hz, 1H), 2.81 – 2.79 (m, 1H), 1.06 (s, 9H). ^{13}C NMR (101 MHz, $CDCl_3$) δ 137.84, 135.93, 135.86, 135.80, 133.89, 133.77, 131.95, 129.88, 129.83, 128.46, 127.82, 127.79, 127.74, 127.48, 73.41, 71.89, 65.16, 53.34, 48.61, 27.01, 19.23.

(*3S,6R*)-6-((Benzyloxy)methyl)-3-((*tert*-butyldimethylsilyl)oxy)-1,2,3,6-tetrahydropyridine (32). The Boc-protected *tert*-butyl (*3S,6R*)-6-((benzyloxy)methyl)-3-((*tert*-butyldimethylsilyl)oxy)-3,6-dihydropyridine-1(2*H*)-carboxylate was prepared according the reported route [2,3]. To a solution of Boc-protected compound (210 mg, 0.480 mmol) was added 10% TFA in DCM (5 mL) with ice cooling, the reaction mixture was stirred at r.t. for 0.5h and subsequently co-evaporated with toluene (3x20 mL), the residue was diluted with ethyl acetate, washed with 10% Na_2CO_3, water, brine and dried over $MgSO_4$, and concentrated under reduced pressure, which afforded the crude product (97.0 mg, 0.291 mmol, 54% yield) as light yellow oil. ^{1}H NMR (400 MHz, $CDCl_3$) δ 7.37 – 7.31 (m, 4H), 7.30 – 7.25 (m, 1H), 5.82 – 5.68 (m, 1H), 5.60 (dt, *J* = 10.3, 1.8 Hz, 1H), 4.60 – 4.45 (m, 2H), 4.35 – 4.29 (m, 1H), 3.62 – 3.54 (m, 1H), 3.50 (dd, *J* = 8.9, 4.0 Hz, 1H), 3.37 – 3.28 (m, 1H), 3.22 (dd, *J* = 12.1, 5.7 Hz, 1H), 2.64 (dd, *J* = 11.5, 8.5 Hz, 1H), 2.25 (br, 1H), 0.90 (s, 9H), 0.09 (s, 3H), 0.08 (s, 3H). ^{13}C NMR (101 MHz, $CDCl_3$) δ 138.14, 132.52, 128.49, 128.28, 127.84, 127.78, 73.83, 73.51, 65.84, 54.06, 50.44, 25.98, 18.31, -4.51, -4.62.

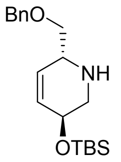

***tert*-Butyl (2*R*,3*R*)-3-hydroxy-2-(hydroxymethyl)-3,6-dihydropyridine-1(2*H*)-carboxylate (34)**. Compound **33** was prepared according to the reported methods[4]. To a solution of compound **33** (790 mg, 2.92 mmol) in MeOH (25 mL) was added catalytic amount of *p*-TsOH (27.8 mg, 0.146 mmol). The reaction mixture was stirred at r.t. for 2.5h until TLC showed the reaction was completed. The reaction mixture was evaporated under reduced pressure and the residue was purified by flash chromatography to furnish the title compound (576 mg, 2.51 mmol, 86% yield) as light yellow oil. ^{1}H NMR (400 MHz, $CDCl_3$) δ 5.77 (br, *J* = 12.0 Hz, 1H), 5.69 (d, *J* = 12.0 Hz, 1H), 4.68 – 4.57 (m, 2H), 4.16 – 4.04 (m, 1H), 4.00 – 3.98 (m, 1H), 3.61 (dd, *J* = 11.3, 6.7 Hz, 1H), 3.51 (d, *J* = 16.5 Hz, 1H), 3.02 (br, 2H), 1.47 (s, 9H). ^{13}C NMR (101 MHz, $CDCl_3$) δ 155.03, 128.19, 124.16, 80.79, 66.91, 60.43, 53.37, 40.94, 28.50. LC-MS m/z: calculated for $C_{11}H_{19}NO_4$ $[M+H]^+$ 230.28, found: 230.86.

***tert*-Butyl (*2R,3R*)-2-((benzyloxy)methyl)-3-hydroxy-3,6-dihydropyridine-1(2*H*)-carboxylate (35)**. 2-Aminoethylborinate (10 mol%), *tert*-butyl (*2R,3R*)-3-hydroxy-2-(hydroxymethyl)-3,6-dihydropyridine-1(2*H*)-carboxylate **34** (250 mg, 1.09 mmol), KI (217 mg, 1.31 mmol) and K_2CO_3 (181 mg, 1.31 mmol) were transferred to a 2-dram vial containing a magnetic stir bar. The vial was then sealed with a septum and purged with argon. Anhydrous acetonitrile was added to the flask, followed by benzyl bromide (0.233 mL, 1.96 mmol). The resulting mixture was stirred at 60 ^oC for 24h. The mixture was then transferred to a separation funnel containing water and ethyl acetate, the organic layer was separated, and the aqueous layer was extracted two more times with ethyl acetate. The combined organic layers were washed with brine, dried over $MgSO_4$, filtered, and concentrated in *vacuo*. The resulting crude material was purified by silica gel chromatography (pentane/EtOAc 10:1 → 2:1) to furnish the title compound (310 mg, 0.972 mmol, 89% yield) as colorless oil. ^{1}H NMR (400 MHz, $CDCl_3$) δ 7.37 – 7.19 (m, 5H), 5.73 (d, *J* = 10.4 Hz, 1H), 5.65 (br, 1H), 4.91 (br, 1H), 4.59 – 4.49 (m, 1H), 4.44 (d, *J* = 11.9 Hz, 1H), 4.08 (br, 1H), 3.85 – 3.73 (m, 1H), 3.72 – 3.33 (m, 3H), 1.46 (s, 9H). ^{13}C NMR (101 MHz, $CDCl_3$) δ 155.11, 137.89, 128.68, 128.41, 127.73, 127.63, 123.92, 80.32, 73.08, 66.21, 65.93, 50.01, 40.73, 28.46. LC-MS m/z: calculated for $C_{18}H_{25}NO_4$ $[M+H]^+$ 320.40, found: 320.76.

***tert*-Butyl (*2R,3R*)-2-((benzyloxy)methyl)-3-((*tert*-butyldimethylsilyl)oxy)-3,6-dihydropyridine-1(2*H*)-carboxylate (36).** Imidazole (243 mg, 3.57 mmol) and TBS-Cl (484 mg, 3.21 mmol) were added to a stirred solution of compound **35** (570 mg, 1.79 mmol) in DMF with ice cooling, and stirred at room temperature for 2h. The reaction mixture was quenched with water, and extracted with EtOAc (3 x 50 mL). The organic layer was washed with water and brine, dried over $MgSO_4$, filtered, and concentrated *in vacuo*. The resulting crude material was purified by silica gel chromatography (pentane/EtOAc 100:1 →10:1) to furnish the title compound (735 mg, 1.70 mmol, 95% yield) as colorless oil. ^{1}H NMR (400 MHz, $CDCl_3$) δ 7.39 – 7.28 (m, 5H), 5.70 – 5.57 (m, 2H), 5.03 – 4.84 (m, 1H), 4.79 – 4.41 (m, 3H), 4.32 – 4.22 (m, 0.5H), 4.12 – 4.03 (m, 0.5H), 3.77 – 3.70 (m, 1H), 3.64 – 3.52 (m, 1H), 3.53 – 3.41 (m, 1H), 1.53 (s, 9H), 0.94 (s, 9H), 0.15 (s, 6H). ^{13}C NMR (101 MHz, $CDCl_3$) δ 155.34, 138.63, 129.08, 128.34, 127.73, 127.52, 124.54, 79.86, 72.60, 66.18, 65.12, 53.81, 40.48, 28.47, 25.85, 18.11, -4.69, -4.84. LC-MS m/z: calculated for $C_{24}H_{39}NO_4Si$ $[M+H]^+$ 434.66, found: 434.12.

(*2R,3R*)-2-((Benzyloxy)methyl)-3-((*tert*-butyldimethylsilyl)oxy)-1,2,3,6-tetrahydropyridine (37). To a solution of compound **36** (540 mg, 1.25 mmol) was added 10% TFA in DCM (5mL) in DCM with ice cooling. The reaction mixture was stirred at r.t. for 0.5h. The reaction mixture was co-evaporated with toluene (3 x 20 mL), the residue was diluted with ethyl acetate and washed with 10% Na_2CO_3, water, brine and dried over $MgSO_4$. Concentration under reduced pressure afforded the crude product (290

mg, 0.871 mmol, 69% yield). ^{1}H NMR (400 MHz, $CDCl_3$): δ 7.40 – 7.34 (m, 4H), 7.30 – 7.28 (m, 1H), 5.88 – 5.79 (m, 2H), 4.53 (s, 2H), 4.14 – 4.07 (m, 1H), 3.54 – 3.51 (m, 2H), 3.38 – 3.34 (m, 1H), 3.32 (d, *J* = 4.0 Hz, 1H), 2.99 (dt, *J* = 4.0 Hz, 8.0 Hz, 1H), 2.94 – 2.87 (m, 1H), 0.87 (s, 9H), 0.06 (s, 3H), 0.05 (s, 3H). ^{13}C NMR (101 MHz, $CDCl_3$) δ 138.39, 130.42, 128.48, 128.18, 128.01, 127.74, 73.58, 70.37, 63.46, 57.38, 44.90, 26.01, 18.21, -3.89, -4.72. LC-MS m/z: calculated for $C_{19}H_{31}NO_2Si$ $[M+H]^+$ 334.55, found: 334.92.

S.Scheme 1. Synthesis of intermediates **42** and **47**

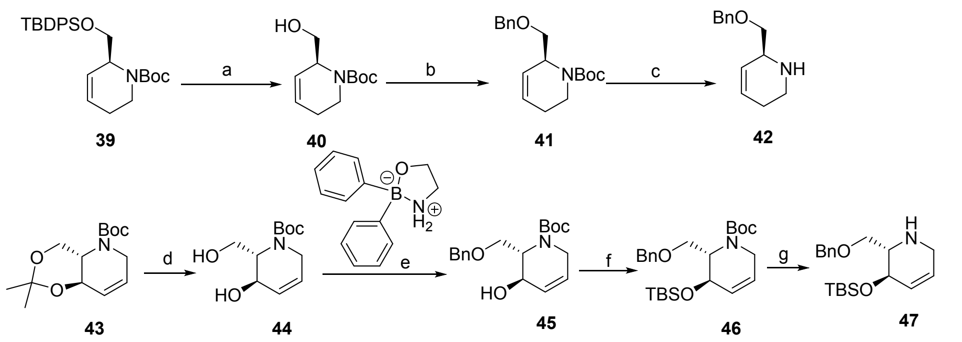

Reagents and conditions: (a) TBAF, THF, r.t. 92%; (b) BnBr, TBAI, NaH, DMF, 87%; (c) 25% TFA (v/v), DCM, r.t., 82%; (d) cat. *p*-TsOH, MeOH, 84%; (e) BnBr, KI, MeCN, 60 °C, 90%; (f) TBSCl, imidazole, DMF, 95%; (g) 10% TFA, DCM, 0 °C, 71%.

***tert*-Butyl (*S*)-6-(hydroxymethyl)-3,6-dihydropyridine-1(2*H*)-carboxylate (40).** The title compound was synthesized from compound **39** (596 mg, 1.32 mmol) according to the procedures described for compound **21**. This furnished the title compound (196 mg, 0.920 mmol, 92% yield) as yellow oil. ^{1}H NMR ($CDCl_3$, 400 MHz): δ 5.89 – 5.77 (m, 1H), 5.57 (dt, *J* = 10.2, 2.8 Hz, 1H), 4.39 (br s, 1H), 3.96 (br s, 1H), 3.54 (d, *J* = 6.4 Hz, 2H), 3.33 (br s, 1H), 2.84 (br s, 1H), 2.08 (br s, 1H) 1.86 (dt, *J* = 16.0 Hz, 4.0Hz, 1H), 1.36 (s, 9H). ^{13}C NMR ($CDCl_3$, 101 MHz) δ 158.67, 127.20, 125.01, 79.87, 64.33, 53.93, 38.13, 28.32, 24.75. LC-MS m/z: calculated for $C_{11}H_{19}NO_3$ $[M+H]^+$ 214.28, found: 214.65.

***tert*-Butyl (*S*)-6-((benzyloxy)methyl)-3,6-dihydropyridine-1(2*H*)-carboxylate (41).** The title compound was synthesized from alcohol **40** (360 mg, 1.69 mmol) according to the procedures described for compound **22**. This furnished the title compound (446 mg, 1.47 mmol, 87% yield) as yellow oil. ^{1}H NMR ($CDCl_3$, 400 MHz): δ 7.37 – 7.21 (m, 5H), 5.93 (br, 1H), 5.74 (d, *J* = 12.0 Hz, 1H), 4.58 – 4.50 (m, 3H), 4.19 – 4.06 (m, 1H), 3.56 (br, 2H), 3.04 – 2.83 (m, 1H), 2.19 (br, 1H), 1.97 (d, *J* = 12.0 Hz, 1H), 1.44 (s, 9H). ^{13}C NMR ($CDCl_3$, 101 MHz,) δ 154.74, 138.53, 128.43, 127.60, 127.55, 126.92, 125.99, 79.74, 73.14, 71.43, 51.96, 37.17, 28.56, 25.03. LC-MS m/z: calculated for $C_{18}H_{25}NO_3$

[M+H]$^+$ 304.40, found: 303.89.

(*S*)-6-((Benzyloxy)methyl)-1,2,3,6-tetrahydropyridine (42). The title compound was synthesized from **41** (500 mg, 1.65 mmol) according to the procedures described for compound **23**. This furnished the title compound (275 mg, 1.35 mmol, 82 % yield) as a yellow oil. ^{1}H NMR (400 MHz, $CDCl_3$): δ 7.34 – 7.29 (m, 4H), 7.19 – 7.23 (m, 1H), 5.86 - 5.81 (m, 1H), 5.54 – 5.50 (m, 1H), 4.52 (d, *J* = 8.0 Hz, 2H), 3.59 – 3.55 (m, 1H), 3.46 (dd, *J* = 12.0, 4.0 Hz, 1H), 3.38 (app. t, *J* = 8.0 Hz, 1H), 3.07 – 3.01 (m, 1H), 2.86 – 2.79 (m, 1H), 2.36 (br s, 1H), 2.21 – 2.11(m, 1H), 2.06 – 1.91 (m, 1H). ^{13}C NMR (101 MHz, $CDCl_3$) δ 138.10, 128.25, 127.59, 127.49, 127.47, 126.66, 73.42, 73.19, 53.64, 41.15, 25.81. LC-MS m/z: calculated for $C_{13}H_{17}NO$ [M+H]$^+$ 204.29, found: 204.88.

***tert*-Butyl (*2S,3R*)-3-hydroxy-2-(hydroxymethyl)-3,6-dihydropyridine-1(2*H*)-carboxylate (44)**. Compound **43** was prepared according to the literature reported method[4]. The title compound was synthesized from **43** (280 mg, 1.1 mmol) and *p*-TsOH (28.0 mg, 0.15 mmol), according to the procedures described for compound **34**. This furnished the title compound (200 mg, 0.87 mmol, 84% yield) as a light yellow oil. ^{1}H NMR (400 MHz, $CDCl_3$) δ 5.87 – 5.81 (m, 1H), 5.77 (br, 1H), 4.38 – 4.28 (m, 1H), 4.12 (br, 2H), 3.89 (br, 2H), 3.53 – 3.33 (m, 3H), 1.37 (s, 9H). ^{13}C NMR (101 MHz, $CDCl_3$) δ 156.28, 127.16, 124.51, 80.31, 62.76, 60.64, 57.79, 41.08, 28.33. LC-MS m/z: calculated for $C_{11}H_{19}NO_4$ [M+H]$^+$ 230.28, found: 230.59.

***tert*-Butyl (*2S,3R*)-2-((benzyloxy)methyl)-3-hydroxy-3,6-dihydropyridine-1(2*H*)-carboxylate (45)**. Title compound was synthesized from compound **44** (280 mg, 1.22 mmol), benzyl bromide (0.140 mL, 1.18 mmol), KI (130 mg, 0.79 mmol) and K_2CO_3 (109 mg, 0.79 mmol), according to the procedures described for the preparation of compound **35**. This furnished title compound (188 mg, 0.590 mmol, 90% yield) as yellow oil. ^{1}H NMR (400 MHz, $CDCl_3$) δ 7.47 – 7.13 (m, 5H), 5.89 (br, 1H), 5.84 (br, 1H), 4.80 – 4.38 (m, 3H), 4.24 (br, 1H), 4.09 (br, 1H), 3.49 – 3.31 (m, 3H), 1.45 (s, 9H). ^{13}C NMR (101 MHz, $CDCl_3$) δ 155.72, 138.10, 128.30, 127.93, 127.56, 127.49, 124.72, 80.09, 72.73, 67.80, 63.56, 56.46, 40.19, 28.36. LC-MS m/z: calculated for $C_{18}H_{25}NO_4$ [M+H]$^+$ 320.40, found: 320.08.

***tert*-Butyl (*2S,3R*)-2-((benzyloxy)methyl)-3-((*tert*-butyldimethylsilyl)oxy)-3,6-dihydropyridine-1(2*H*)-carboxylate (46)**. The title compound was synthesized from compound **45** (570 mg, 1.78 mmol), and TBS-Cl (480 mg, 3.21 mmol), according to the procedures described for compound **36**. This furnished the title compound (740 mg, 1.70 mmol, 95% yield) as light yellow oil. ^{1}H NMR (400 MHz, $CDCl_3$) δ 7.34 – 7.25 (m, 5H), 5.79 (d, *J* = 11.4 Hz, 2H), 4.66 – 4.47 (m, 3H), 4.34 (br d, *J* = 20.0 Hz 1H), 4.19 (br, 1H), 3.37 (m, 3H), 1.46 (s, 9H), 0.90 (s, 9H), 0.11 (s, 3H), 0.08 (s, 3H). ^{13}C NMR (101 MHz,

$CDCl_3$) δ 155.40, 138.29, 128.32, 127.56, 127.38, 127.20, 124.66, 79.58, 72.72, 68.21, 64.35, 56.50, 39.94, 28.41, 25.91, 18.26, -4.36, -4.54. LC-MS m/z: calculated for $C_{24}H_{39}NO_4$ Si $[M+H]^+$ 434.66, found: 434.89.

(*2S,3R*)-2-((Benzyloxy)methyl)-3-((*tert*-butyldimethylsilyl)oxy)-1,2,3,6-tetrahydropyridine (47). The title compound was synthesized from compound **46** (210 mg, 0.484 mmol) according to the procedures described for compound **37**. This furnished crude product (120 mg, 0.344 mmol, 71% yield) as light yellow oil. ^1H NMR (400 MHz, $CDCl_3$) δ 7.43 – 7.39 (m, 4H), 7.38 – 7.32 (m, 1H), 5.85 – 5.80 (m, 1H), 5.71 (dd, *J* = 10.2, 2.1 Hz, 1H), 4.70 – 4.53 (m, 2H), 4.18 (d, *J* = 8.2 Hz, 1H), 3.80 (dd, *J* = 9.0, 2.8 Hz, 1H), 3.60 (dd, *J* = 9.0, 6.8 Hz, 1H), 3.53 – 3.45 (m, 1H), 3.37 – 3.31 (m, 1H), 2.85 – 2.81 (m, 1H), 2.61 (br, 1H), 0.95 (s, 9H), 0.15 (s, 3H), 0.11 (s, 3H). ^{13}C NMR (101 MHz, $CDCl_3$) δ 138.17, 130.30, 128.44, 127.89, 127.84, 127.73, 73.47, 71.06, 66.61, 59.02, 44.66, 29.72, 25.87, -4.09, -4.76. LC-MS m/z: calculated for $C_{19}H_{31}NO_2Si$ $[M+H]^+$ 334.55, found: 334.68.

S.Scheme 2. Enantioselective synthesis of compound **4a**

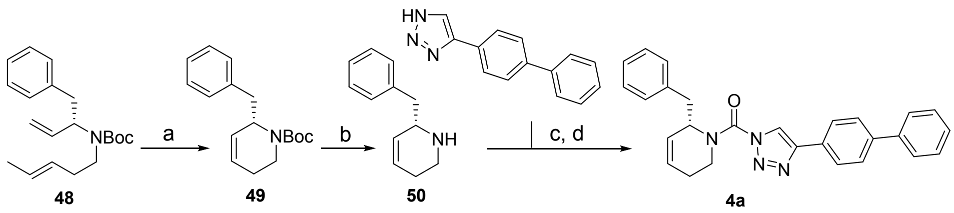

Reagents and conditions: (a) Grubbs I cat. 4 mol %, DCM, reflux, 48h; (b) 25% TFA, DCM; (c) DIPEA, triphosgene, THF, 0 °C; (d) DIPEA, DMAP, triazole , THF, 60 °C.

***tert*-Butyl (*S*)-6-benzyl-3,6-dihydropyridine-1(2*H*)-carboxylate (49)**. The title compound was prepared according to the literature reported method[2,3] as depicted in S.Scheme 2. In brief, the diene **48** (425 mg, 1.35 mmol) was dissolved in DCM (10 mL) and purged with argon. After the addition of Grubb's 1st generation catalyst (42.0 mg, 0.050 mmol, 3.6 mol%) and refluxing overnight TLC analysis confirmed complete conversion. The solvent was evaporated and the crude product purified by silica gel column chromatography using pentane : EtOAc = 97 : 3 as the eluent to afford the title compound (252 mg, 0.920 mmol, 68% yield). $[\alpha]_D^{21}$ = +161 (c = 1.0, $CHCl_3$). HRMS calculated for $C_{17}H_{23}NO_2$ $[M+H]^+$: 274.1802; found: 274.1802. ^1H NMR (400 MHz, $CDCl_3$, 60 °C) δ 7.28 – 7.21 (m, 2H), 7.20 – 7.14 (m, 3H), 5.79 (dd, *J* = 10.3, 6.1 Hz, 1H), 5.54 (dt, *J* = 10.3, 3.3 Hz, 1H), 4.54 (s, 1H), 4.11 (s, 1H), 2.89 (dd, *J* = 13.0, 6.2 Hz, 1H),

2.83 – 2.67 (m, 2H), 2.15 (m, 1H), 1.88 (m, 1H), 1.39 (s, 9H). ^{13}C NMR (101 MHz, $CDCl_3$, 60 °C) δ 154.28, 138.23, 129.36, 128.10, 127.94, 126.06, 125.38, 79.17, 53.47, 40.20, 36.30, 28.29, 24.85.

(*S*)-6-Benzyl-1,2,3,6-tetrahydropyridine (50) Boc-protected compound **49** (100 mg, 0.366 mmol) was added 25% TFA in DCM (5 mL), the reaction mixture was stirred at r.t. for 0.5h. The reaction mixture was co-evaporated with toluene (3 x 20 mL). The residue was diluted with ethyl acetate, washed with 10% Na_2CO_3, water, brine, dried over $MgSO_4$ and concentrated under reduced pressure. The crude product **50** was used without further purification. ^{1}H NMR (400 MHz, $CDCl_3$) δ 7.33 – 7.26 (m, 2H), 7.25 – 7.18 (m, 3H), 5.85 – 5.74 (m, 1H), 5.70 – 5.54 (m, 1H), 3.56 – 3.53 (m, 1H), 3.07 – 3.03 (m, 1H), 2.85 – 2.73 (m, 2H), 2.71 – 2.65 (m, 1H), 2.27 – 2.13 (m, 1H), 2.03 (br, 1H), 1.99 – 1.89 (m, 1H). ^{13}C NMR (101 MHz, $CDCl_3$) δ 138.99, 130.34, 129.30, 128.47, 126.32, 126.13, 55.46, 42.61, 42.15, 25.91.

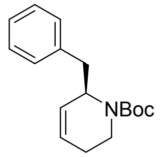

***tert*-Butyl (*R*)-6-benzyl-3,6-dihydropyridine-1(2*H*)-carboxylate (51)**. The title compound was prepared as described for the preparation of **49**, and afforded compound **51** (491 mg, 1.80 mmol). $[\alpha]_D^{21}$ = -172 (c =1.0 $CHCl_3$). HRMS calculated for $C_{17}H_{23}NO_2$ $[M+H]^+$: 274.1802; found: 274.1803. IR 2974, 2926, 1690, 1454, 1416, 1391, 1364, 1337, 1279, 1250, 1171, 1107. ^{1}H NMR (400 MHz, $CDCl_3$) δ 7.38 – 7.08 (m, 5H), 5.81 (s, 1H), 5.63 – 5.48 (m, 1H), 4.68 – 4.42 (m, 1H), 4.33 – 3.94 (m, 1H), 2.99 – 2.82 (m, 1H), 2.84 – 2.67 (m, 2H), 2.19 (m, 1H), 2.02 – 1.83 (m, 1H), 1.36 (s, 9H). ^{13}C NMR (101 MHz, $CDCl_3$) δ 154.34, 138.25, 129.40, 128.22, 127.70, 126.15, 125.63, 79.33, 53.84, 40.27, 35.87, 28.29, 24.85.

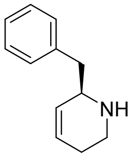

(*R*)-6-Benzyl-1,2,3,6-tetrahydropyridine (52). The title compound was prepared as described for **50** from *tert*-butyl (*R*)-6-benzyl-3,6-dihydropyridine-1(2*H*)-carboxylate **51** (130 mg, 0.476 mmol). The crude product was obtained that was used without further purification. ^{1}H NMR (400 MHz, $CDCl_3$) δ 7.33 – 7.25 (m, 2H), 7.25 – 7.15 (m, 3H), 5.81 – 5.76 (m, 1H), 5.65 – 5.61 (m, 1H), 3.56 – 3.50 (m, 1H), 3.06 - 3.01 (m, 1H), 2.85 – 2.72 (m, 2H), 2.72 – 2.49 (m, 1H), 2.28 – 2.07 (m, 1H), 1.99 – 1.90 (m, 1H), 1.88 (br, 1H). ^{13}C NMR (101 MHz, $CDCl_3$) δ 138.95, 130.32, 129.22, 128.39, 126.24, 126.05, 55.40, 42.58, 42.10, 25.88.

Chiral analysis for the representative enantiomers (Compound **11** and its enantiomer)

Compounds were analyzed on a Daicel Chiralcel OD-H column (4.5 X 250 mm, 20:80 IPA/Hex, flow rate of 1 mL/min)

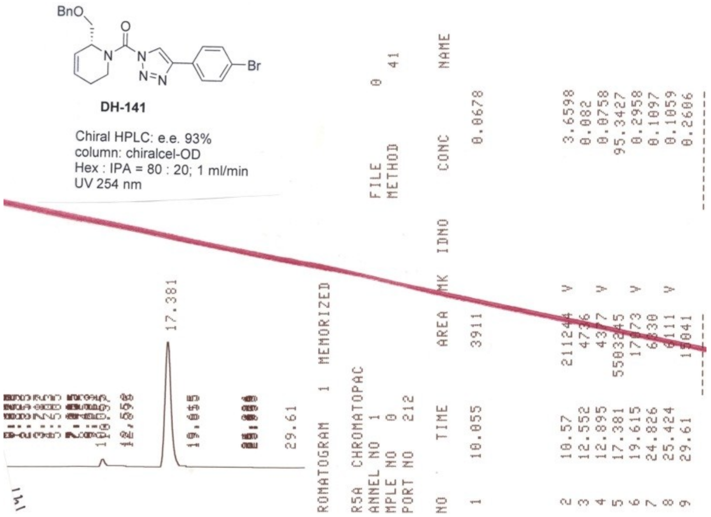

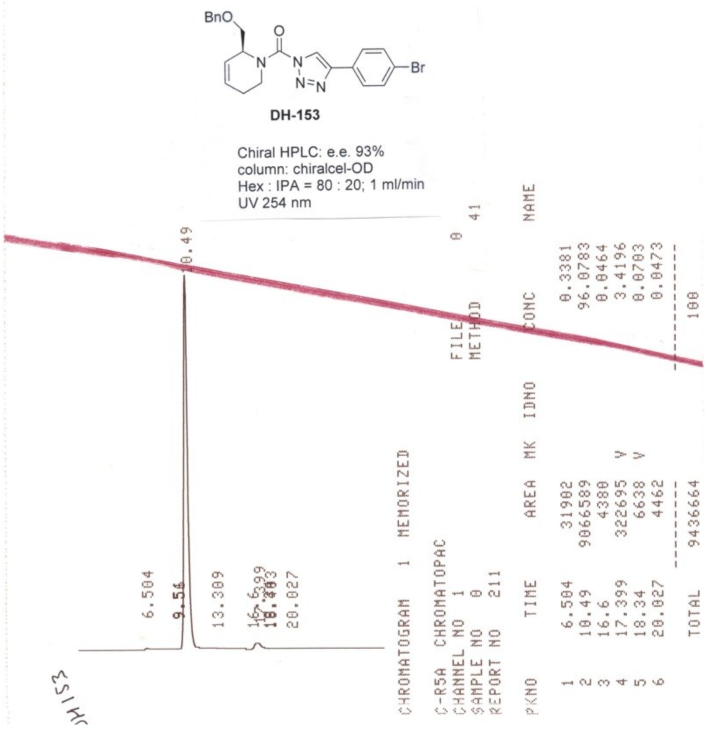

Chiral analysis for Compound **5a** and its enantiomer **5b**)

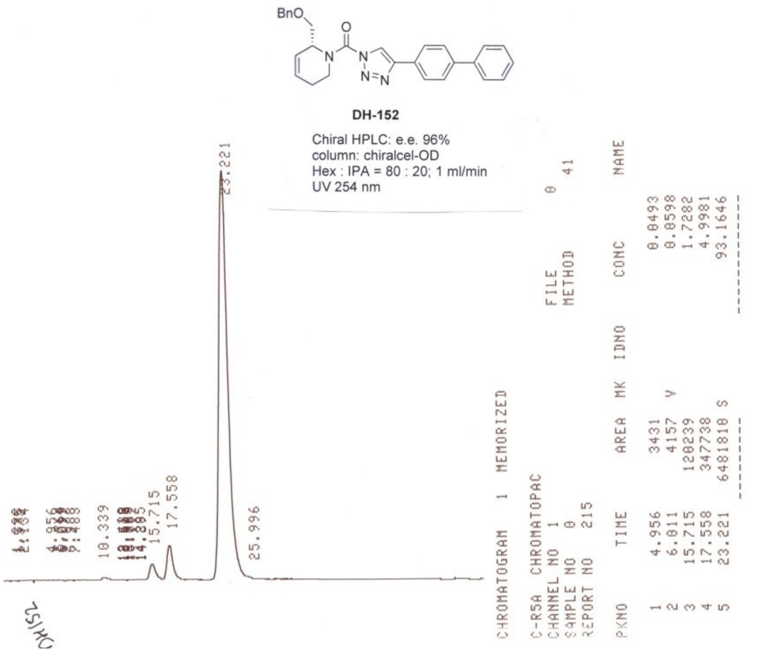

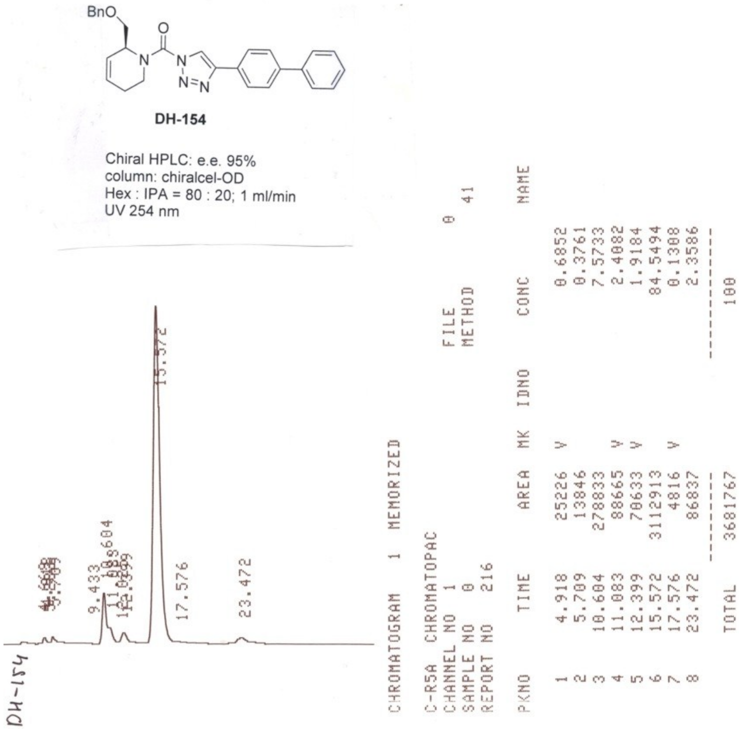

Since the existence of rotamers in the iminosugar-based trizole ureas complicates ^{1}H-NMR analysis, we conducted high-temperature (100 ^{o}C) NMR experiments by using compound **11** as an example. The purity of all final compounds was determined by HPLC system (Thermo Finnigan, buffer A: H_2O, B: acetonitrile (MeCN) and C: 1% aqueous TFA). The ^{1}H NMR spectrums and HPLC data are as follows:

^{1}H NMR

Compound **11** ($(CD_3)_2SO$, 25 ^{o}C)

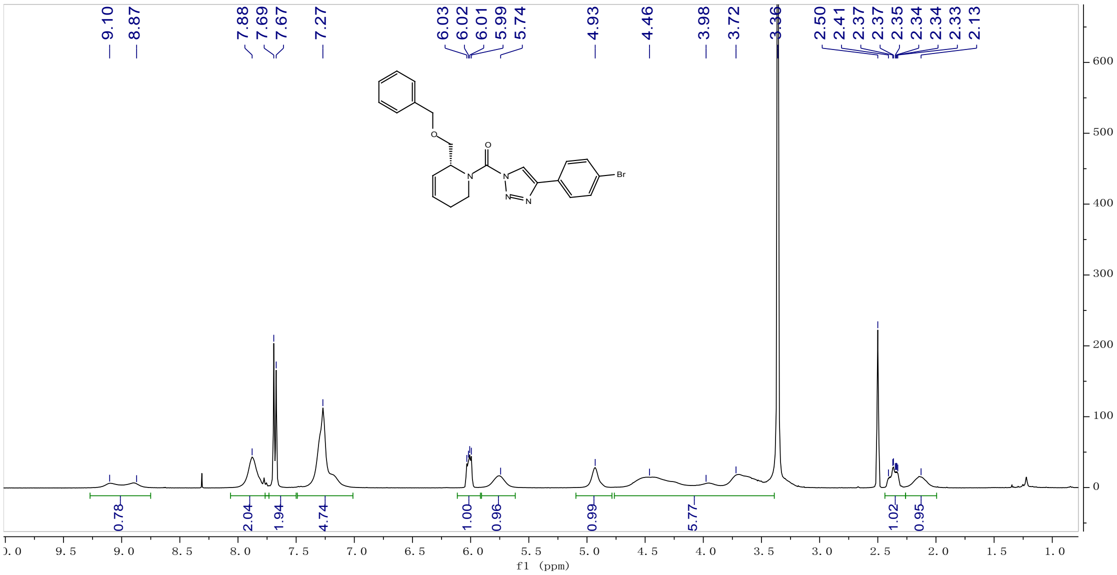

Compound **11** (($(CD_3)_2SO$, 100 °C)

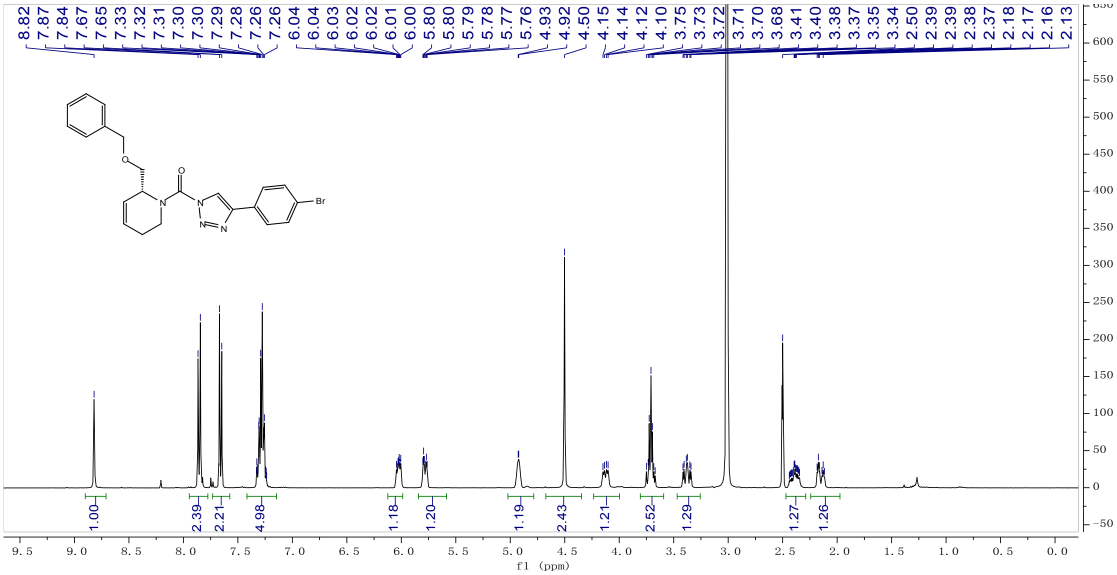

Compound 11

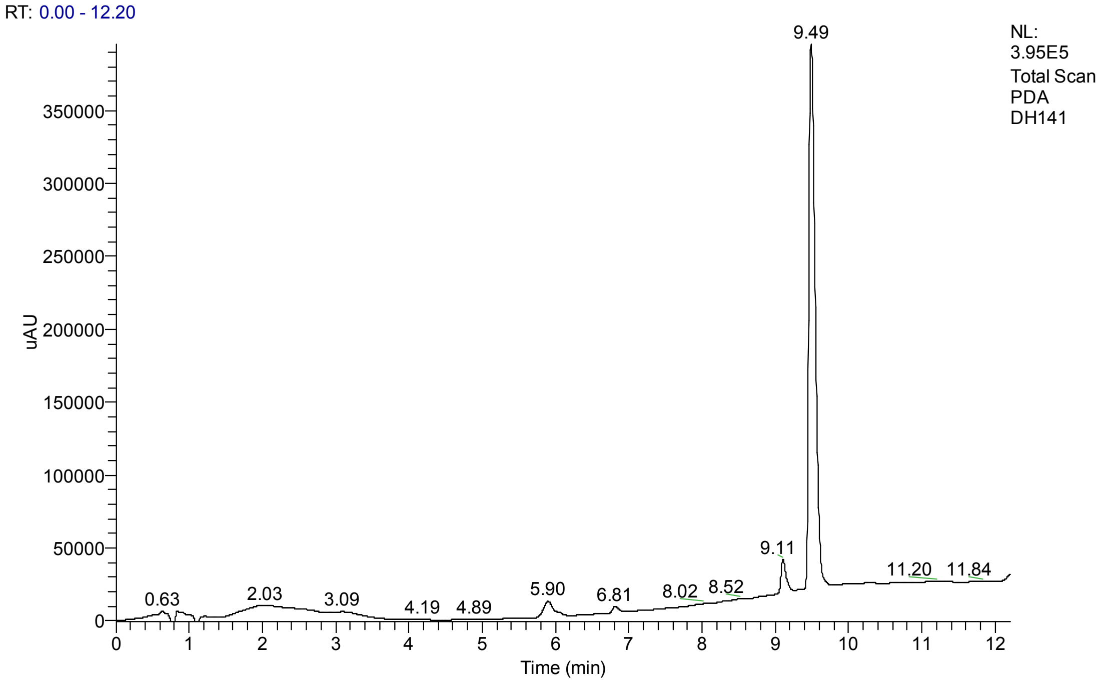

^{1}H NMR

Compound **3** ($CDCl_3$, 25 ^{o}C)

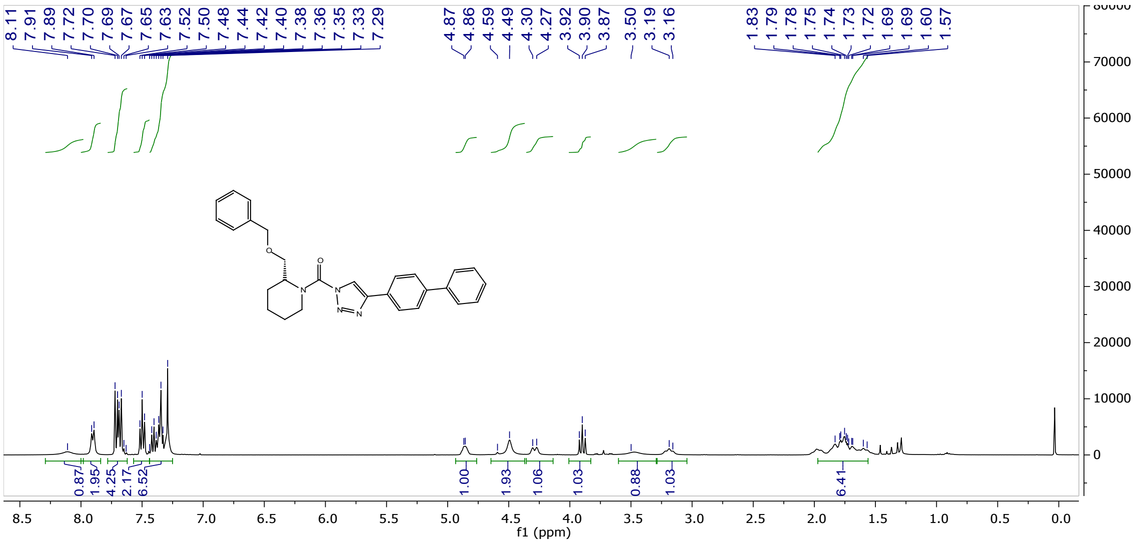

Compound **3**

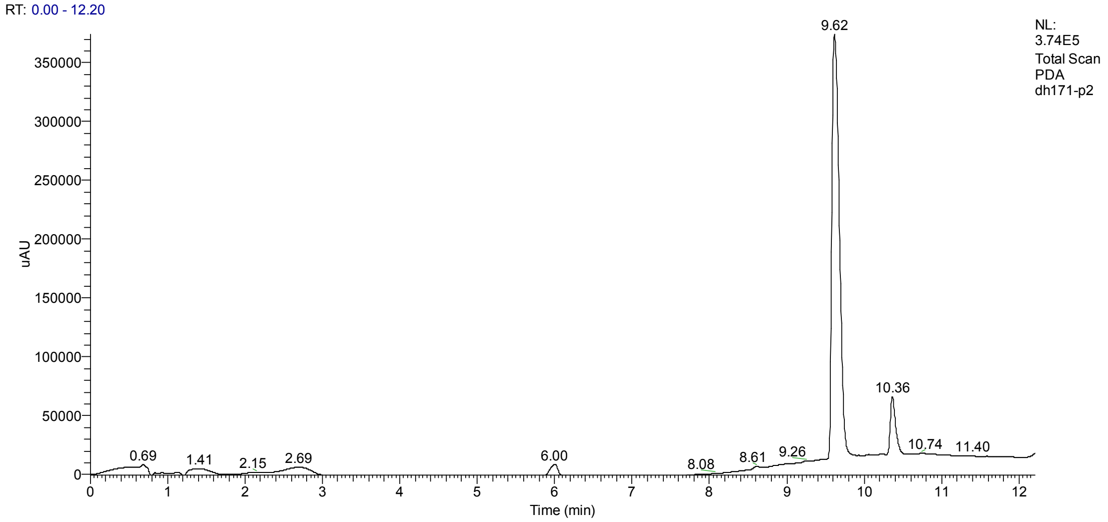

^{1}H NMR

Compound **4a** ($CDCl_3$, 25 °C)

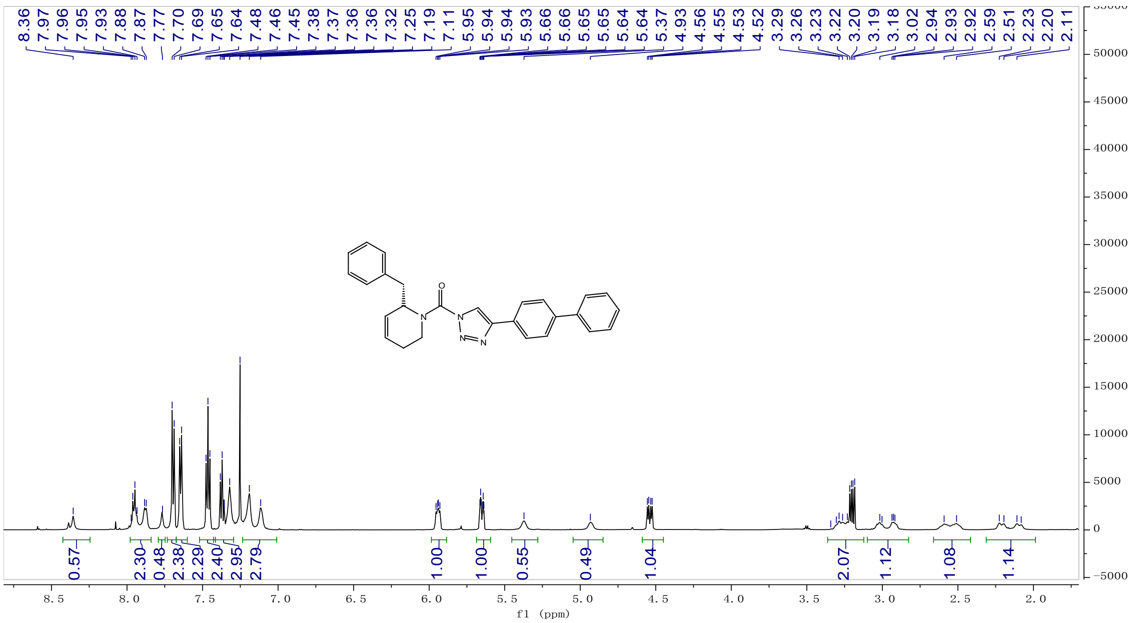

Compound **4a**

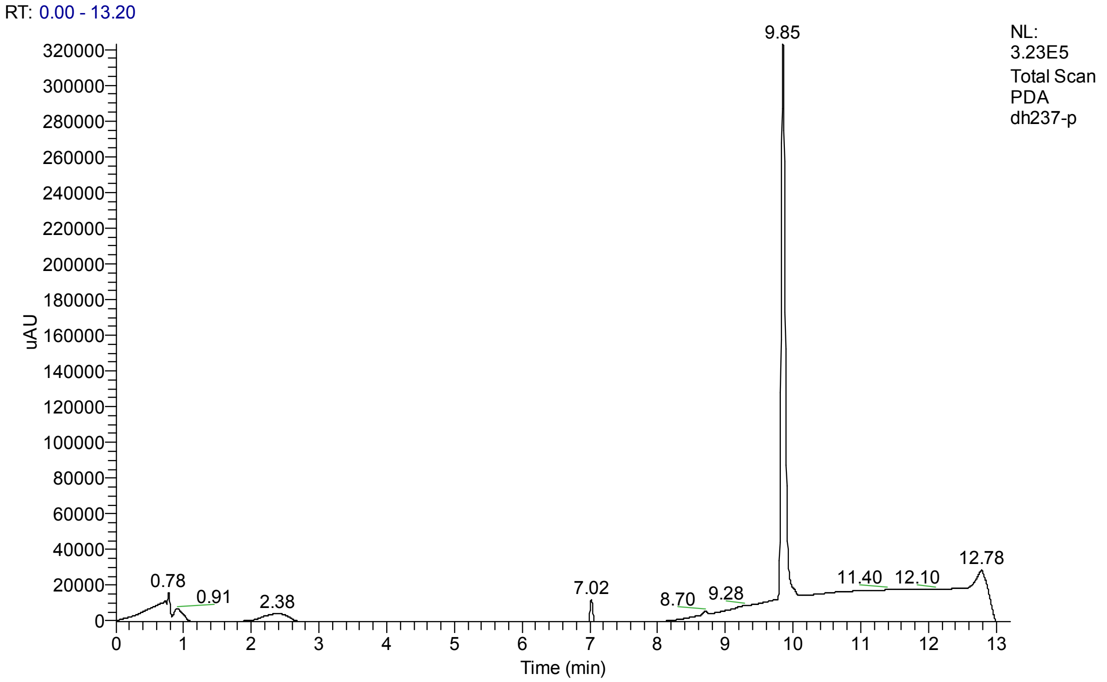

^{1}H NMR

Compound **5a** ($CDCl_3$, 25 ^{o}C)

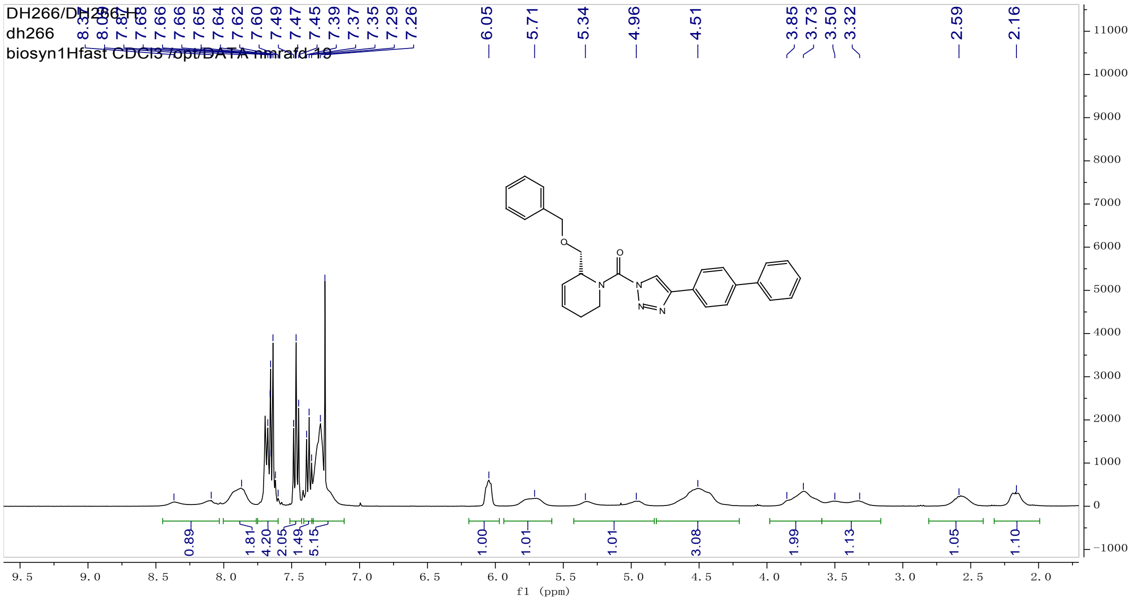

Compound **5a**

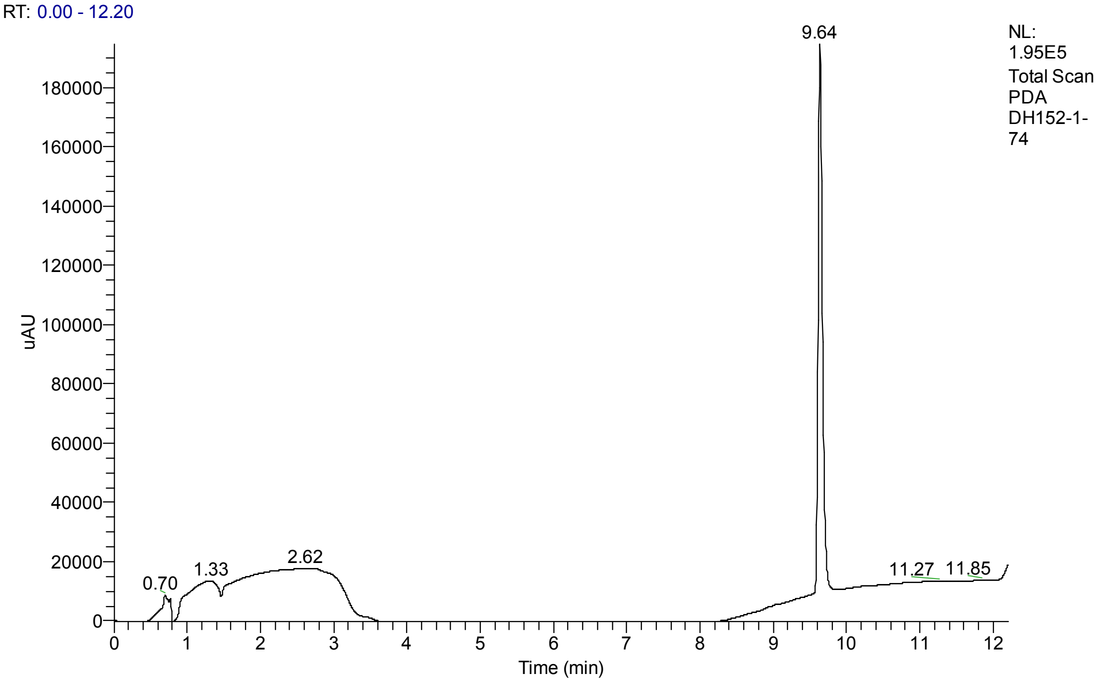

^{1}H NMR

Compound **6a** ($CDCl_3$, 25 °C)

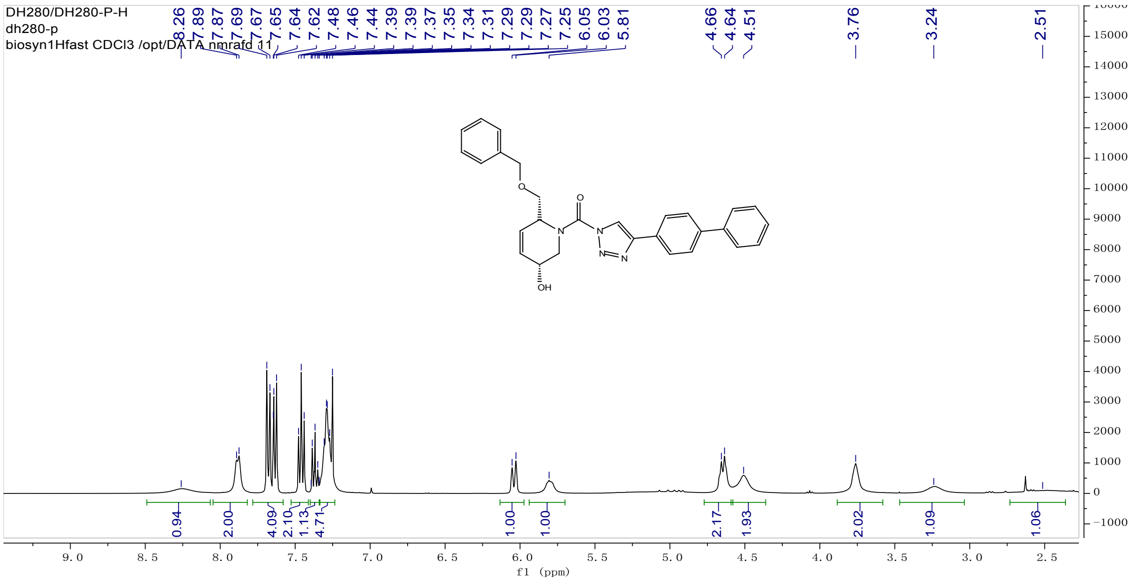

Compound **6a**

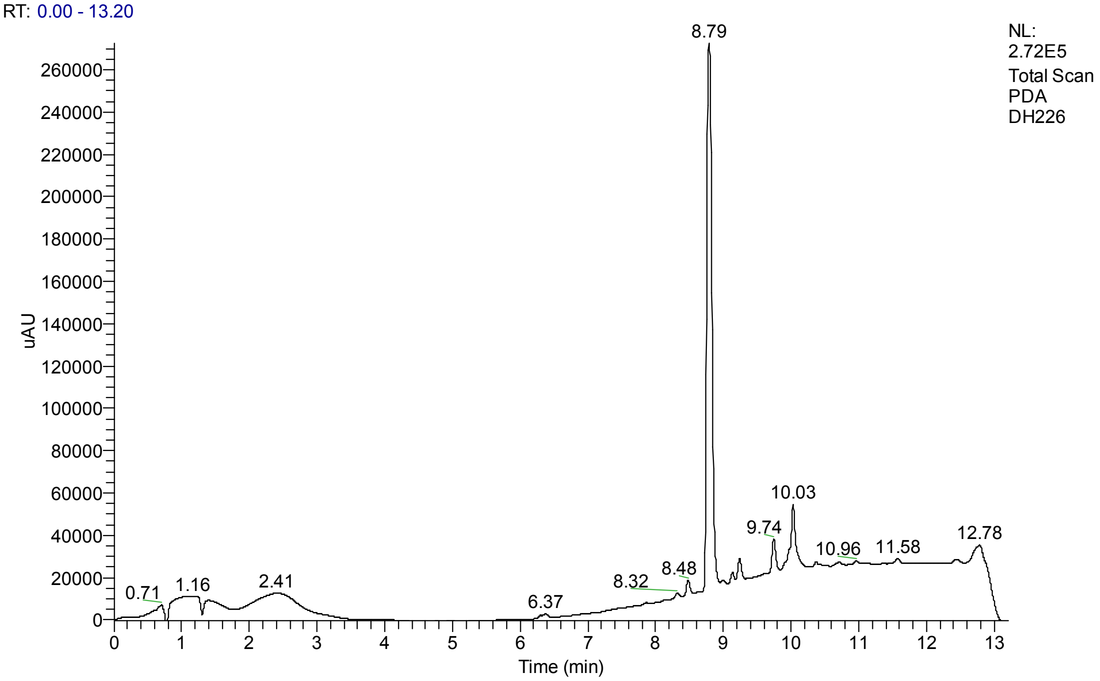

^{1}H NMR

Compound **7a** ($CDCl_3$, 25 °C)

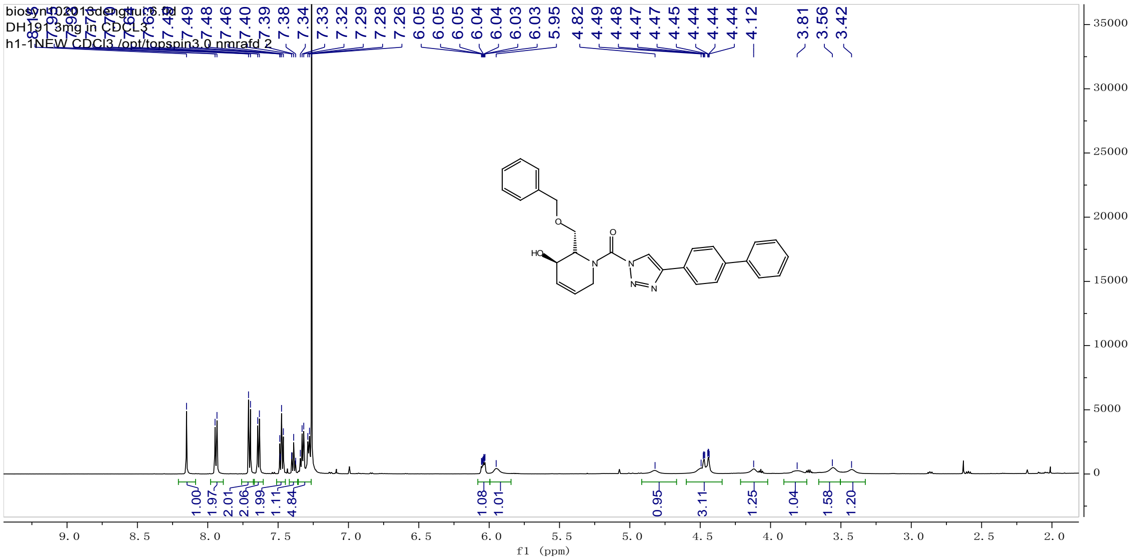

Compound **7a**

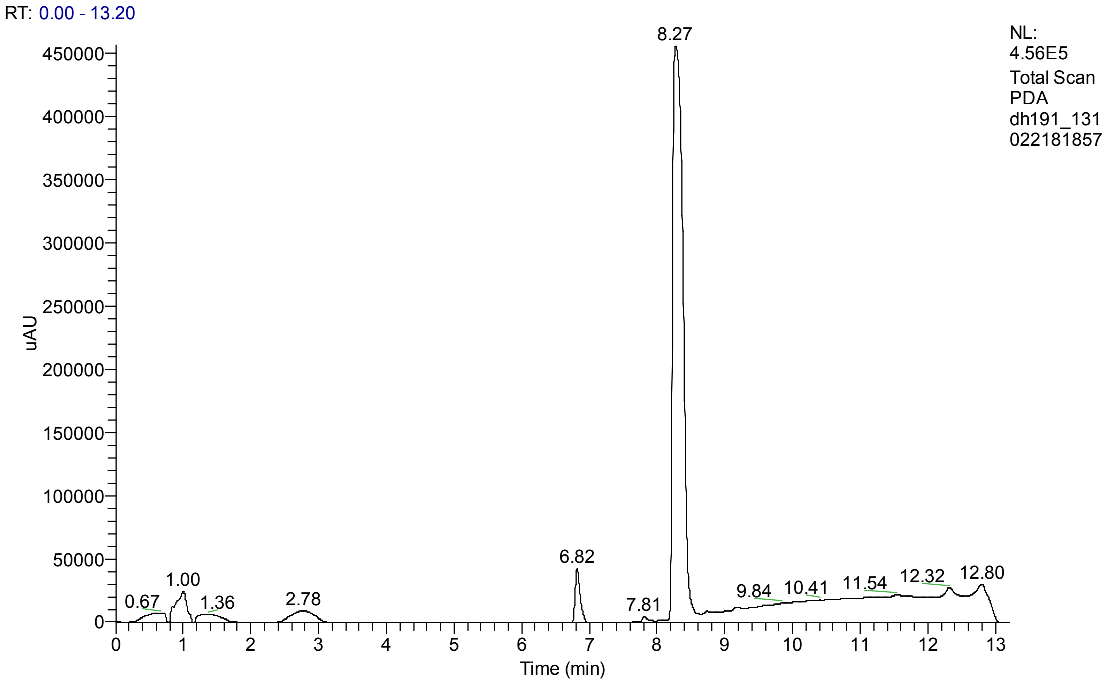

^{1}H NMR

Compound **8** ($CDCl_3$, 25 ^oC)

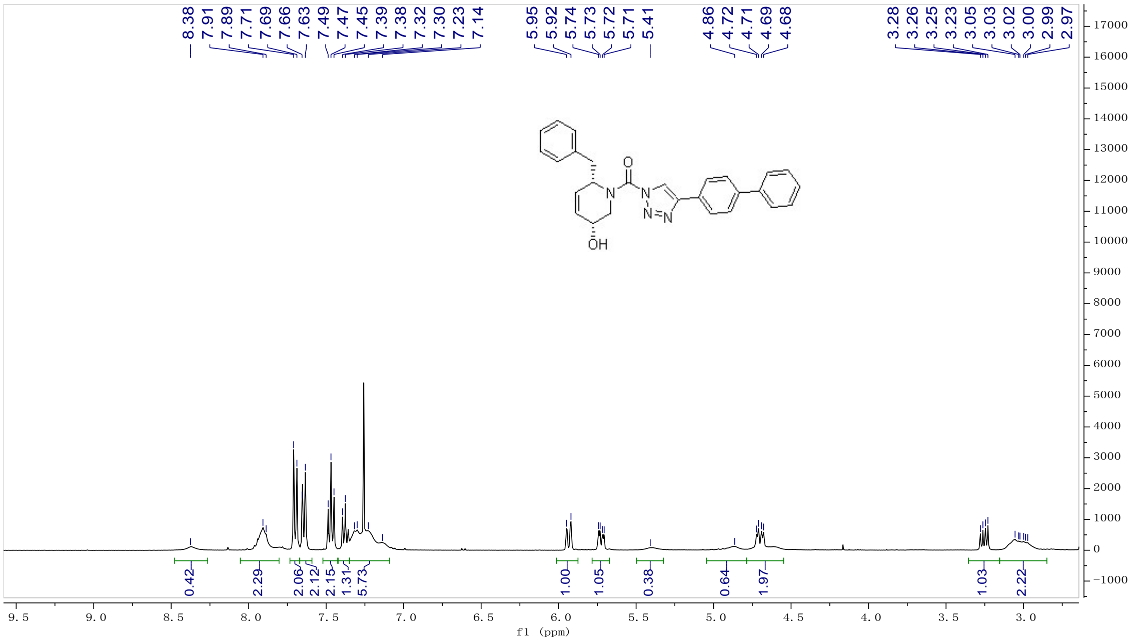

Compound **8**

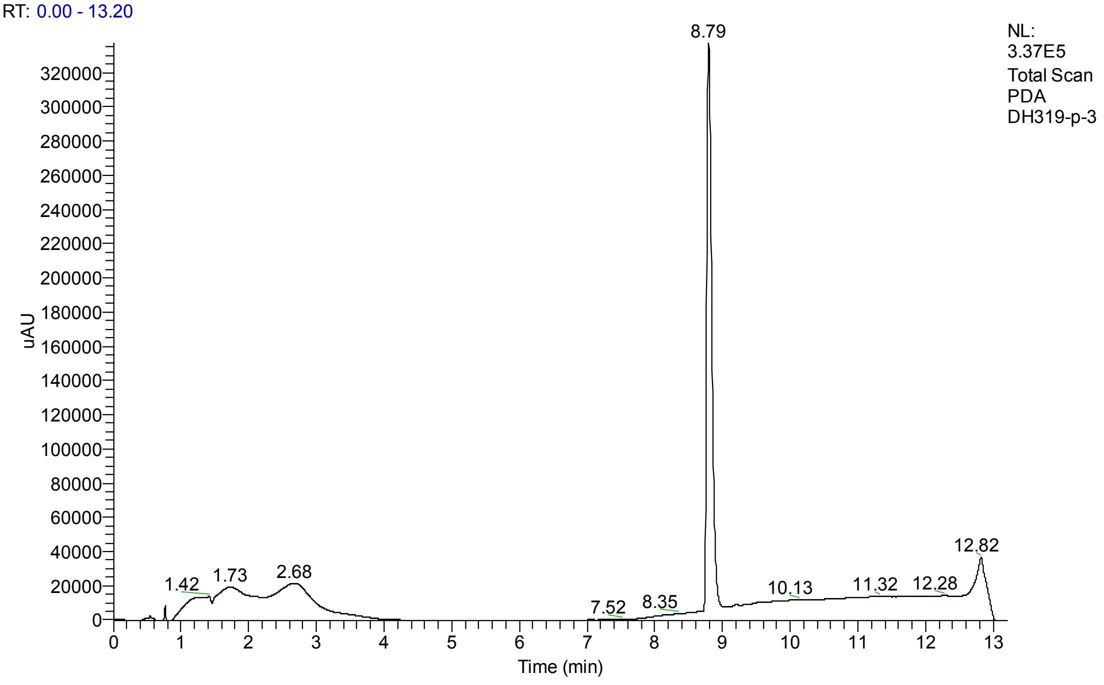

^{1}H NMR

Compound **4b** ($CDCl_3$, 25 ^{o}C)

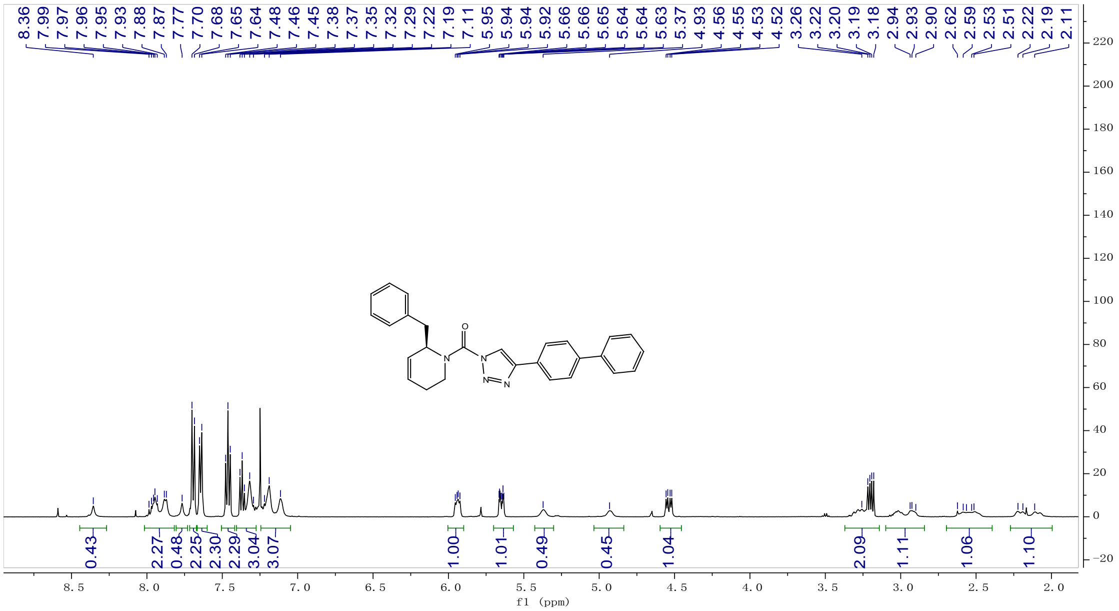

Compound **4b**

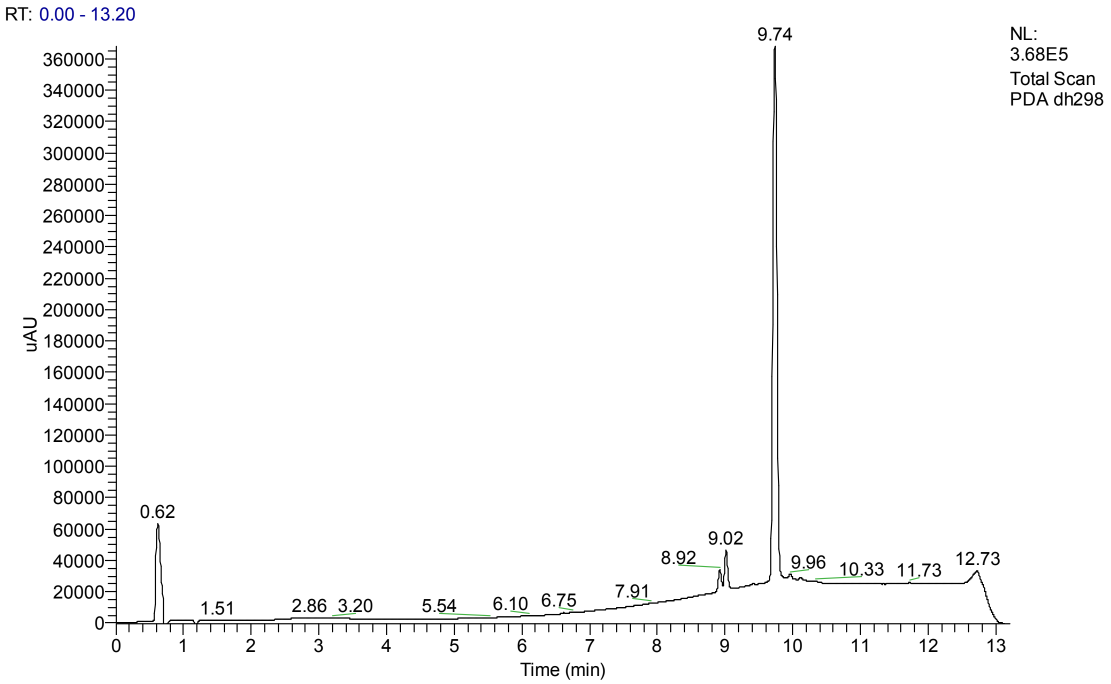

^{1}H NMR

Compound **5b** ($CDCl_3$, 25 ^{o}C)

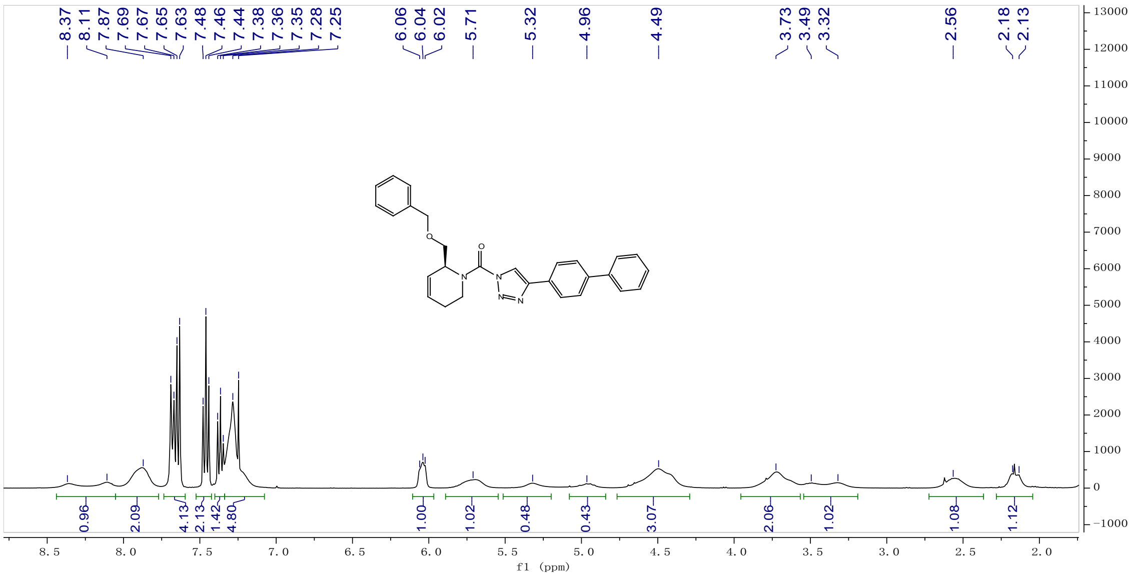

Compound **5b**

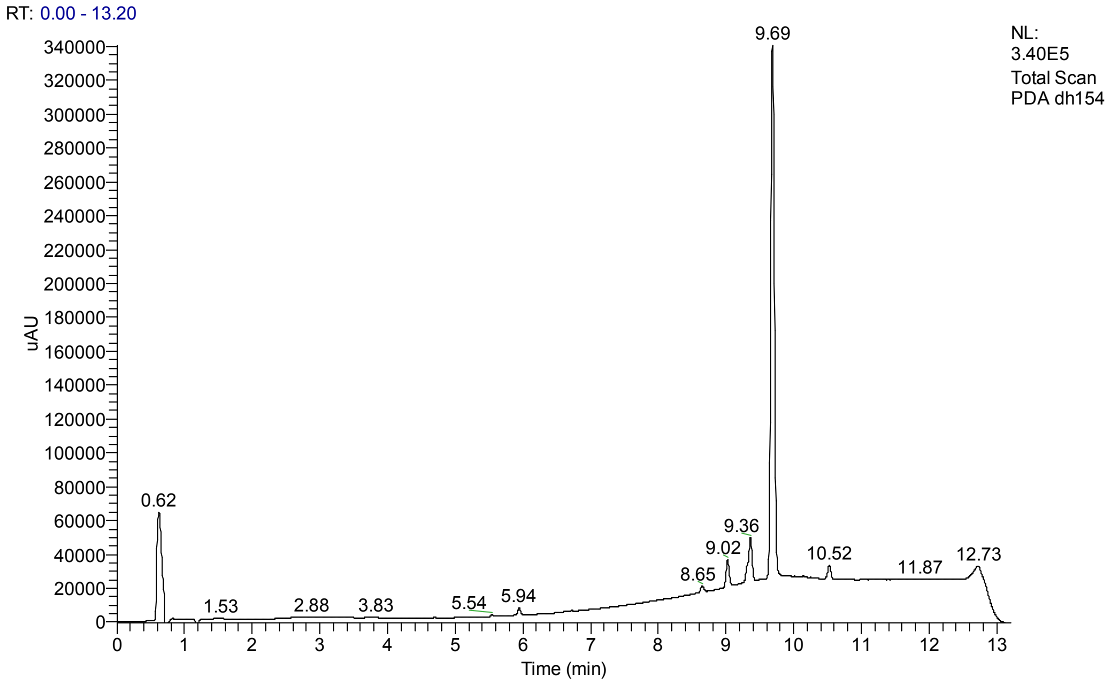

^{1}H NMR

Compound **6b** ($CDCl_3$, 25 ^oC)

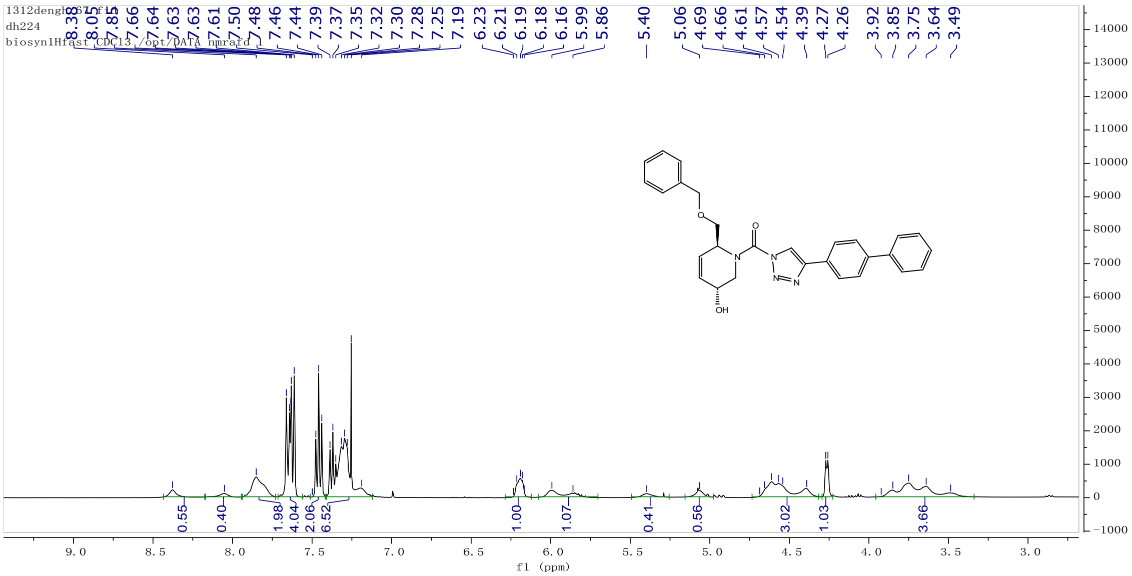

Compound **6b**

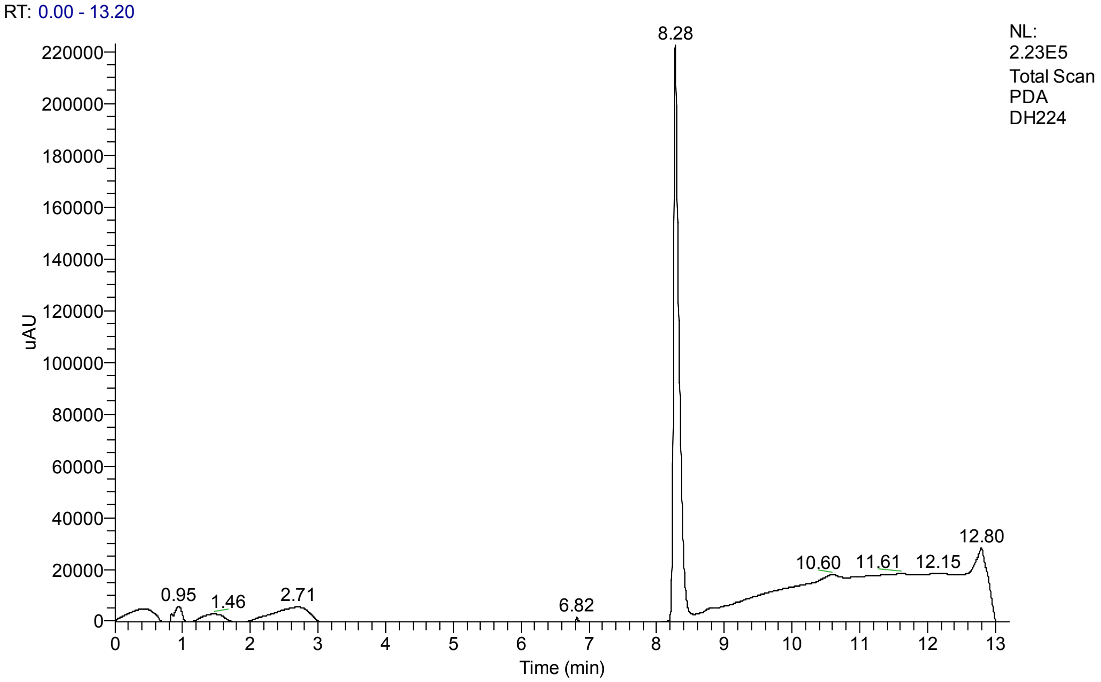

^{1}H NMR

Compound **6c** ($CDCl_3$, 25 °C)

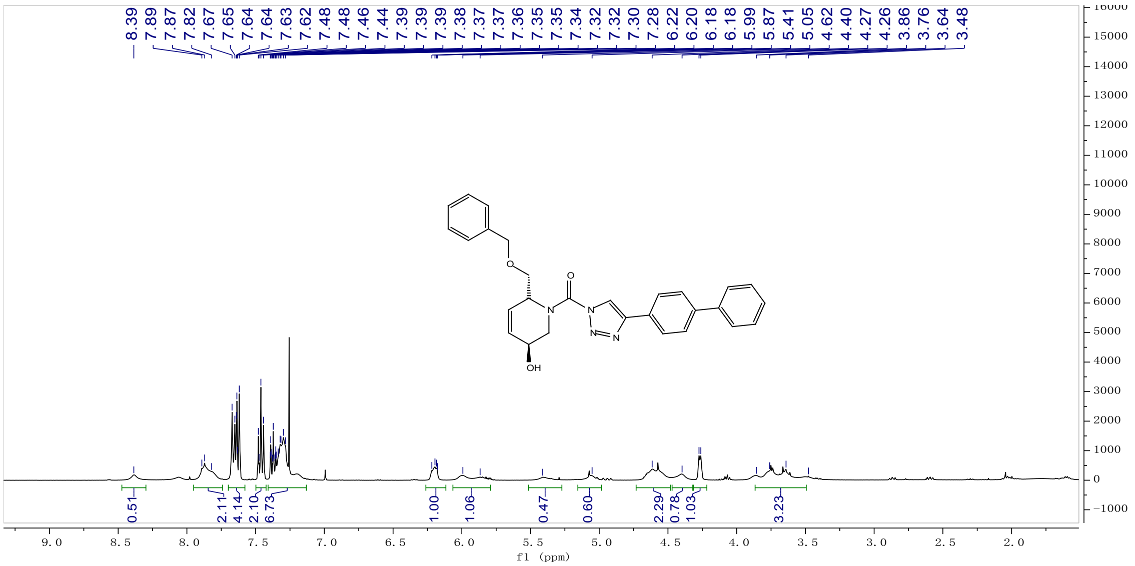

Compound **6c**

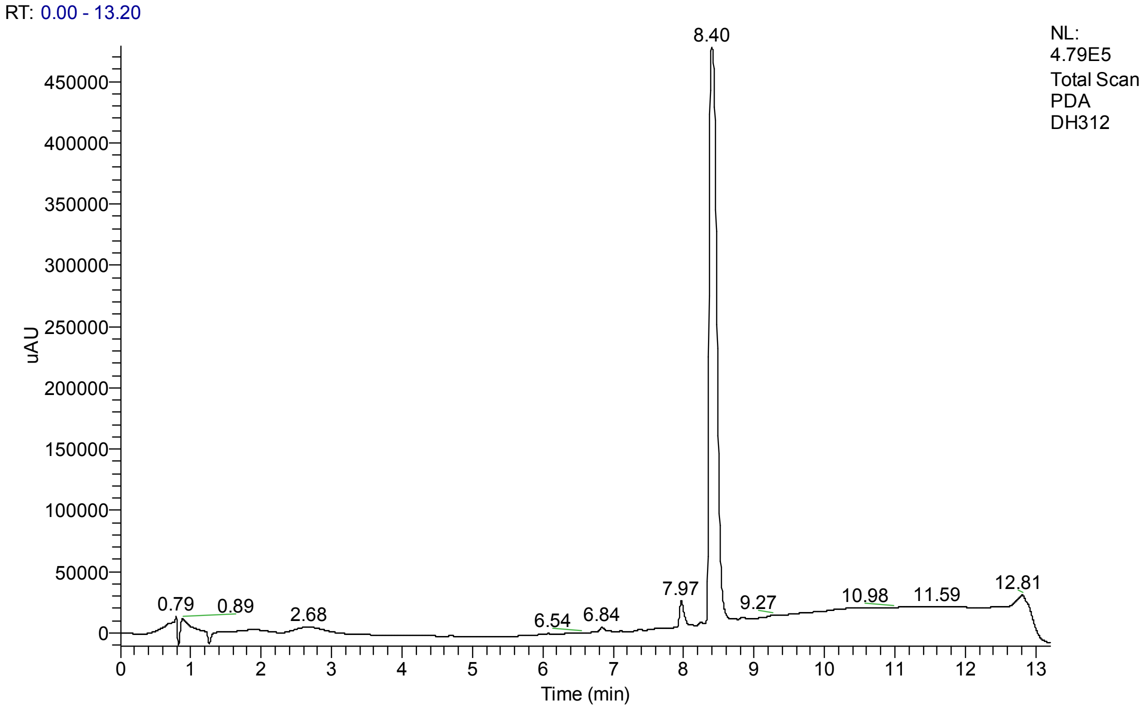

^{1}H NMR

Compound **7b** ($CDCl_3$, 25 ^{o}C)

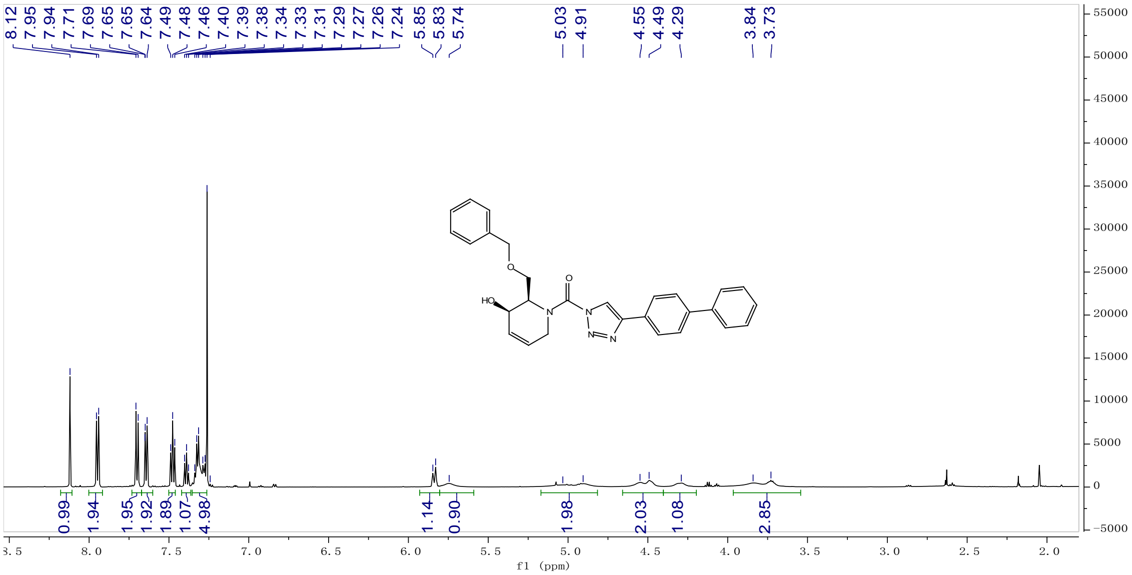

Compound **7b**

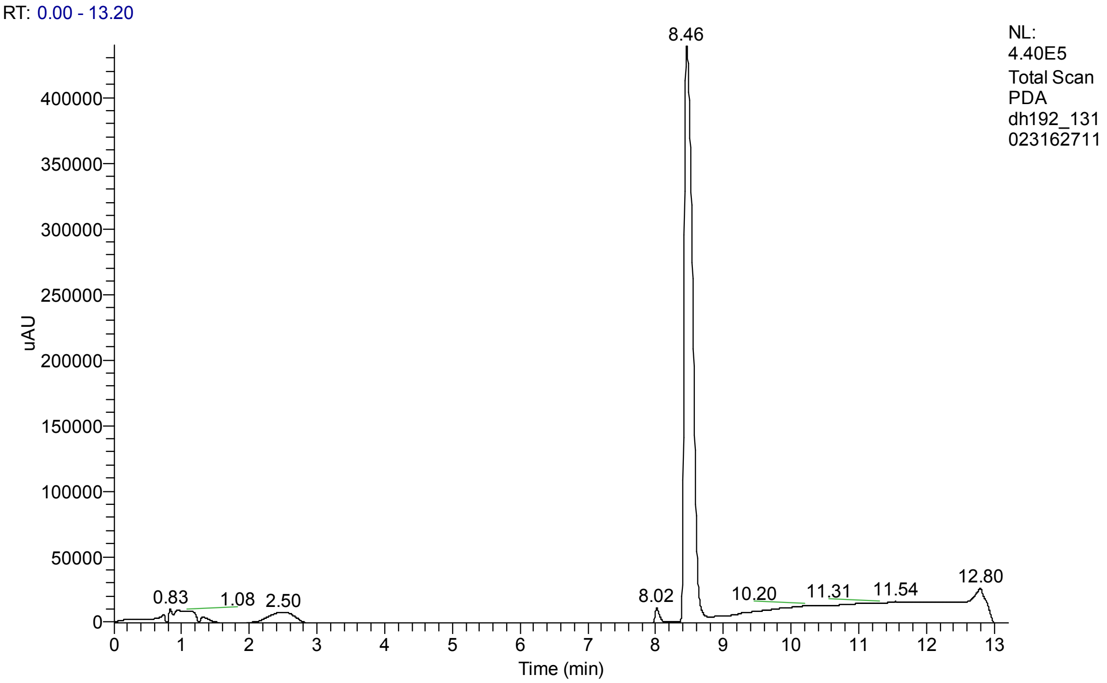

^{1}H NMR

Compound **9** ($CDCl_3$, 25 ^{o}C)

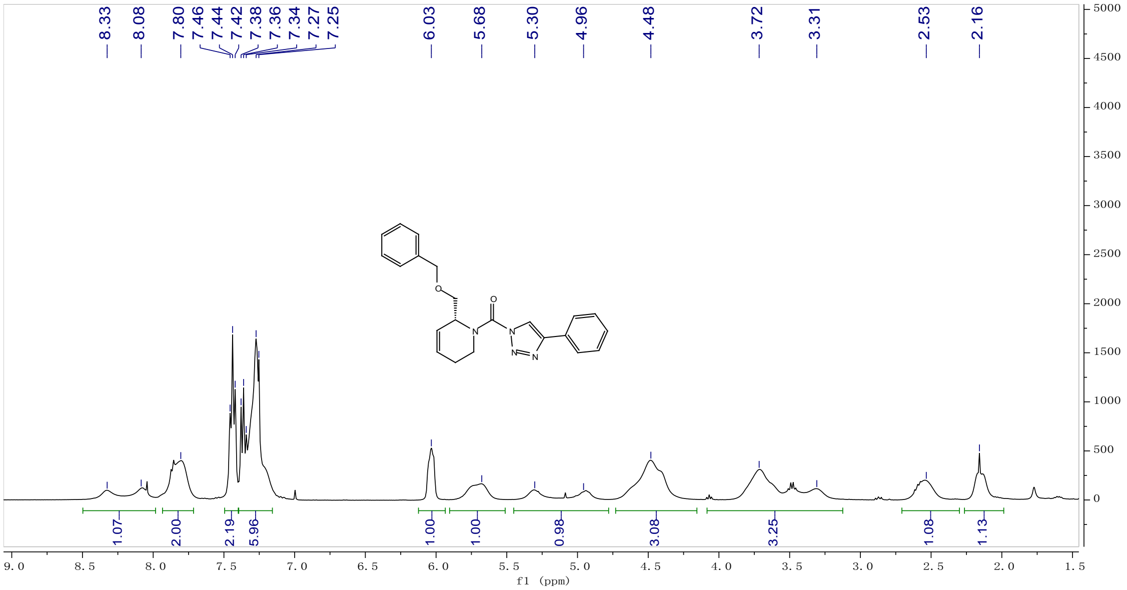

Compound **9**

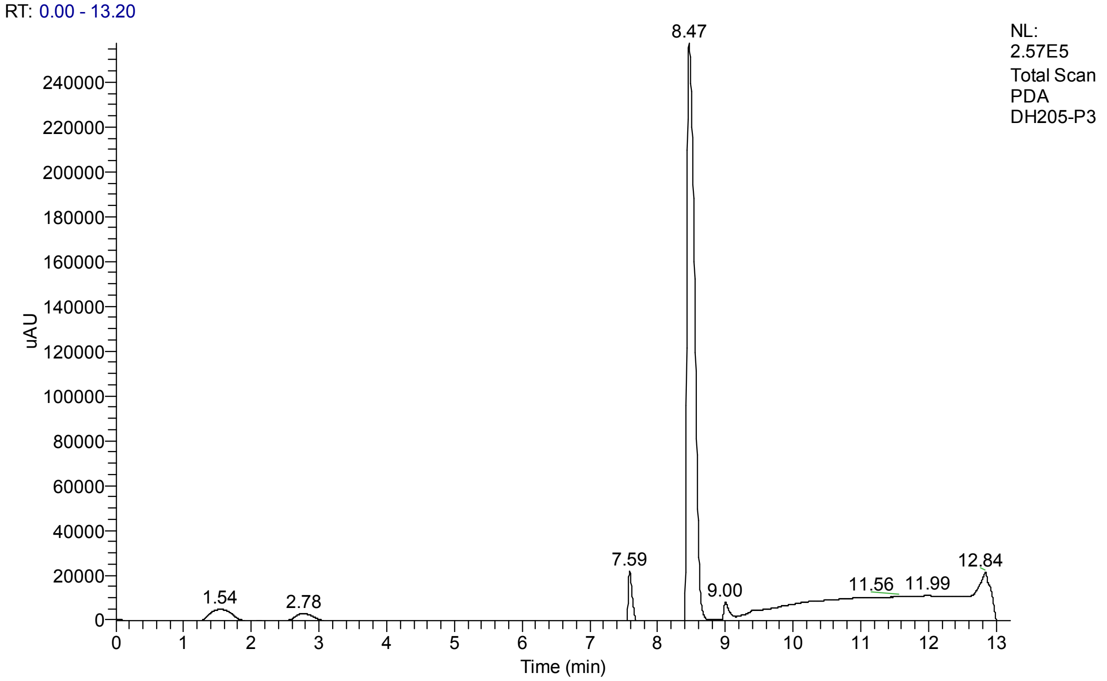

^{1}H NMR

Compound **10** ($CDCl_3$, 25 °C)

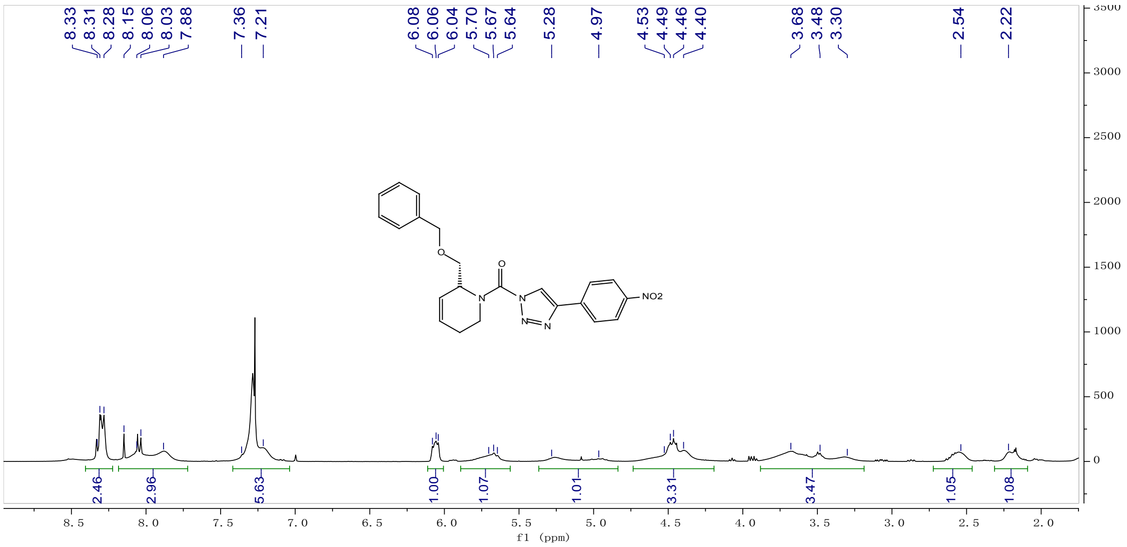

Compound **10**

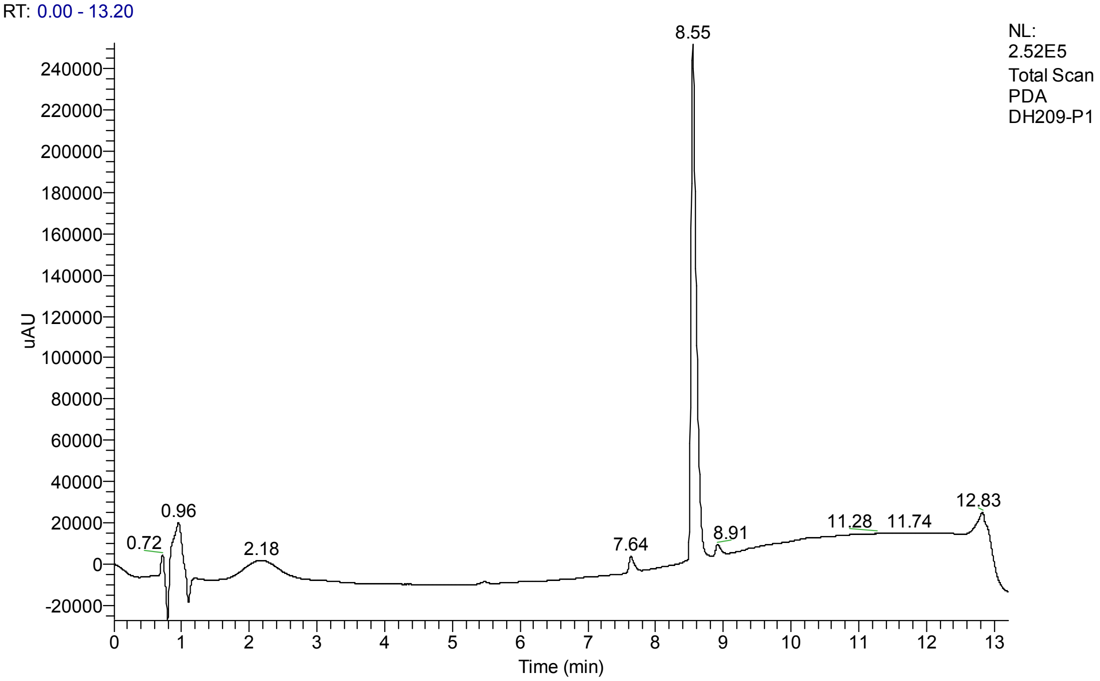

^{1}H NMR

Compound **12** ($CDCl_3$, 25 ^oC)

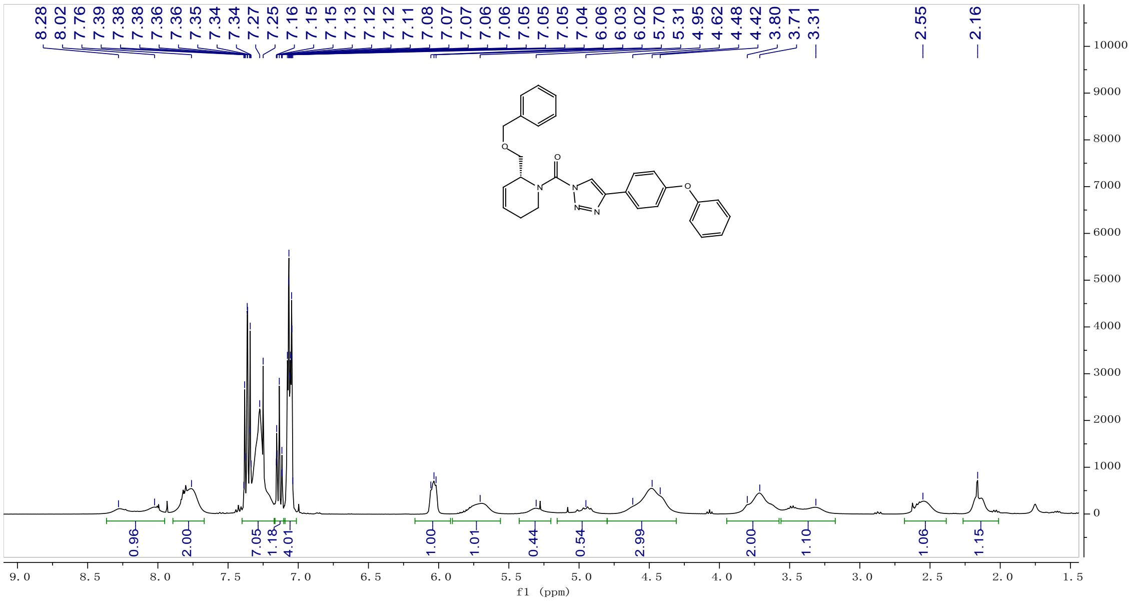

Compound **12**

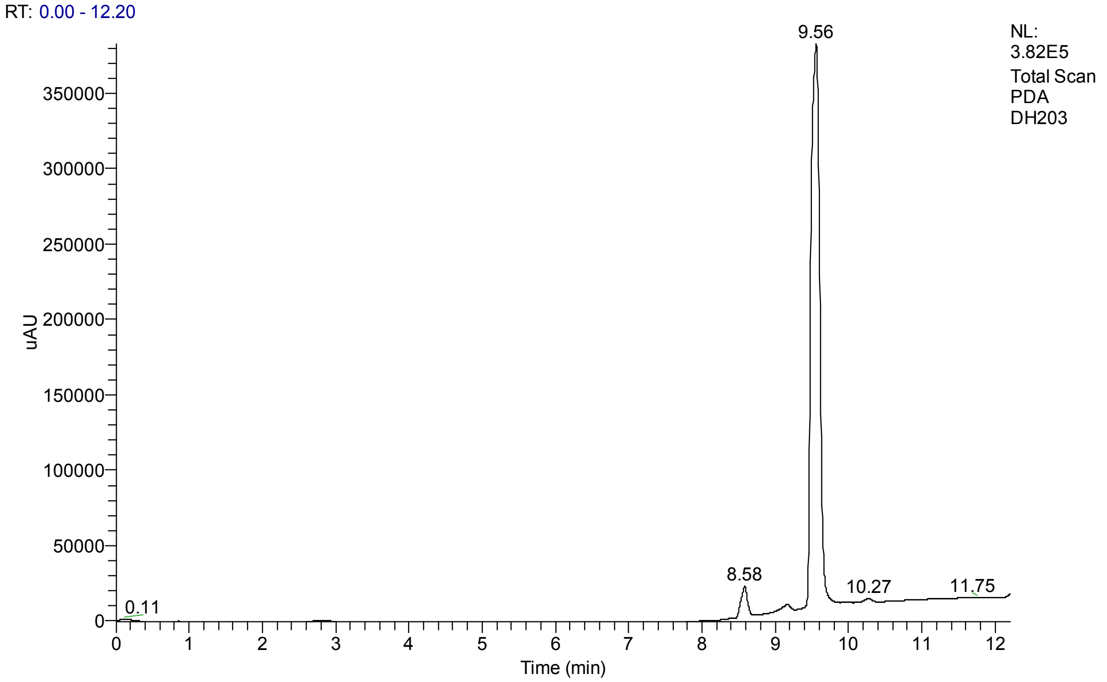

^{1}H NMR

Compound **13** ($CDCl_3$, 25 °C)

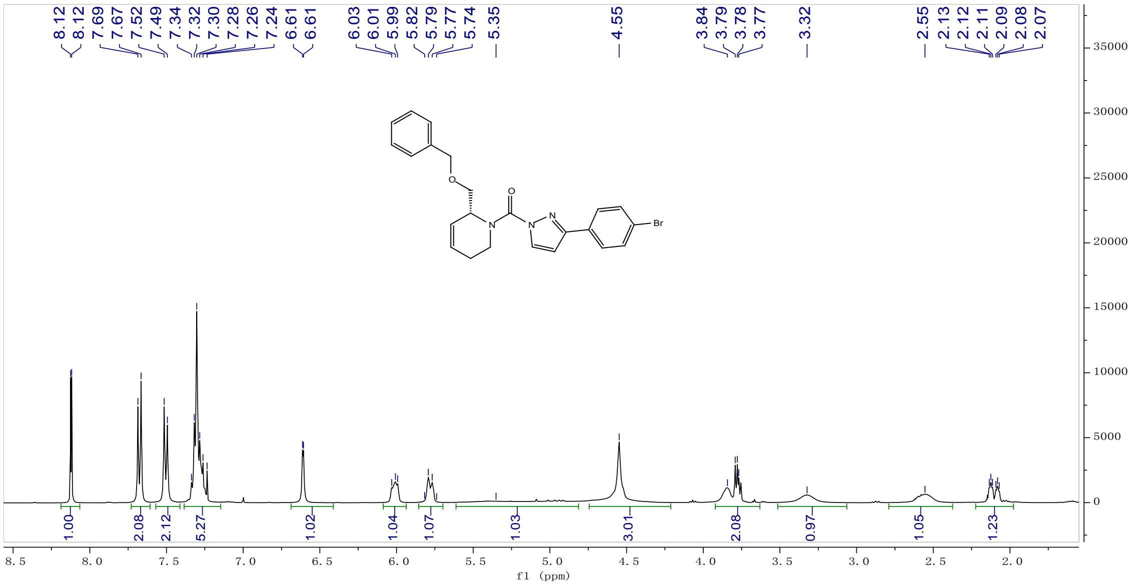

Compound **13**

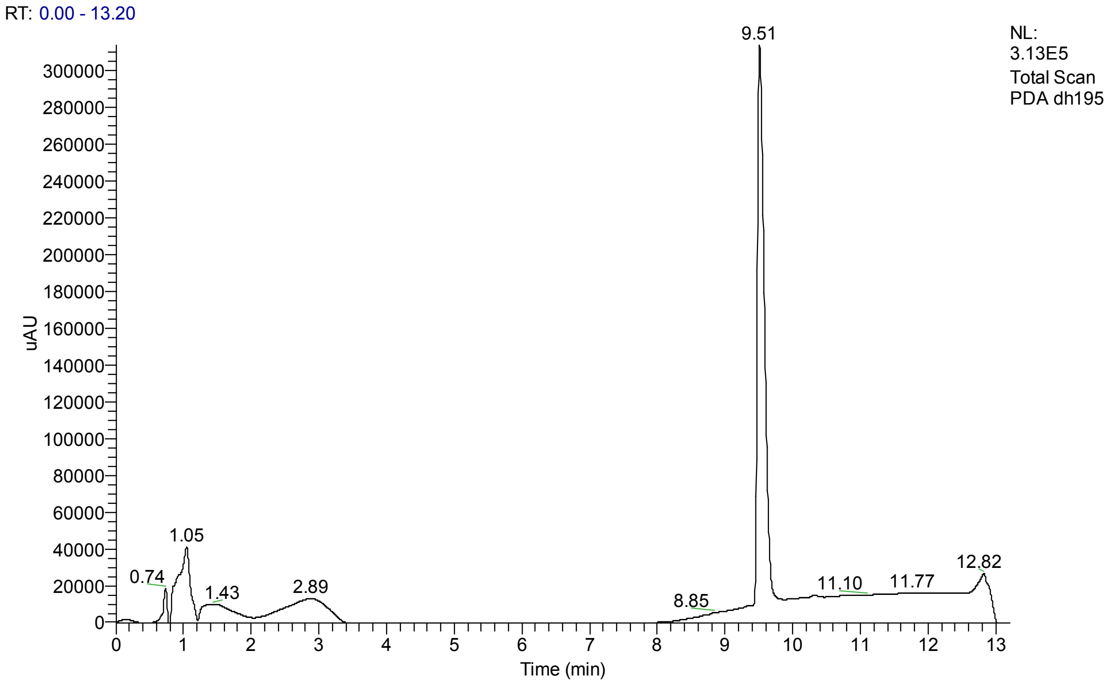

^{1}H NMR

Compound **14** ($CDCl_3$, 25 ^{o}C)

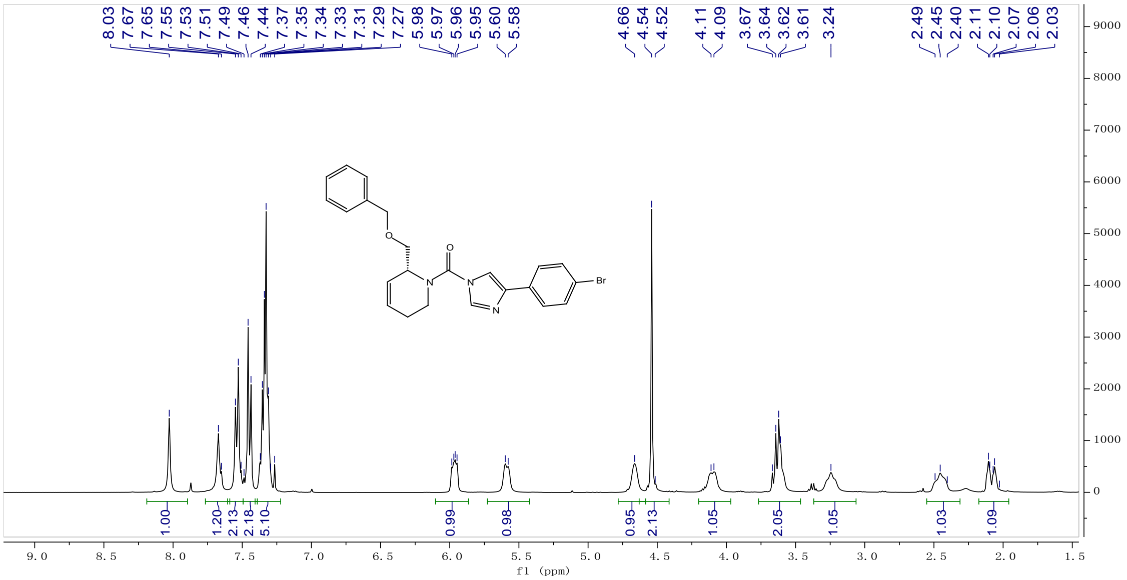

Compound **14**

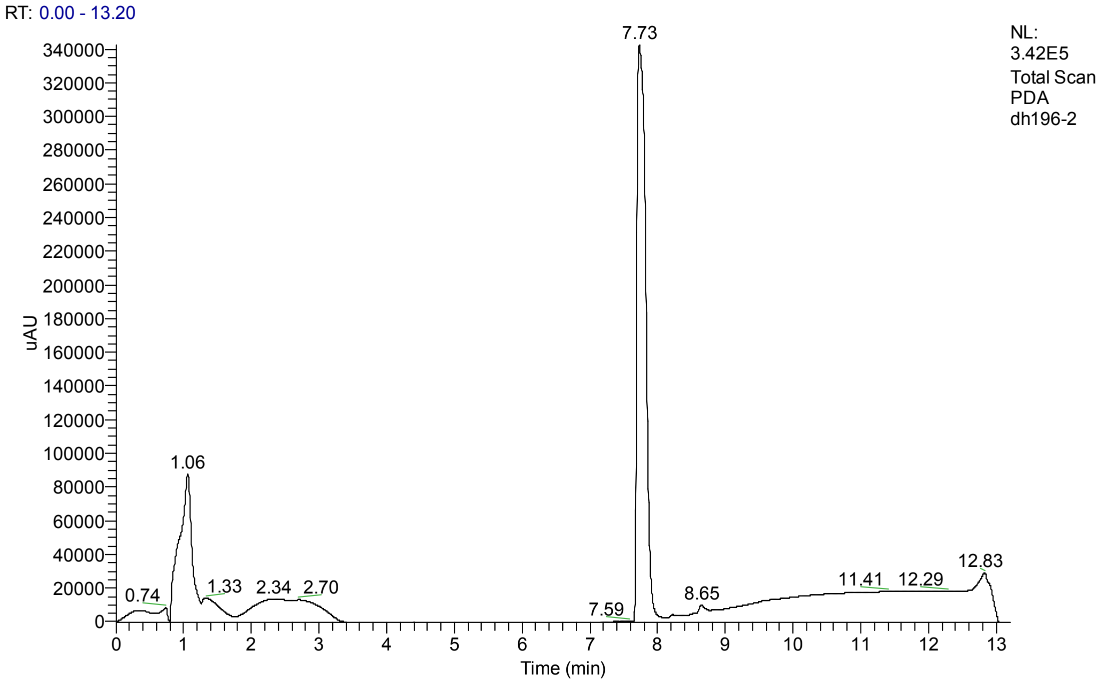

^{1}H NMR

Compound **15** ($CDCl_3$, 25 ^{o}C)

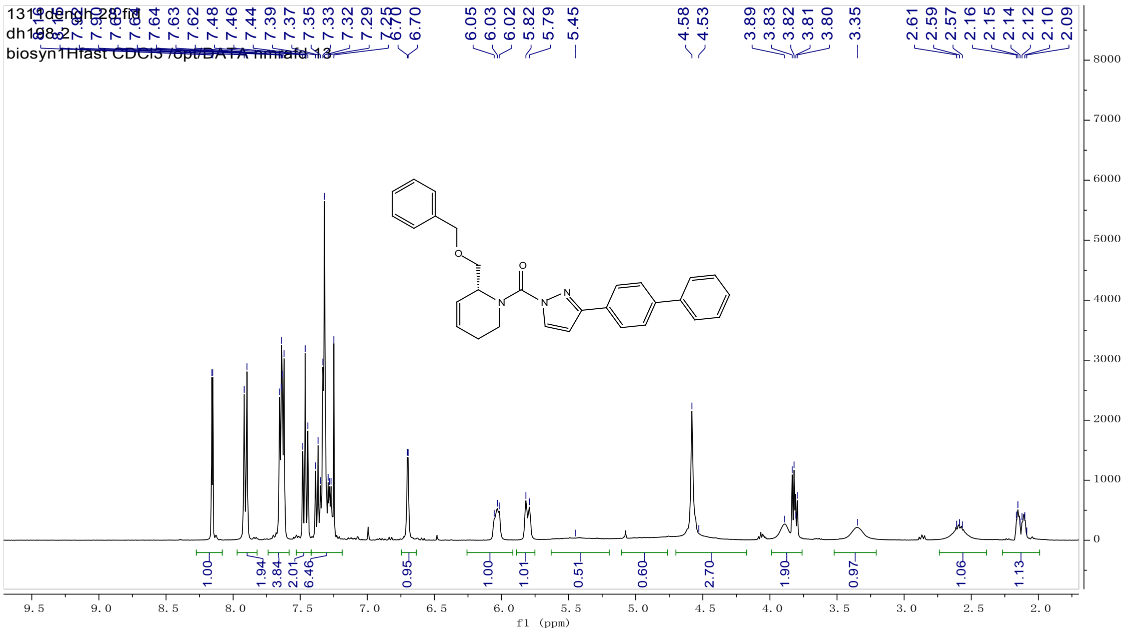

Compound **15**

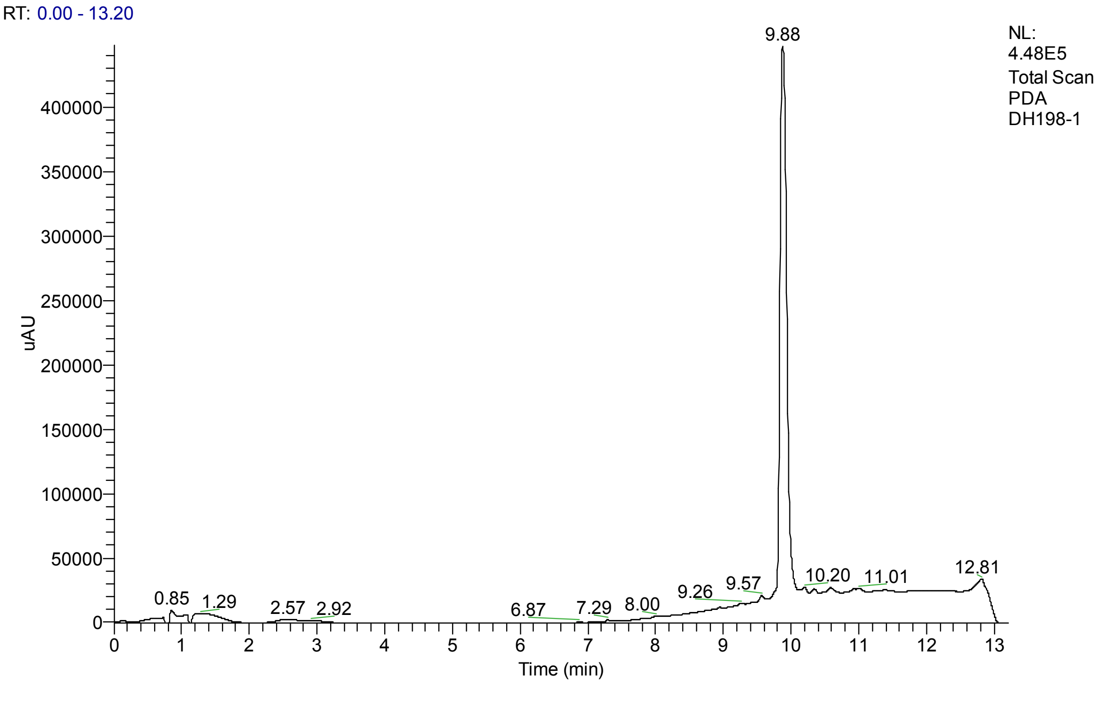

^{1}H NMR

Compound **16** ($CDCl_3$, 25 °C)

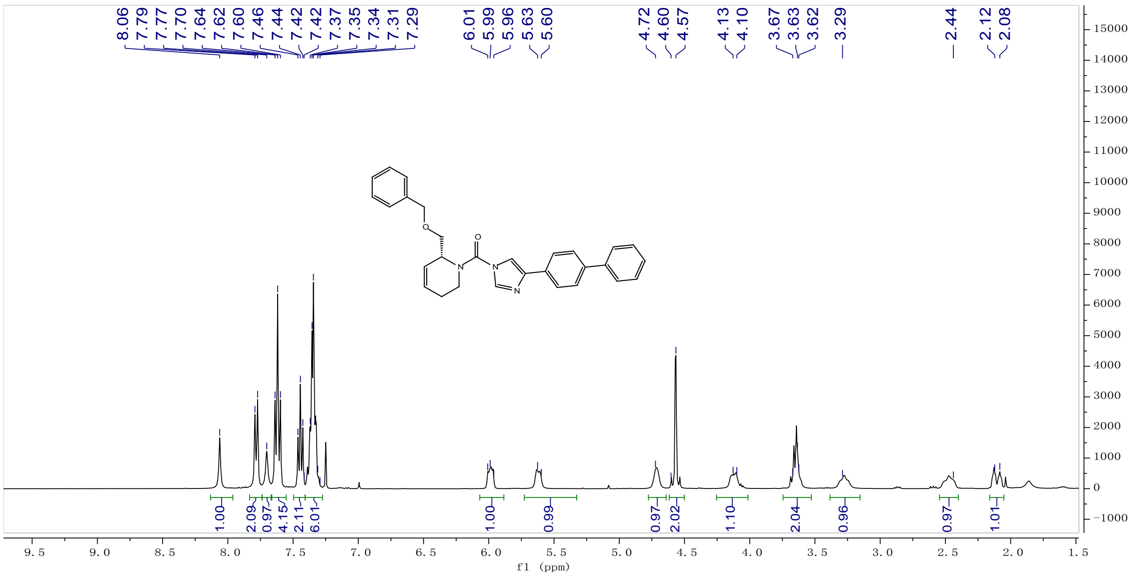

Compound **16**

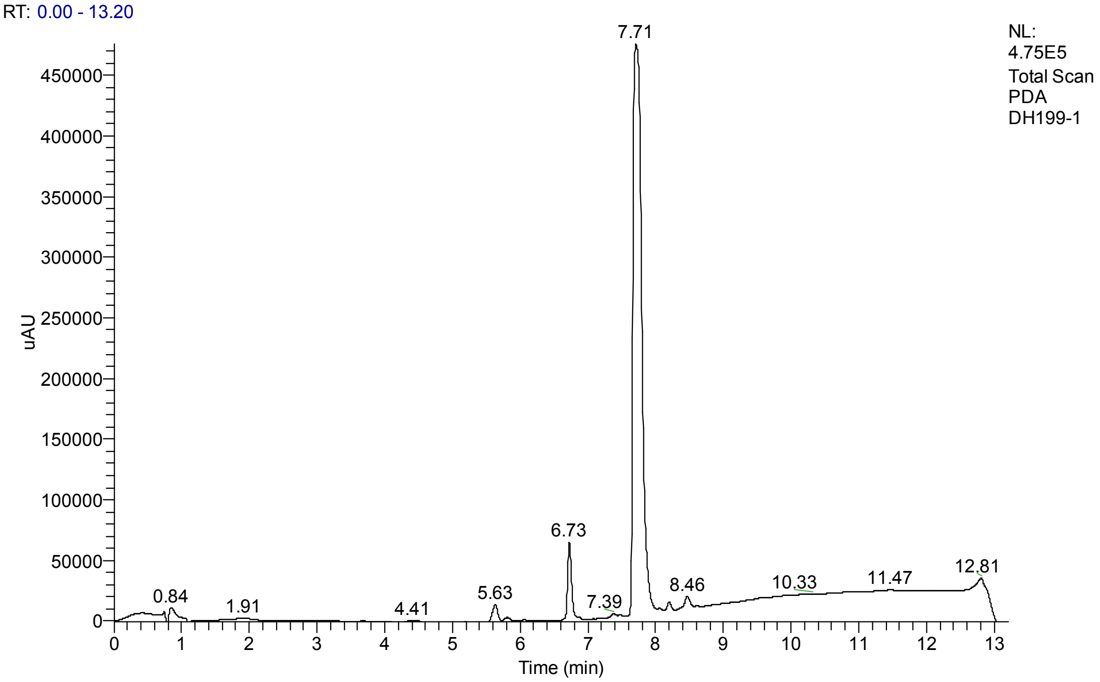

^{1}H NMR

Compound **17** ($CDCl_3$, 25 °C)

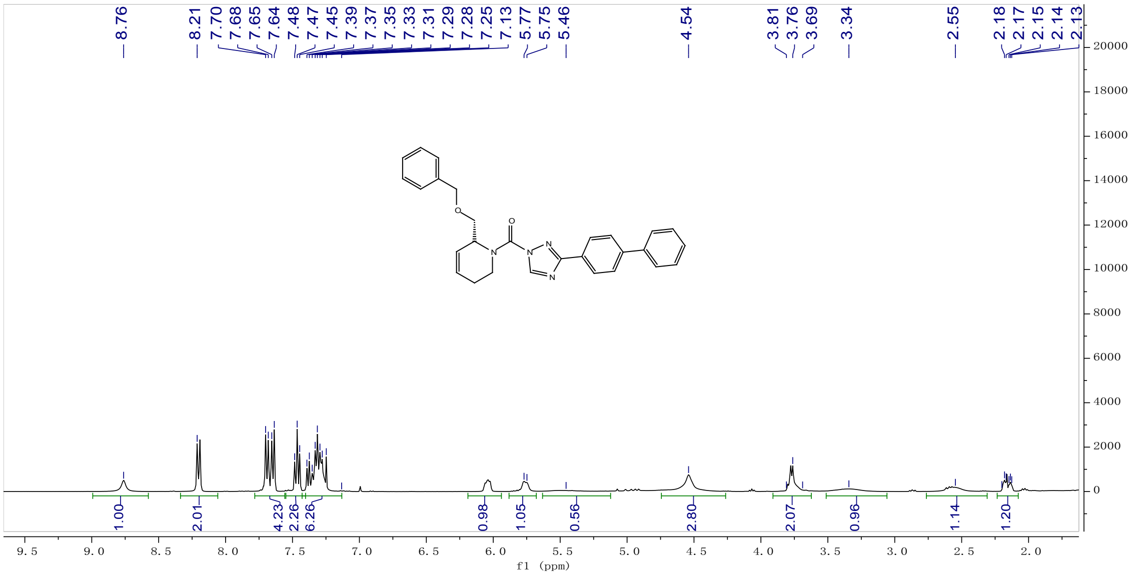

Compound **17**

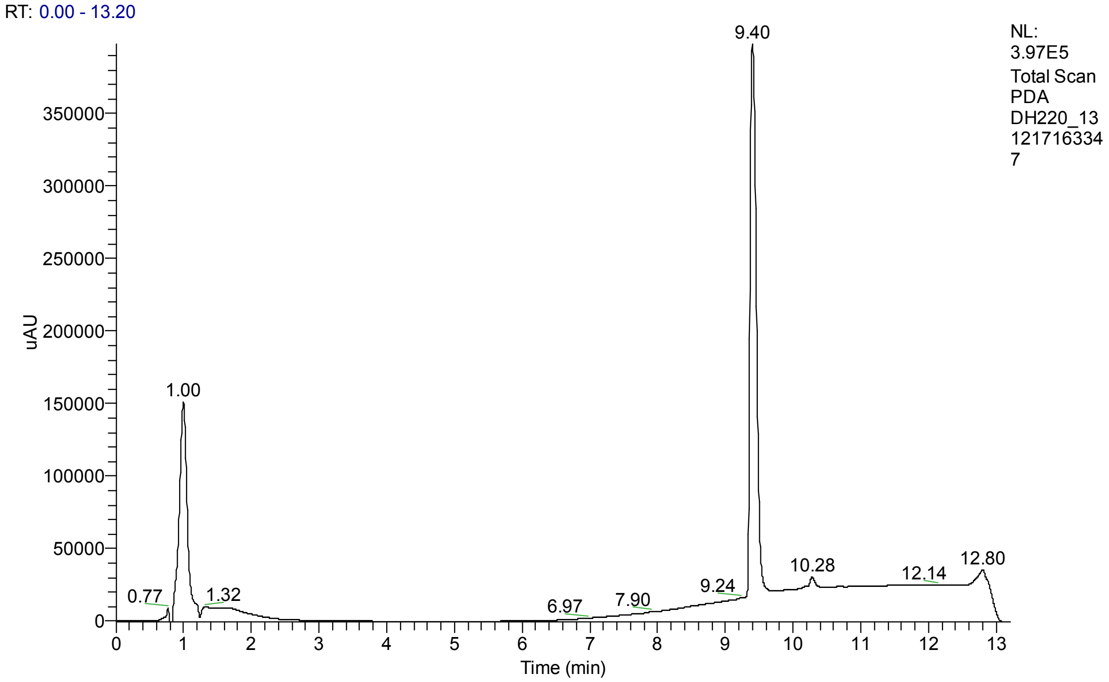

^{1}H NMR

Compound **18** ($CDCl_3$, 25 ^oC)

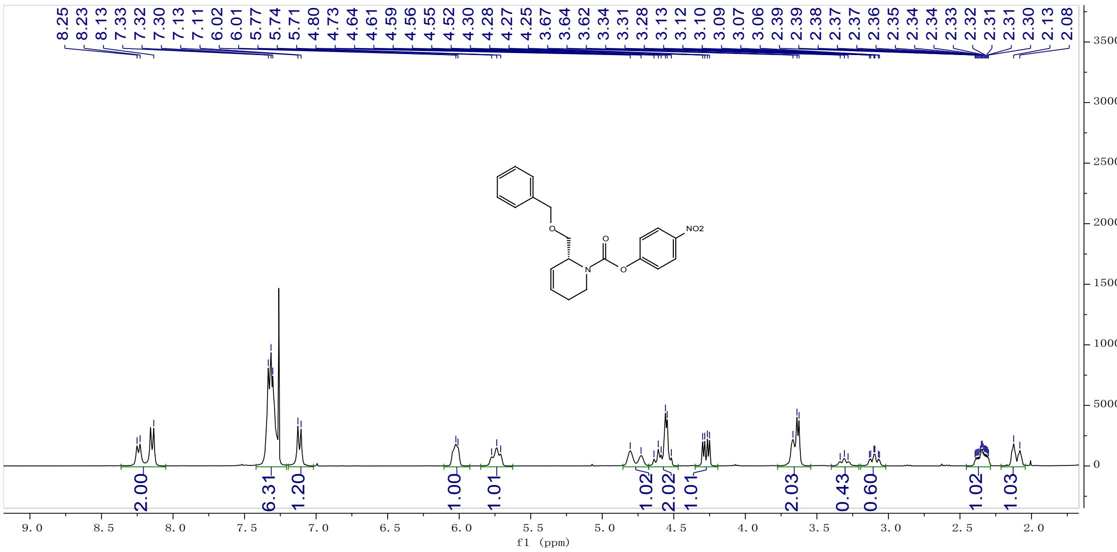

Compound **18**

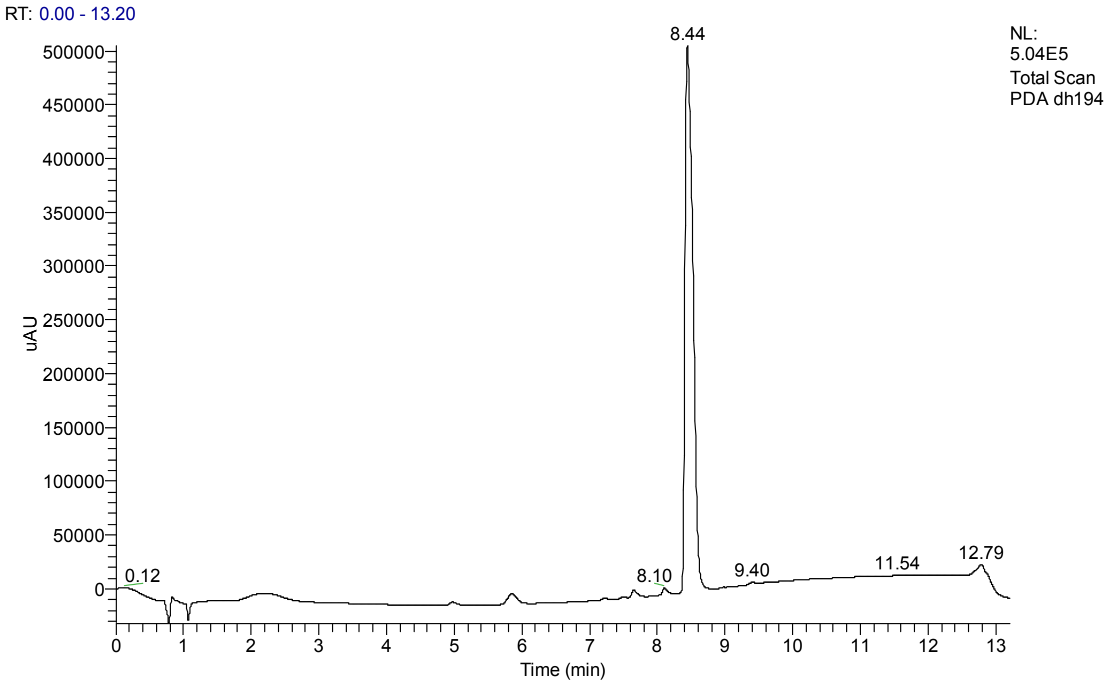

II. REFERENCES

1. Y. Banba, C. Abe, H. Nemoto, A. Kato, I. Adachi and H. Takahata, *Tetrahedron-Asymmetr*, 2001, **12**, 817-819.
2. A. M. C. H. van den Nieuwendijk, R. J. B. H. N. van den Berg, M. Ruben, M. D. Witte, J. Brussee, R. G. Boot, G. A. van der Marel, J. M. F. G. Aerts and H. S. Overkleeft, *European Journal of Organic Chemistry*, 2012, 3437-3446.
3. A. M. van den Nieuwendijk, M. Ruben, S. E. Engelsma, M. D. Risseeuw, R. J. van den Berg, R. G. Boot, J. M. Aerts, J. Brussee, G. A. van der Marel and H. S. Overkleeft, *Organic letters*, 2010, **12**, 3957-3959.
4. H. Takahata, Y. Banba, H. Ouchi and H. Nemoto, *Organic letters*, 2003, **5**, 2527-2529.
